# Hippocampal small RNAs from patients with schizophrenia induce specific cognitive and neural phenotypes in mice

**DOI:** 10.1038/s41420-026-03166-z

**Published:** 2026-05-25

**Authors:** Marcos Galán-Ganga, Anna Guisado-Corcoll, Anna Sancho-Balsells, Marina Herrero-Lorenzo, Iván Ballasch, Lisa Patterer, Jordi Alberch, Belén Arranz, Albert Giralt, Eulàlia Martí

**Affiliations:** 1 Departament de Biomedicina, Facultat de Medicina, Institut de Neurociències, Universitat de Barcelona, Barcelona, Spain; 2 Institut d’Investigacions Biomèdiques August Pi i Sunyer (IDIBAPS), Barcelona, Spain; 3 Centro de Investigación Biomédica en Red sobre Enfermedades Neurodegenerativas (CIBERNED), Madrid, Spain; 4 Production and Validation Centre of Advanced Therapies (Creatio), Faculty of Medicine and Health Science, University of Barcelona, Barcelona, Spain; 5 Parc Sanitari Sant Joan de Déu, CIBERSAM, Barcelona, Spain

**Keywords:** Genetics of the nervous system, Hippocampus

## Abstract

Schizophrenia is a neuropsychiatric syndrome characterized by the presence of psychotic, negative and cognitive symptoms. Cognitive deficits can appear even before the onset of the first psychotic episode and their severity has been recently associated with altered levels of small RNAs (sRNAs), including microRNAs. Nevertheless, how the dysregulation of sRNAs can lead to cognitive impairments and their role in the onset and pathophysiology of the disorder are still unknown. In our research, we hypothesized that hippocampal sRNAs generated in patients with schizophrenia could contribute to the impairment of specific cognitive domains that are altered in the disease. Through deep sequencing, we identified novel dysregulated sRNAs (including microRNAs and tRNA-derived fragments) in the hippocampus of patients with schizophrenia. Furthermore, when injected in the hippocampus of wild-type mice, animals exhibited an impairment in spatial short-term memory accompanied by a reduction in spine density of CA1 pyramidal neurons. RNA-sequencing of the hippocampus of injected mice revealed changes in key pathways related to neurotransmission. Specifically, we found higher levels of the presynaptic marker for Parvalbumin interneurons synaptotagmin II *(Syt2)*, mirroring observations made in the brain of schizophrenia patients. We also detected subtle morphological changes in mouse hippocampal microglia in response to sRNAs from affected individuals. Our results point out that sRNAs can contribute to cognitive symptoms in schizophrenia through synaptic alterations, highlighting these molecules as promising novel therapeutic targets for the disorder.

## Introduction

Schizophrenia is a neuropsychiatric syndrome that affects around 1% of the world population [1]. Although it is not the most prevalent mental illness, it ranks first as the leading cause of years lived with disability among patients with a diagnosis of a mental or substance use disorder [2]. Patients affected by schizophrenia typically suffer from psychotic or positive symptoms (e.g., hallucinations and delusions); negative symptoms (e.g., social withdrawal, anhedonia, and apathy); and cognitive deficits (e.g., social cognition deficits, impaired executive functions, and working memory) [3]. Currently, antipsychotic treatments can help to manage psychotic symptoms in acute episodes of schizophrenia, but they have limited benefits on cognition, and side effects might even aggravate some deficits [4]. Therefore, research and alleviation of those cognitive symptoms, that emerge in the premorbid phase of the disease and may affect almost 98% of the patients, represent an important unmet medical need [5]. Interestingly, these cognitive deficits are the first to manifest in the disorder and they are present before the onset of the first psychotic episode that typically bring patients to medical assessment, suggesting their potential as a possible early biomarker for the disorder [4].

In schizophrenia, the pathophysiology of the disease can impact cognition across 7 different domains: attention, processing speed, verbal memory, spatial memory, working memory, reasoning and problem solving, and social cognition [6]. The hippocampus is the main brain region involved in memory and cognition [7]; and different morphological, electrophysiological, synaptic and molecular abnormalities affecting this brain region have been associated to the disorder [8, 9].

Recent evidence has found altered levels of small RNAs (sRNAs) in *post-mortem* brain samples of patients affected by schizophrenia and other neuropsychiatric disorders [10]. sRNAs are non-coding RNAs with less than 200 nucleotides that do not code for proteins but play an essential role regulating mRNA expression, stability and translation; showing an important function in health and disease [10]. Among the different sRNA biotypes, microRNAs (miRNAs) have been linked to the aetiology of schizophrenia through their direct regulation of neurotransmission and immunological pathways that are known to be altered in the disease [11, 12]. Moreover, dysregulation of some miRNAs has been found not only in the hippocampus, but also in circulating blood vesicles of affected individuals in association with treatment-resistance and the severity of cognitive deficits [13]. However, none of these studies have been able to assess their active role in the onset and/or progression of the cognitive symptoms of the disease.

In the present manuscript, we aimed to decipher whether hippocampal sRNAs from patients with schizophrenia could lead to an impairment in different cognitive domains that are affected in the disease. For this purpose, we isolated sRNAs from the hippocampus of schizophrenia patients as well as control individuals to assess possible alterations in sRNAs profiles that could contribute to the complexity of altered cognition in schizophrenia. Furthermore, we generated a translational model based on the injection of these sRNAs in the hippocampus of wild-type mice to study whether species generated in schizophrenia patients have neurotoxic effects that may disrupt cognition in vivo. Behavioural and molecular changes related to an impairment in the spatial short-term memory showed that infusion of sRNAs from patients with schizophrenia in mice can mirror findings already observed in the disease as well as offer new potential therapeutic targets related to synaptic function.

## Results

### Characterization of small RNA profiles reveal new dysregulated miRNAs in the hippocampus of patients with schizophrenia

**Fig. 1:**
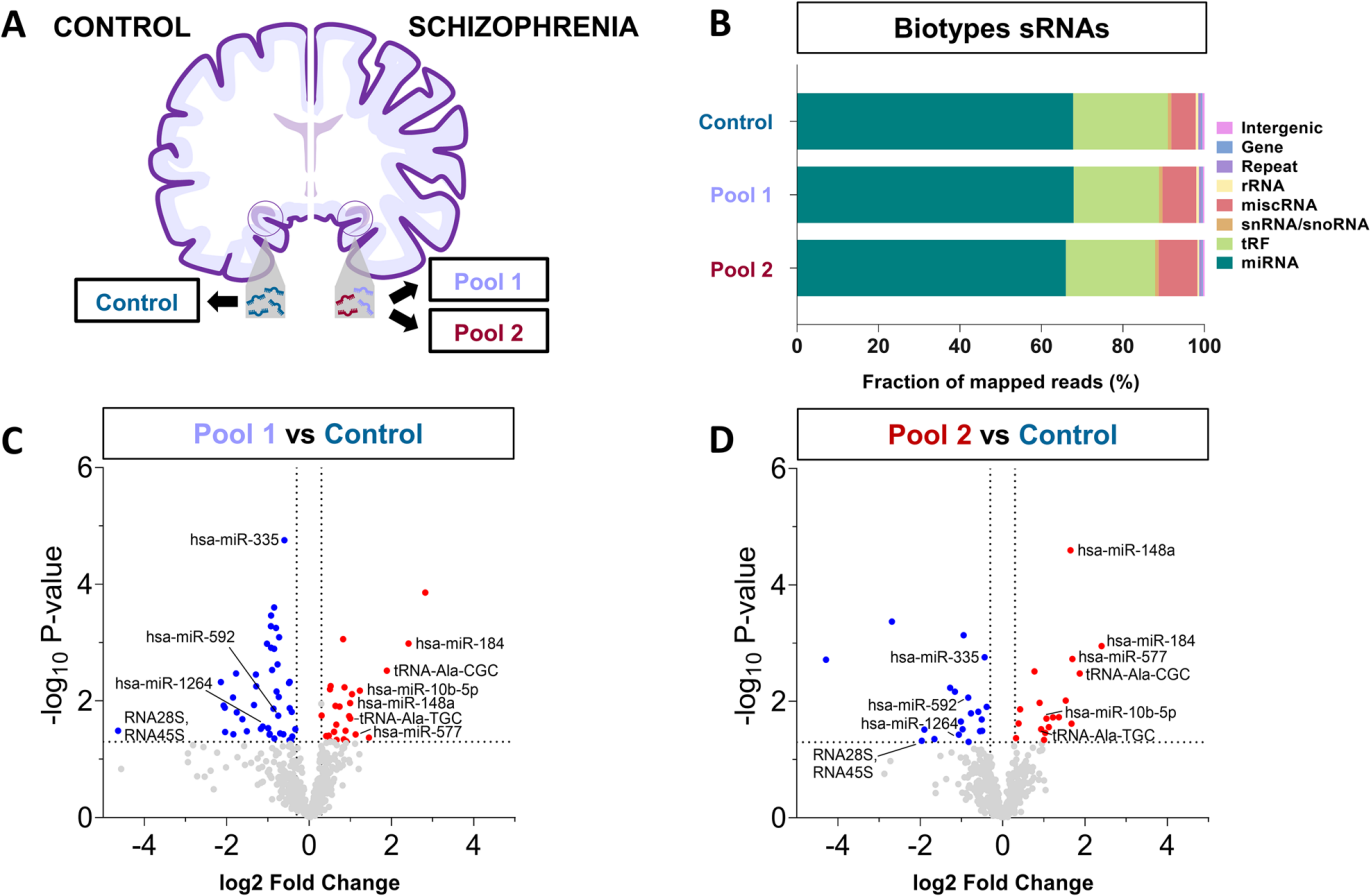
Characterization of small RNAs from *post-mortem* hippocampi of patients with schizophrenia and unaffected controls using SeqCluster annotation tool. Schematic diagram of the study design (**a**). Frozen brain hippocampal tissues from patients with schizophrenia, as well as unaffected individuals were used to purify sRNAs. sRNAs from 9 control patients were mixed into a Control Pool; whereas sRNAs from 20 patients with schizophrenia were distributed into a Pool 1 and Pool 2 of sRNAs of 10 patients each. Purified sRNAs were subjected to small RNA-sequencing. sRNAs’ biotypes using SeqCluster tool are depicted (**b**). Fraction of mapped reads in each sRNA pool (Control, Pool 1 and Pool 2) annotating onto the different sRNA biotypes (*N* = 6 patients/pool). miRNA microRNAs, tRF tRNA-derived fragments, snRNA/snoRNA small nuclear/nucleolar RNAs, miscRNA miscellaneous RNA. rRNA ribosomal RNA, Repeat repetitive RNA, Gene gene fragments, Intergenic intergenic RNAs. Volcano plots constructed with the DE sRNAs of Pool 1 vs Control (**c**) and Pool 2 vs Control (**d**) patients using the sRNA matrix of counts obtained with SeqCluster (*N* = 6 patients/pool). Red dots highlight the significantly upregulated sRNAs (Log2 Fold Change ≥ 0.3, *p*-value < 0.05) and blue dots illustrate the downregulated sRNAs (Log2 Fold Change ≤ −0.3, *p*-value < 0.05). Dots representing illustrative sRNA species are labelled.

We first aimed to characterize the profile of non-coding sRNAs from the brain of patients with schizophrenia and to compare the results with controls without psychiatric disorders. To do so we first took advantage of our cohort of human *post-mortem* hippocampal samples of patients with schizophrenia and control individuals [14]. We purified sRNAs and mixed equivalent amounts (4.7 ng) of each sample to generate a Control Pool (*N* = 9 patients) and two independent pools (Pool 1 and Pool 2) of sRNAs from patients with schizophrenia (*N* = 10 patients per pool) (Fig. 1a).

sRNAs from a representative group of individuals from each pool (*N* = 6) were sequenced and reads were quantified and annotated SeqCluster [15] and ExceRpt [16] bioinformatic tools. SeqCluster tool showed that miRNAs were the most abundant biotype (65–67%) followed by tRNA derived fragments (tRFs) (20–23%) and miscellaneous RNAs (miscRNA) (5–9%) (Fig. 1b). No significant changes in the proportion of each of the sRNA biotypes were found between the control samples and the two different pools of schizophrenia patients. Similar abundances of sRNA biotypes were obtained when using the ExceRpt tool (Supplementary Fig. 1).

Profiting from the advantages of SeqCluster to annotate complex sRNA sequences, such as tRFs, we first performed a differential expression (DE) analysis using the sRNA count matrix generated with this tool to assess which specific sRNA biotypes were more dysregulated in Pool 1 and Pool 2 of patients with schizophrenia compared to controls. Interestingly, we found that the majority of dysregulated sRNAs (Log2 Fold Change ≥ 0.3 and ≤ −0.3, *p*-value < 0.05) corresponded to miRNAs, although we also identified some tRFs and rRNA fragments dysregulated (Fig. 1c, d) (Supplementary Table 3A, B). This observation adds to the recent evidence suggesting that altered levels of miRNAs in the brain [12] and circulating extracellular vesicles (EVs) [13] of patients with schizophrenia might be related to the onset and/or severity of the disease. Thus, we focused on analyzing miRNA candidates in our samples using ExceRpt, a tool that optimally annotates these sequences [16].

**Fig. 2:**
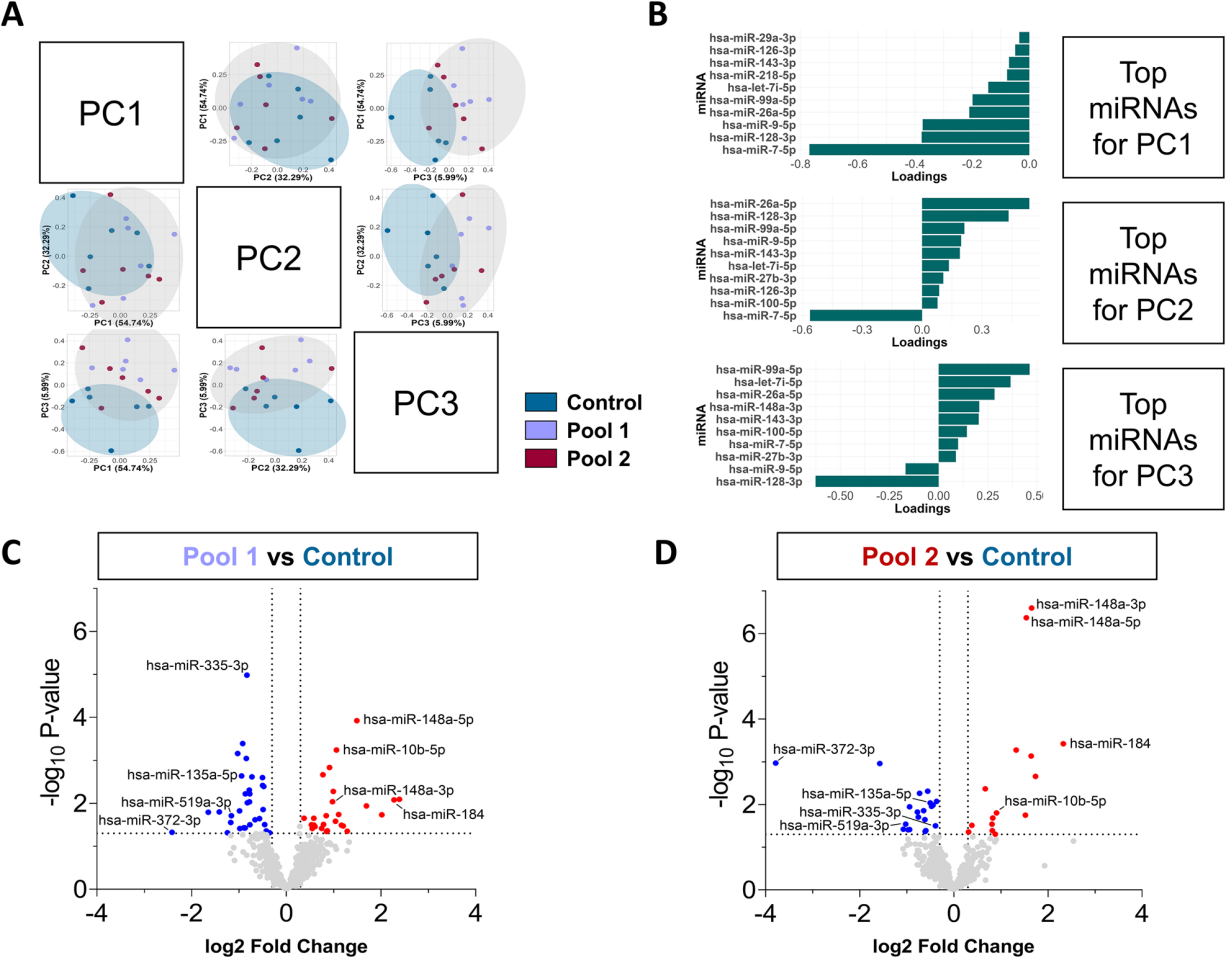
Characterization of microRNAs from *post-mortem* hippocampi of patients with schizophrenia and unaffected controls using ExceRpt annotation tool. miRNA PCA plots (**a**). PCA plots constructed with the matrix of counts of only miRNAs obtained using ExceRpt tool and combining the first three components that explain more than 90% of the variance (*N* = 6 patients/pool). miRNA PC loadings (**b**). PC loadings of the top 10 miRNAs species contributing to the separation of control and schizophrenia samples in each of the three components. Volcano plots constructed with the DE miRNAs of Pool 1 vs Control (**c**) and Pool 2 vs Control (**d**) patients using the miRNA matrix of counts obtained with ExceRpt. Red dots highlight the significantly upregulated miRNAs (Log2 Fold Change ≥ 0.3, *p*-value < 0.05) while blue dots illustrate the downregulated miRNAs (Log2 Fold Change ≤ -0.3, *p*-value < 0.05). Dots representing illustrative miRNA species are labelled.

We performed an unsupervised principal component analysis (PCA) to look for specific expression patterns of miRNAs discriminating groups using the miRNAs identified with ExceRpt. The first two components of the PCA did not show a clear separation between controls and patients with schizophrenia (Pool 1 and Pool 2). However, the first and third components of the PCA provided better discrimination between unaffected individuals and diseased patients (Fig. 2a). The top 10 miRNAs species contributing to the separation of control and schizophrenia samples in each of the 3 components were identified and the respective loadings are summarized in Fig. 2b.

To go deep into the possible association of specific miRNA candidates and the disease, we did a DE analysis between controls and schizophrenia patients (Pool 1 and Pool 2) using the miRNA count matrix generated by the ExceRpt tool. As it can be observed in the volcano plots, we found a total of 26 and 15 upregulated miRNAs for Pool 1 and Pool 2 patients compared to controls (Log2 Fold Change ≥ 0.3, *p*-value < 0.05), respectively (Fig. 2c); and a total of 32 and 20 downregulated miRNAs for Pool 1 and Pool 2 patients compared to controls (Log2 Fold Change ≤ -0.3, *p*-value < 0.05), respectively (Fig. 2d) (Supplementary Table 4A–D). Equivalent results were obtained when using the SeqCluster tool (Supplementary Fig. 2a–d) (Supplementary Table 5a–d).

**Fig. 3:**
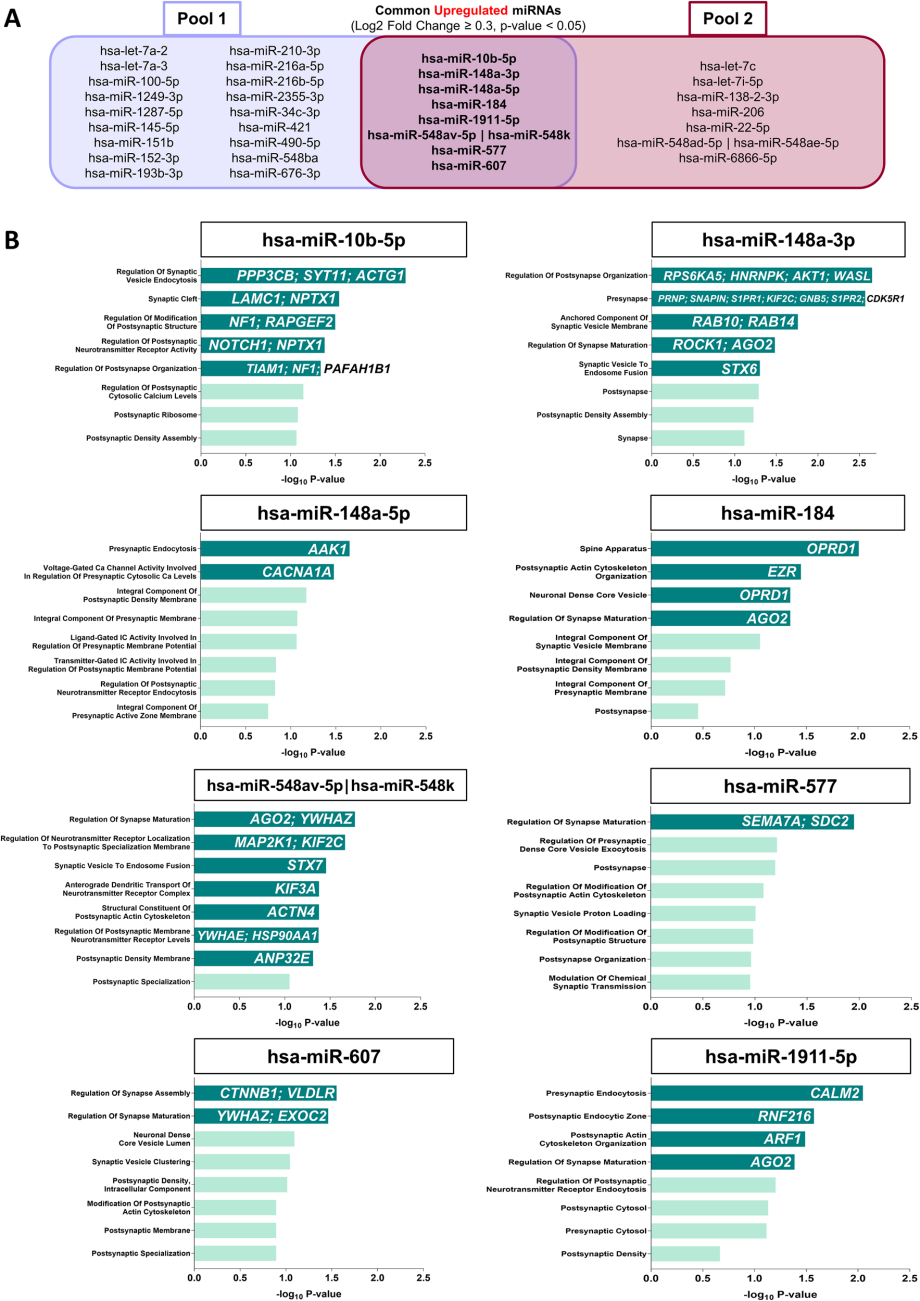
Gene ontology synaptic pathways associated to the target genes of common upregulated microRNAs in the hippocampus of Pool 1 and Pool 2 patients using SynGO tool. Venn diagram showing the overlap between significantly upregulated miRNAs detected in both sRNA pools of patients with schizophrenia (**a**). Screening threshold used was Log2 Fold Change ≥ 0.3 and *p*-value < 0.05 for both Pool 1 and Pool 2 groups of patients. Bar plots showing -log10 *p*-values of representative gene ontology synaptic pathways associated to the target genes of common upregulated microRNAs (Log2 Fold Change ≥ 0.3, *p*-value < 0.05) in Pool 1 and Pool 2 of patients with schizophrenia (**b**). Target genes of each miRNA were obtained from the mirTarBase database and introduced into the SynGO 2024 for gene ontology analysis. Pathways with a significant *p*-value (*p*-value < 0.05) are depicted in dark green and associated candidate genes are indicated in black/white. Non-significant pathways (*p*-value ≥ 0.05) are depicted in light green.

Our experimental paradigm is based on the injection of human sRNAs into the brains of wild-type mice. This approach suggests that the functional effects of schizophrenia sRNAs in the hippocampus of mice would be driven by species specifically produced and therefore over-represented in this condition, rather than the absence or under-representation of specific sRNA sequences. This is the reason why, for our analysis, we decided to focus on upregulated miRNAs. We found that 8 miRNAs were commonly upregulated in both Pool 1 and Pool 2 of patients: hsa-miR-10b-5p, hsa-miR-148a-3p, hsa-miR-148a-5p, hsa-miR-184, hsa-miR-1911-5p, hsa-miR-548av-5p | hsa-miR-548k, hsa-miR-577, and hsa-miR-607 (Fig. 3a).

Given that synaptic abnormalities affecting the hippocampus have been associated with schizophrenia [8, 9], we wondered whether those common upregulated miRNAs in Pool 1 and Pool 2 of patients might regulate gene pathways related to the organization and functioning of brain synapses. As described in the Materials and Methods section, we obtained the target genes of each of those miRNAs from the mirTarBase [17] and introduced them in the SynGO web server [18] to explore the functional annotations associated to synaptic pathways.

Target genes of common upregulated miRNAs in both Pool 1 and Pool 2 were significantly involved (p-value < 0.05) in one or more synaptic pathways (Fig. 3b). Gene ontology analysis revealed pathways related to the regulation of pre and postsynaptic organization, synapse assembly, and synapse maturation, among others. Interestingly, altered levels and specific variants of some of the genes involved in those processes, such as *AGO2*, *AKT1*, *CTNNB1* and *YWHAZ*, have been previously associated with schizophrenia [19–23].

Altogether, these data suggest that the combined action of multiple dysregulated sRNAs rather than a single clear sRNA candidate in the hippocampus of patients with schizophrenia might induce pathogenic alterations contributing to the symptomatology of the disorder.

### Injection of small RNAs from patients with schizophrenia provokes specific hippocampal-related cognitive deficits in mice

**Fig. 4:**
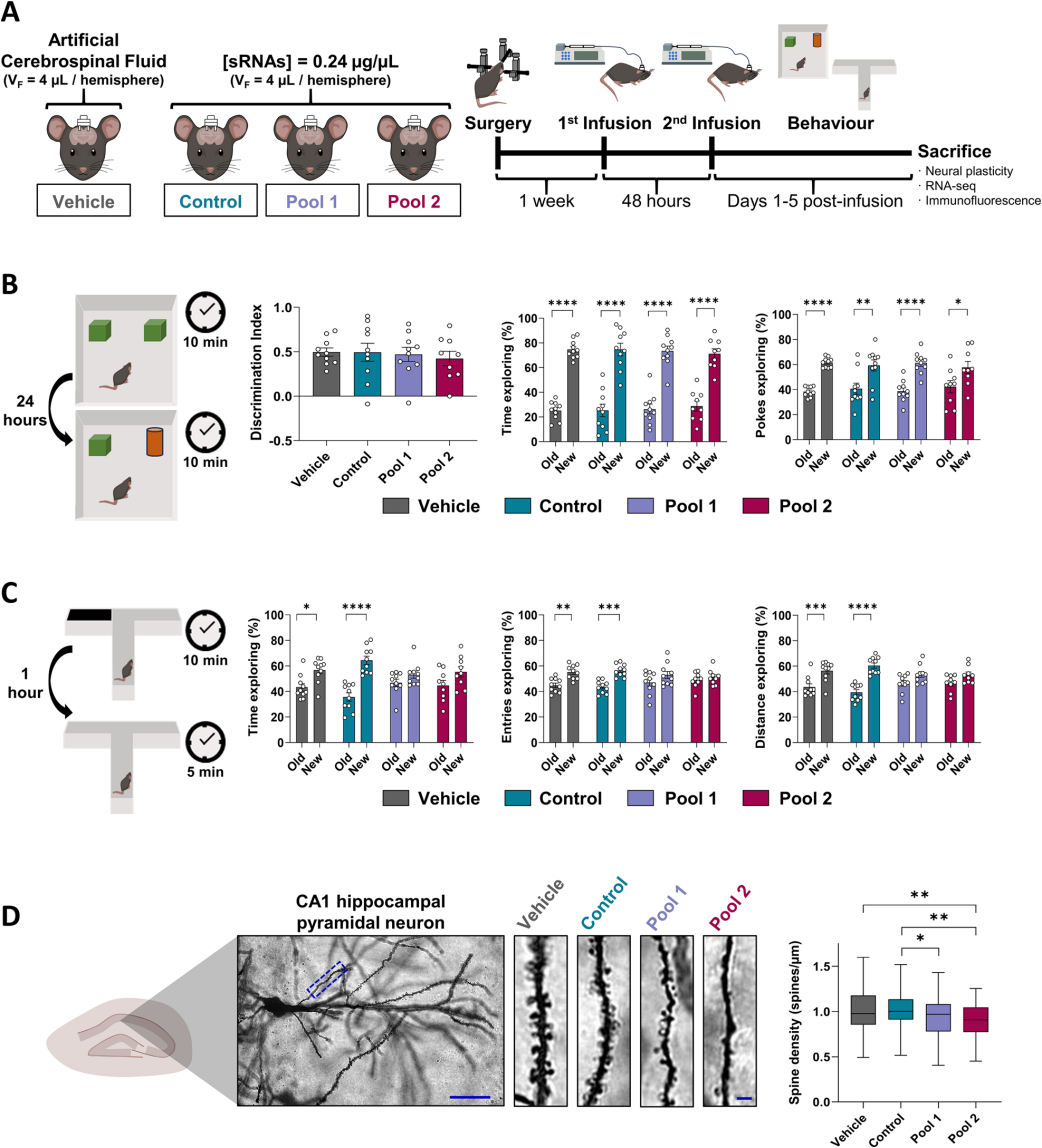
Behavioral effects induced by intra-hippocampal infusion of small RNAs from *post-mortem* hippocampi of patients with schizophrenia and unaffected controls. Schematic diagram of the study design (**a**). sRNAs were injected into the dorsal hippocampus of wild-type mice using bilateral cannulas. Artificial cerebrospinal fluid was used as an additional control of the experiment. Injected mice were assessed for different cognitive skills. Four groups were generated: Vehicle group (no sRNAs injected), Control group (injected with sRNAs from controls with no psychiatric illnesses), Pool 1 group (injected with sRNAs from a group of 10 patients with schizophrenia) and Pool 2 group (injected with sRNAs from a second and different group of 10 patients with schizophrenia). Once sacrificed, brains were processed for histopathological evaluation (Golgi staining), transcriptomic analysis (RNA-seq) and immunofluorescence. Results from recognition memory assessment in the novel object recognition test (NORT) (**b**). Three parameters assessing the preference for the new object were analyzed (from left to right): Discrimination Index (first panel, one-way ANOVA, F(_3,35_) = 0.1727, *P* = 0.9141, no effects), percentage of time exploring the objects (second panel, two-way ANOVA, novelty effect F(_1,70_) = 279.2, *P* < 0.0001; interaction effect F(_3,70_) = 0.3454, *P* = 0.7926) and percentage of number of nose pokes (third panel, two-way ANOVA, novelty effect F(_1,70_) = 63.60, *P* < 0.0001; interaction effect F(_3,70_) = 0.5112, *P* = 0.6759). Results from the spatial memory assessment in a T-maze (**c**). Three parameters assessing the preference for the new arm (context) were analyzed (from left to right): percentage of time exploring arms (first panel, two-way ANOVA, novelty effect F(_1,70_) = 41.10, *P* < 0.0001; interaction effect F(_3,70_) = 4.533, *P* = 0.0057), percentage of number of entries into the arms (second panel, two-way ANOVA, novelty effect F(_1,70_) = 28.48, P < 0.0001; interaction effect F(_3,70_) = 2.609, *P* = 0.0583) and percentage of travelled distance (third panel, two-way ANOVA, novelty effect F(_1,70_) = 54.87, P < 0.0001; interaction effect F(_3,70_) = 4.558, P = 0.0056). Tukey’s and Bonferroni’s *post hoc* test were used accordingly, *P < 0.05, **P < 0.01, ***P < 0.001 and ****P < 0.0001. Values are mean ± SEM. *N* = 9–10 mice/group. Golgi staining was performed in brains from mice of the four groups (Vehicle, Control, Pool 1 and Pool 2) (**d**). Second order apical dendrites from CA1 pyramidal neurons were evaluated for spine density and a representative dendrite from each group is depicted. Spine density (spines/micron) is shown for each group (one-way ANOVA, F(_3,389_) = 6.890, P = 0.0002). Tukey’s *post hoc* test was performed, *P < 0.05 and **P < 0.01. Values are mean ± SEM. 84–113 dendrites from 5 mice/group were analyzed. Scale bar representative neuron: 25 microns. Scale bar representative dendrites: 2 microns.

Given the important role of miRNAs as modulators of gene expression and function in the brain [24, 25], we then aimed to determine the role of sRNAs in vivo by directly injecting them into the hippocampus of wild-type mice following our previously described procedure [26]. Based on the molecular study carried out in Fig. 1a, we generated four experimental groups (Fig. 4a): mice injected with sRNAs pooled from 9 control individuals (Control), mice injected with sRNAs pooled from 10 patients with schizophrenia (Pool 1) and mice injected with sRNAs pooled from a second cohort of 10 patients with schizophrenia (Pool 2). Artificial cerebrospinal fluid was used as an additional vehicle control in a fourth group of mice (Vehicle). These groups of mice were bilaterally infused in the dorsal hippocampus with the sRNAs (or vehicle) two times (Fig. 4a). 24 hours after the last infusion all mice were subjected to a battery of behavioral tests such as open field test to monitor anxiety-like and exploratory behaviours, novel object recognition test (NORT) to evaluate recognition memory, and T-maze test to assess spatial memory.

First, body weight of the four groups of mice were indistinguishable (Supplementary Fig. 3) as well as their appearance (data not shown) indicating no gross affectation of the mice health due to this experimental approach. Next, evaluation of the open field data also indicated that all groups of mice displayed normal anxiety-like levels as well as unaffected locomotor activity and exploration when compared with the vehicle group (Supplementary Fig. 4). In the NORT, all groups of mice displayed normal recognition memory skills determined by several parameters such as the Discrimination Index, total time exploring the objects and number of nose pokes (Fig. 4b). Finally, in the T-Maze test, both Vehicle and Control groups showed increased preference to explore the new arm as evaluated using several parameters such as time exploring the new arm, number of times entering the new arm as well as distance travelled in the new arm (Fig. 4c). However, both groups that received the sRNAs from patients with schizophrenia (Pool 1 and Pool 2 groups) showed a clear and consistent impaired performance as observed in all parameters evaluated (Fig. 4c). Since mouse hippocampal-related cognitive skills seem to be susceptible to sRNAs from patients with schizophrenia, we next evaluated micro-structural synaptic plasticity in their hippocampi (Fig. 4a). To do so we quantified the spine density in the apical dendrites of CA1 pyramidal neurons (Fig. 4d). Interestingly, no differences were observed between Vehicle and Control groups suggesting no affectations by the mere fact of injecting sRNAs in the mouse hippocampus (Fig. 4d). In contrast, Pool 1 and Pool 2 groups both showed a significant reduction of spine density in apical dendrites of CA1 pyramidal neurons when compared with the Control group (Fig. 4d). Moreover, sRNAs injection at the desired coordinates (Supplementary Fig. 5a) was confirmed in hippocampal sections stained with DAPI (Supplementary Fig. 5b). Since we did not find any significant differences in the behavior and the spine density between the Vehicle and Control mice, for downstream analyses we decided to focus on the comparison between mice receiving the sRNAs from Control individuals and sRNAs from Pool 1 and Pool 2 of patients.

Altogether, these results indicated that injecting sRNAs in the mouse hippocampus following our protocol is not toxic per se and that sRNAs from patients with schizophrenia induce specific spatial memory deficits with associated changes on structural synaptic plasticity.

### Small RNAs from patients with schizophrenia induce alterations in the expression pattern of hippocampal synaptotagmin II, mirroring molecular changes present in the brain of these patients

Since we observed specific spatial memory deficits in mice injected with sRNAs from patients with schizophrenia, we then performed a broad characterization of the molecular changes taking place by subjecting the mice hippocampi to RNA sequencing.

**Fig. 5:**
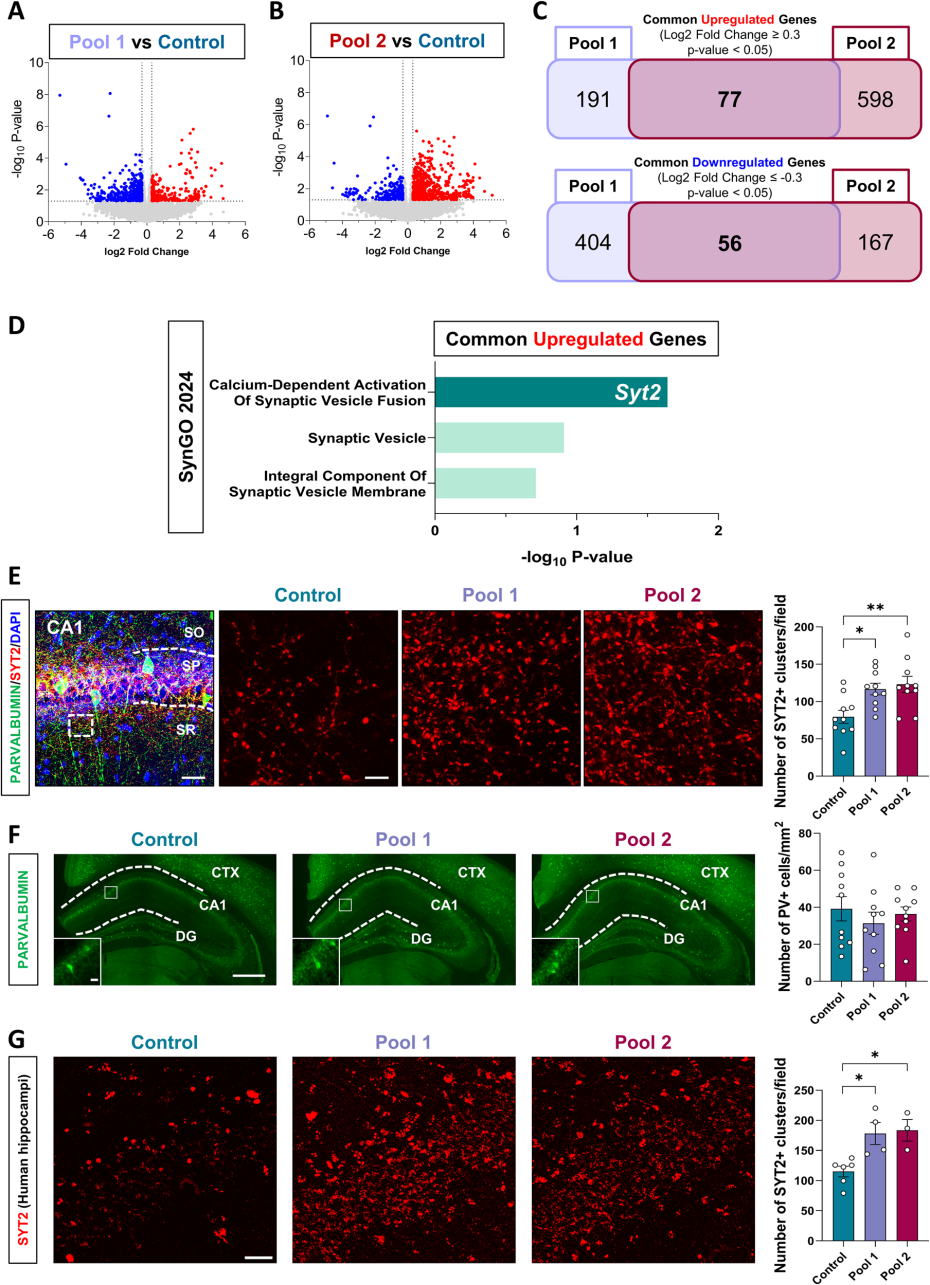
Molecular effects in mice mediated by intra-hippocampal infusion of sRNAs from *post-mortem* hippocampi of patients with schizophrenia and unaffected controls. RNA-seq analysis of the hippocampus of mice injected with sRNAs. Volcano plots constructed with DEGs in the hippocampus of Pool 1 vs Control (**A**) and Pool 2 vs Control (**B**) mice. Red dots highlight the significantly upregulated miRNAs (Log2 Fold Change ≥ 0.3, *p*-value < 0.05) while blue dots illustrate the downregulated miRNAs (Log2 Fold Change ≤ -0.3, *p*-value < 0.05). Venn diagrams showing the overlap between significantly upregulated and downregulated genes detected in both groups of mice injected with sRNAs from patients with schizophrenia (**C**). Screening thresholds used were Log2 Fold Change ≥ 0.3 and Log2 Fold Change ≤ -0.3, respectively; and *p*-value < 0.05for both Pool 1 and Pool 2 groups of mice. Gene ontology synaptic pathways and gene candidates associated to common upregulated genes in the hippocampus of Pool 1 and Pool 2 mice (**D**). Bar plot showing -log10 *p*-values of representative gene ontology synaptic pathways associated with the common upregulated genes (Log2 Fold Change ≥ 0.3, *p*-value < 0.05) in Pool 1 and Pool 2 groups of mice using SynGo 2024. Pathways with a significant p-value (p-value < 0.05) are depicted in dark green and associated candidate genes are indicated in white. Non-significant pathways (p-value ≥ 0.05) are depicted in light green. Characterization of hippocampal SYT2-positive pre-synaptic clusters of inhibitory synapses in mice injected with Control, Pool 1 or Pool 2 sRNAs (**E**). Left panel illustrates the number of SYT2-positive pre-synaptic clusters (in red) in the dorsal CA1 (as shown by the DAPI nuclei staining in blue) and colocalizing with parvalbumin (in green). White dashed square shows that SYT2-positive clusters were analyzed in the CA1 *stratum radiatum* (SR) of the three groups of treated mice (Control, Pool 1 and Pool 2 groups). SO *stratum oriens*. SP *stratum pyramidale*. Scale bars: 40 and 4 microns, respectively. Bar plot shows the number of SYT2-positive clusters/image in the three groups of mice (right panel, one-way ANOVA, F(_2,27_) = 6.653, *P* = 0.0045). Number of parvalbumin-positive cells (in green) were quantified in the dorsal CA1 of the three groups (**F**). In the left panels, representative images are depicted. Scale bar: 500 microns for tile scans and 25 microns for zoom ins. Quantification of parvalbumin cells was determined and relativized per area (right panel, one-way ANOVA, F(_2,27_) = 0.5038, *P* = 0.6098). CTX cortex, DG dentate gyrus. In (**E**, **F**), Tukey’s *post hoc* test was performed, *P < 0.05 and **P < 0.01. Values are mean ± SEM. At least two slices were analysed per mouse and *N* = 10 mice/group. Characterization of SYT2-positive clusters in the hippocampus of Control, Pool 1 and Pool 2 patients (**G**). Representative images illustrating the number of SYT2-positive clusters (in red) are depicted (scale bar: 30 microns). Bar plot shows the number of SYT2-positive clusters/image in each group of patients (right panel, one-way ANOVA, F(_2,10_) = 8.394, *P* = 0.0073). Tukey’s *post hoc* test was performed, *P < 0.05. Values are mean ± SEM. One slice per patient and at least two images per slice were analyzed. *N* = 3–6 patients/group.

First, we analyzed the DEGs in Pool 1 and Pool 2 mice compared to those mice receiving sRNAs from the Control group. As summarized in the volcano plots, we found a total of 268 and 675 upregulated genes in the hippocampus of Pool 1 and Pool 2 mice (Log2 Fold Change ≥ 0.3, *p*-value < 0.05), respectively (Fig. 5a); and a total of 460 and 223 downregulated genes in the Pool 1 and Pool 2 mice (Log2 Fold Change ≤ −0.3, *p*-value < 0.05), respectively (Fig. 5b) (Supplementary Table 6a–d).

Since spatial memory deficits and spine density loss were commonly observed in both Pool 1 and Pool 2 groups of mice (Fig. 4c, d), we focused our analysis on potential dysregulated genes that might be related to synaptic changes. Among the commonly 77 upregulated and 56 downregulated genes shared by both Pool 1 and Pool 2 mice (Fig. 5C) (Supplementary Table 7A, B), gene ontology analysis using the SynGO web server [18] allowed us to identify synaptotagmin II gene (*Syt2*) as a significant (*p*-value < 0.05) upregulated candidate gene related with calcium-dependent activation of synaptic vesicle fusion (Fig. 5D).

We aimed to verify and further explore the changes in SYT2 protein expression by immunofluorescence in fixed hippocampal slices from mice of the three groups. It is noteworthy that SYT2 is a presynaptic marker for inhibitory projections coming from Parvalbumin interneurons [27, 28] and regulates the GABA release capacity of these synaptic terminals [29]. Therefore, using confocal microscopy we quantified SYT2-positive clusters in the *Stratum Radiatum* of the CA1 (Fig. 3c). The number of clusters was significantly increased in both Pool 1 and Pool 2 groups, when compared with Control group (Fig. 5E). These changes were independent of the total number of Parvalbumin interneurons in CA1 (Fig. 5F). Then, we wanted to validate whether those changes in the number of SYT2-positive clusters were also present in the hippocampus of patients used for sRNA extraction. Immunofluorescence of a representative number of patients from each group showed that schizophrenia patients from both Pool 1 and Pool 2 groups also had a significant increase in the number of clusters, compared with the Control individuals (Fig. 5G).

Since SYT2 levels can be linked to pathologies affecting the central nervous system [30], we believe its upregulation could explain, at least in part, the hippocampal related memory deficits observed in sRNA-schizophrenia injected mice and this approach might recapitulate some of the molecular changes that are present in the brain of patients with schizophrenia.

### Injection of small RNAs from patients with schizophrenia generates subtle but consistent changes in mouse hippocampal microglia

**Fig. 6:**
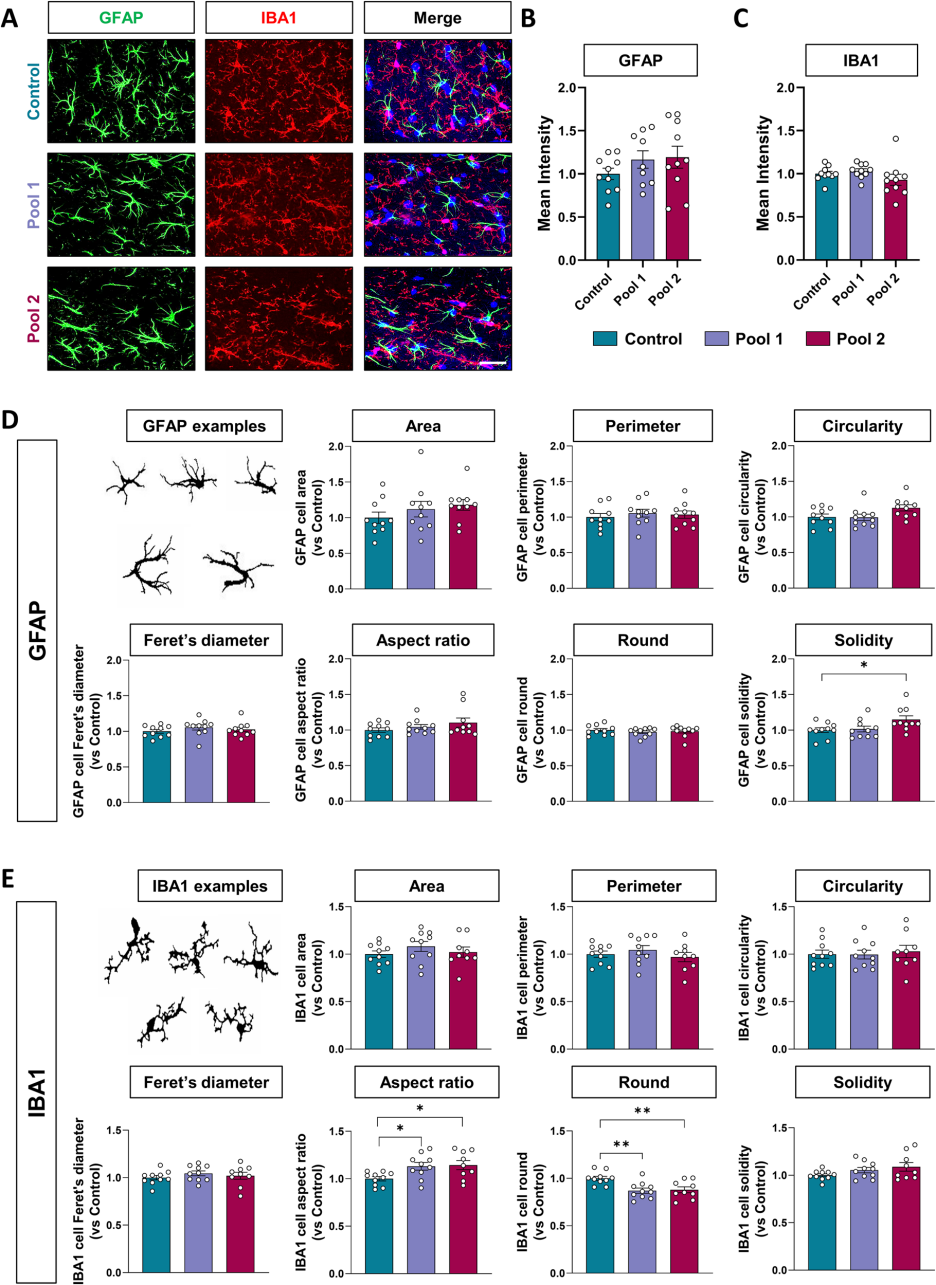
Effects of small RNA injection on mice hippocampal inflammatory markers. Representative images of GFAP staining (in green) and IBA1 (in red) co-stained with the nuclei marker DAPI (blue) in the *stratum radiatum* of the hippocampus in each group of mice (Control, Pool 1 and Pool 2) (**a**). Mean intensity (IOD) of GFAP-positive astrocytes (**b**) and IBA1-positive microglia (**c**) in the *stratum radiatum* of the hippocampus. Several cellular shape descriptors for astrocytes (**d**) and microglia (**e**) included by default in the FIJI software were analyzed and relativized to Control group: area, perimeter, circularity, Feret’s diameter, aspect ratio, round and solidity as well as some examples of different types of GFAP and IBA1-positive cells are also depicted. (GFAP solidity, one-way ANOVA, F(_2,27_) = 3.660, *P* = 0.0392; IBA1 aspect ratio, one-way ANOVA, F(_2,26_) = 4.408, *P* = 0.0225; IBA1 round, one-way ANOVA, F(_2,26_) = 7.389, *P* = 0.0029). Dunnet’s *post hoc* test comparing both Pool 1 and Pool 2 with Control mice was performed, *P < 0.05 and **P < 0.01. Values are mean ± SEM. At least two slices per mouse and two images per slice were analyzed. *N* = 9, 10 mice/group.

Bulk RNAseq analysis of mice hippocampi indicated no major changes in molecular profiles related with neuroinflammation or with glial function upon injection with sRNAs from patients with schizophrenia. However, since neuroinflammation has been suggested to participate in schizophrenia [31], we cannot rule out possible subtle changes in the state, morphology and/or function of astroglia and microglia since they are very fluent, heterogenous and complex [32, 33]. Thus, we performed a characterization of astroglia and microglia morphology following up a previously published pipeline established by our group [34]. First, hippocampal levels of GFAP and IBA1 (both are classical markers for astrocytes and microglia respectively) were indistinguishable between Pool 1 and Pool 2 groups in respect to the Control group (Fig. 6a–c). Next, we evaluated the morphology of GFAP-positive astrocytes located in the *Stratum Radiatum* of the CA1 hippocampal region. We focused on the following parameters: area, perimeter, circularity, Feret’s diameter, aspect ratio, round and solidity (Fig. 6d). We found a subtle increase in solidity (less ramifications) only in Pool 2 astrocytes compared with the Control group. Similarly, we evaluated the same eight parameters in microglia also located in the *Stratum Radiatum* of the CA1 (Fig. 6e). Interestingly, we found subtle but consistent changes namely increased aspect ratio and decreased round in microglia from both groups, Pool 1 and Pool 2, when compared to the Control group (Fig. 6e). Altogether these results suggest that mouse microglia, although in a subtle way, react to the injection of sRNAs specifically coming from patients with schizophrenia increasing their polarization and elongation, while they remain apparently unaltered in the presence of sRNAs from healthy individuals.

## Discussion

In this manuscript, we present for the first time how sRNAs isolated from *post-mortem* human hippocampi affected by schizophrenia impair specific declarative memory skills in mice, whereas sRNAs from control individuals do not. We identified several sRNA species that may substantially contribute to these deficits, and we describe how inhibitory presynaptic contacts of parvalbumin interneurons are particularly affected by these sRNAs from schizophrenia patients.

To date, broad changes in sRNA profiles in patients with schizophrenia have been largely descriptive and primarily focused on analyzing the levels of specific miRNAs in other brain regions different from the hippocampus and in circulating EVs isolated from plasma. Specifically, several sRNAs have been reported as altered in circulating EVs correlating with the severity of cognitive deficits [13]. Building on this, we have here evaluated whether sRNAs generated in the hippocampus of schizophrenia patients contribute to cognitive alterations.

We identified a range of differentially expressed sRNAs in patients with schizophrenia compared to control individuals. Although most of them corresponded to miRNAs, our analysis revealed for the first time alterations in other sRNA biotypes that might be of interest to the pathophysiology of the disorder. For instance, we found upregulated levels of tRNA-Ala-CGC and tRNA-Ala-TGC derived tRFs. Interestingly, both tRFs appear to be accumulated in the senescence-accelerated mouse prone 8 (SAMP8) model, which is used as a model of accelerated ageing and shows early learning and memory deficits [35]. This is particularly relevant, since life expectancy in schizophrenia is reduced up to 15 years [36].

Focusing on miRNAs, we observed that hsa-miR-10b-5p, hsa-miR-148a-3p, hsa-miR-148a-5p, hsa-miR-184, hsa-miR-1911-5p, hsa-miR-548av-5p, hsa-miR-548k, hsa-miR-577, and hsa-miR-607 were upregulated in the hippocampus of the two different pools of schizophrenia patients compared to controls. Among them, hsa-miR-148a-3p was also identified as one of the top contributors for separation in the third component of PCA, which provided a better discrimination between unaffected individuals and diseased patients. Remarkably, hsa-miR-148a-3p is enriched in neurons and increased levels of this miRNA have been found in plasma from patients with Alzheimer’s disease and a longitudinal mouse model for age-associated memory decline [37, 38]. Bath application of miR-148a-3p in mouse primary hippocampal neurons induced a decrease in spine density while injection of inhibitory oligonucleotides against this miRNA in the CA1 region of old mice was able to prevent hippocampal-dependent age-associated memory deficits [38]. Furthermore, hsa-miR-148a-5p has been associated with cognitive function in humans [39, 40].

Another potential candidate contributing to cognitive impairment would be hsa-miR-10b-5p. Perinatal adverse environments increase the levels of this miRNA [41]. Research in Huntington’s disease has also revealed increased levels of hsa-miR-10b-5p in the PFC of patients with comorbid psychiatric traits [40]. Moreover, the hsa-miR-184, has been shown to be altered in Major Depressive Disorder and correlated with memory impairment [42]. We also observed increased levels of hsa-miR-548k, which has been associated with cognitive impairment and Alzheimer’s disease [43] probably involving microglia-related dysfunctions [44, 45]. In a recent study [46], the authors sequenced sRNAs from the frontal cortex of schizophrenia patients and identified hsa-miR-99b-5p as a potential biomarker candidate. They demonstrated that inhibition of hsa-miR-99b-5p in the prefrontal cortex of mice induced alterations in the Pre-Pulse Inhibition (PPI) paradigm, a model for positive symptoms in schizophrenia [47]. As it could have been expected due to specific regional expression of miRNAs inside the brain, we did not observe changes in hsa-miR-99b-5p in the hippocampus. Nevertheless, we identified hsa-miR-99a-5p as one of the top contributors in the PCA. This miRNA has been linked to cognitive impairments related to reversal learning in mice [48]. Although differential expression of hsa-miR-99a-5p in the hippocampus of both pools of schizophrenia patients did not reach statistical significance, the fact that this miRNA shares the seed region with hsa-miR-99b-5p suggests that this family of miRNAs particularly enriched in the brain might be involved in different aspects of the disorder.

Our findings did not validate previously identified changes in circulating EVs, such as hsa-miR-486-5p and hsa-miR-1246 [13] or hsa-miR-379-3p, hsa-miR-1976, and hsa-miR-151a-5p [49], suggesting that plasma EVs may reflect alterations complementary to those occurring in the brain. Additionally, our study did not detect alterations in hsa-miR-137, a sRNA consistently associated to schizophrenia in GWAS studies that is altered in the prefrontal cortex [50, 51].

Taken together, our results, along with previous reports, suggest that sRNA profiles are unique across different tissues and brain regions, potentially playing complementary and distinct roles in the pathophysiology of the disorder. Remarkably, all the aforementioned species could play, at least in part, a substantial role in the spatial memory alterations observed in mice transduced with sRNAs from patients with schizophrenia.

In the present work we observed that isolated sRNAs from human hippocampi of patients with schizophrenia induced highly specific changes in some mouse declarative memory skills such as spatial memory, a cognitive ability described to be impaired in schizophrenia [52, 53], but not in recognition memory which is also widely reported to be altered in schizophrenia [54]. Interestingly, these variations were not accompanied by changes in other aspects of the mouse behavioural repertoire such as exploratory activity, anxiety-like behaviour, body weight changes, and others. Altogether, our results highlight the specificity of the impact of dysregulated hippocampal sRNAs in the regulation of spatial memory deficits. Given the reported roles of various schizophrenia-dysregulated sRNAs, the observed effects on specific cognitive domains are likely due to the combined action of multiple dysregulated sRNAs. Future studies using oligonucleotides to block the effects of individual sRNAs should help identify the main contributors.

**Fig. 7:**
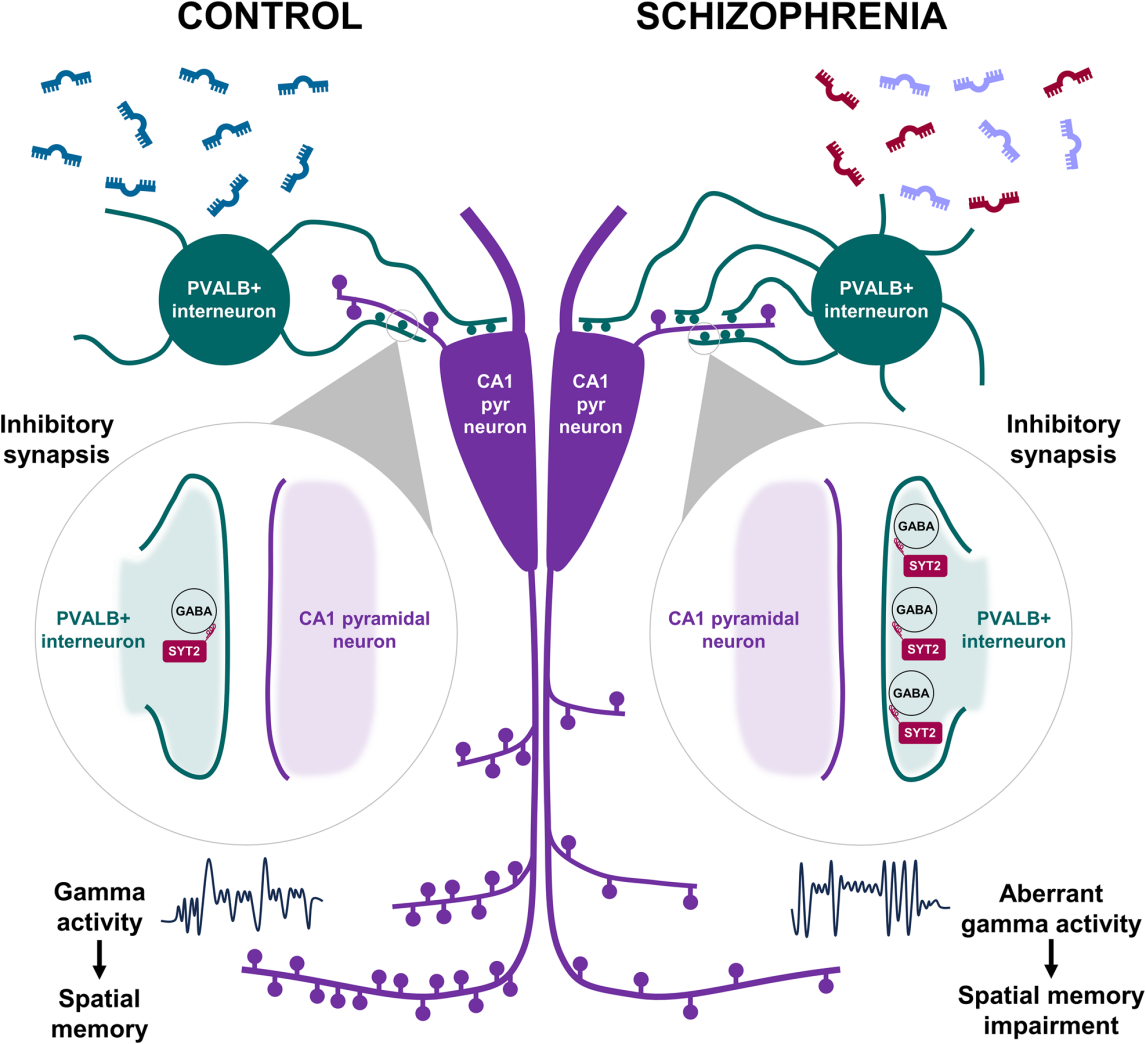
Schematic representation of potential molecular mechanisms underlying spatial memory impairment in schizophrenia due to dysregulated hippocampal small RNAs. In control individuals (left), healthy sRNA transcriptome allows the correct neurotransmission between parvalbumin-positive interneurons (dark green) and excitatory CA1 pyramidal neurons (purple) shown by the normal expression levels of SYT2 (magenta) at the inhibitory terminals of the interneurons. This excitatory/inhibitory balance mediates generation of gamma-frequency oscillations and proper spatial memory encoding and retrieval at a physiological level. In schizophrenia (right), aberrant sRNA transcriptome impairs synaptic transmission at the presynaptic buttons of inhibitory interneurons, generating an accumulation of synaptic vesicles loaded with inhibitory neurotransmitters (e.g., GABA) as shown by the higher number of SYT2-positive clusters. These deficits in inhibitory synaptic transmission between parvalbumin-positive interneurons and CA1 pyramidal cells lead to aberrant gamma activity and spatial memory impairment. PVALB parvalbumin, Pyr pyramidal.

Evaluating the impact of human sRNAs transduced into the mouse hippocampus, we found unanticipated changes in novel genes that might be relevant for the pathophysiology of the disease. Although we observed slight differences in the effects of Pool 1 and Pool 2 schizophrenia sRNAs on gene expression, reflecting the intrinsic biological diversity of the patients from whom the sRNAs were derived, we found that the protein synaptotagmin 2 (SYT2) is a potential direct or indirect molecular target of dysregulated sRNAs from these patients. *SYT2* is a relatively underexplored gene that is enriched in the brain [55], specifically in the presynaptic terminals of parvalbumin interneurons in both the cortex and hippocampus where it acts as a calcium sensor for fast release of neurotransmitters [27]. While there is some evidence regarding synaptotagmin 1 (SYT1) and its role in clathrin-mediated endocytosis in schizophrenia [56], no associations have been made to date between SYT2 and schizophrenia. Of note, SYT2 shares a high homology in terms of biochemical characteristics and functions with SYT1 [55]. Moreover, synaptotagmin 11 (SYT11), another member of the synaptotagmin family, has been identified as a potential genetic risk factor for schizophrenia [57]. Deficiency of SYT11 leads to excessive dopamine release in the striatum and PFC, which would contribute to positive and negative symptoms of schizophrenia, respectively [57]. SYT2 is involved in Ca^2+^-dependent neurotransmitter release in striatal neurons [55]. In fact, genetic deletion of *Syt2* has been shown to cause a significant increase in spontaneous neurotransmitter release [30]. Interestingly, we observed an increase in SYT2 levels in hippocampal parvalbumin interneurons following hippocampal transduction with sRNAs from patients and we showed that this increase in SYT2-positive clusters is also present in the hippocampus of these patients. This raises the possibility that synaptic vesicles loaded with inhibitory neurotransmitters (e.g., GABA) might be accumulating at the presynaptic boutons of these interneurons, reducing its release to regulate CA1 pyramidal neurons and contributing to their disinhibition and aberrant hyperactivity in the hippocampus. Supporting these ideas, parvalbumin interneurons have been extensively studied in the context of schizophrenia, where they are known to be impaired in generating and maintaining gamma-frequency cortical rhythms [58]. Gamma-frequency oscillations are crucial for complex cognitive functions, and in the dorsal hippocampus, they are key for spatial memory encoding and retrieval [59, 60]. Thus, we propose that alterations in hippocampal SYT2 levels in mice injected with sRNAs from schizophrenia patients reproduce some of the molecular phenotypes of the disease, impairing neural synchronization and/or the generation of gamma-frequency rhythms in mice and thereby hindering proper spatial memory acquisition (Fig. 7).

We also observed a few alterations in microglial but not astroglial phenotypes in the hippocampus of mice injected with sRNAs from affected individuals. This aligns with the current understanding that schizophrenia is strongly associated with complex alterations in the immune system, both peripherally [61] and within the brain [62]. Indeed, sRNAs may play a role in these immune system changes [63]. In this context, sRNAs from the hippocampi of patients induced changes in microglial morphology, making them more elongated (increased aspect ratio) and polarized (less round). This altered profile may be linked to specific microglial phenotypes, such as aberrant and increased synaptic engulfment [64]. While we did not directly demonstrate this, such microglia-initiated synaptic engulfment could lead to the loss of spine density in CA1 pyramidal neurons—a mechanism already proposed in schizophrenia [65]. Additionally, such changes could be concomitant to the increased number of SYT2-positive presynaptic boutons on parvalbumin-positive interneurons. Future research should deepen these possibilities and the underlying molecular mechanisms.

Notwithstanding the relevance of our findings, our study has some limitations. Although our sample size is larger than previous studies using *post-mortem* tissue, we primarily used samples from male patients with schizophrenia and we could only include both male and female samples in the Control pool. Given the importance of gender-specific gene expression patterns that may influence the development and prevalence of different neuropsychiatric disorders [66], our approach could have introduced some variability and bias into our molecular analyses. Future experiments using sex-matched samples could help clarify sex differences in sRNA-regulated gene expression networks that may impact clinical manifestations of schizophrenia [67]. In fact, since men usually exhibit a more severe form of schizophrenia and poorer treatment response and outcomes, their cognitive performance might be expected to be more impaired than in women. This could be related to differences in their sRNA profiles that could be assessed with our approach. However, it is also important to note that evidence underlying these sex differences in the symptomatology of the disease remains inconclusive and inconsistent, with studies pointing in different directions [68, 69].

Moreover, while differences in antipsychotic exposure across patients may contributed to variability in hippocampal sRNA composition, the observation that two independently derived sRNA pools produced comparable cognitive impairments indicates that the sRNA effects are strong and convergent enough to generate similar phenotypic consequences.

Additionally, quality controls such as RIN values in some of the *post-mortem* samples were only moderately good. In terms of key sRNA candidates, our study did not identify a single, clear sRNA that plays a major role in the observed phenotypes which goes in line with the complexity of the underlying pathogenic mechanisms. Instead, we propose that a combination of several sRNAs may be required to induce the specific spatial memory deficits identified in our study.

In conclusion, this is the first demonstration that human sRNAs from schizophrenic brains are sufficient to influence higher-order cognitive functions, such as spatial memory, in the context of schizophrenia. Future studies should further investigate the role of these dysregulated sRNAs, considering both the diversity of sRNA profiles across different tissues and their specific roles in the disorder.

## Materials and methods

### Human small RNA isolation and preparation of small RNA pools

The use of human hippocampal frozen and fixed samples was approved by the local Ethical Committee of the University of Barcelona (Institutional Review Board IRB00003099. CER122306). Patient ID, age, sex, pathological diagnosis, *post-mortem* delay (PMD), Positive and Negative Syndrome Scale (PANSS), and treatment information are provided in Supplementary Table 1. Both the fixed and frozen tissues came from the same patients and from the same biobank.

For the hippocampal sRNA extraction and quality control, a total of 9 control and 20 schizophrenia samples were homogenized as described in [26]. Briefly, brain tissue was placed in QIAzol solution (QIAGEN, 79306) and lysed using a polytron, followed by RNA extraction using the miRNeasy mini kit (QIAGEN, 217004) according to the manufacturer’s instructions. All samples were eluted in 40 µL of RNAse-free water and total RNA quantity and quality were measured using a ND-1000 spectrophotometer (Thermo Fisher Scientific) and a TapeStation 4200 (Agilent), respectively. Most of the samples used for the experiments had an RNA integrity number (RIN) of 6 or higher. sRNA fractions were purified from total RNA using the RNA Clean & Concentrator^TM^-5 kit (Zymo Research; R1015).

Pools of sRNA samples were prepared using a total of 4.7 µg of sRNAs from each patient to reach a final concentration of 0.24 µg/µL. The 9 control patients were mixed into a Control Pool; whereas the 20 patients with schizophrenia were distributed into a Pool 1 and Pool 2 of sRNAs of 10 patients each to have two comparable groups in terms of symptomatology disease severity (total PANSS mean Pool 1 = 104.7 ± 29.01; total PANSS mean Pool 2 = 108.3 ± 40.78; Two-tailed t-test, *P* = 0.8612) and RNA quality (RIN mean Pool 1 = 6.49 ± 0.80; RIN mean Pool 2 = 6.55 ± 0.73; Two-tailed t-test, *P* = 0.8622) (Supplementary Table 2).

### Small RNA sequencing of human hippocampal samples

Sequencing libraries were prepared from hippocampal purified sRNAs in a representative subset of control individuals (N = 6) and schizophrenia patients (*N* = 12, 6 patients from each pool) using the NEBNext Small RNA Library Prep Set for Illumina (New England Biolabs), according to the manufacturer’s instructions. Individual libraries were subjected to quality analysis using a Bioanalyser 2100 (Agilent Technologies Inc.), and size selection (140–165 bp) was performed in 6% polyacrylamide gel, which corresponded to adapter-ligated constructs derived from the 21 and 30 nt RNA fragments, respectively. Indexed libraries were equimolarly pooled and sequenced in an HiSeq2500 (Illumina) and 50 nt single-end reads were produced.

### Small RNA sequencing data processing and analysis

The quality of the sequenced fastq files was checked using FastQC software (v0.12.1) (http://www.bioinformatics.babraham.ac.uk/projects/fastqc/). Adapter trimming was performed with Cutadapt [70] and the trimmed reads were aligned to the human genome (version Ensemble hg 19) using the STAR aligner (v2.7.9a) [71]. Quantification and annotation were performed with the SeqCluster [15] and ExceRpt [16] tools. To have a complete view of the sRNA transcriptome, including ribosomal RNA (rRNA) fragments and tRNA-derived fragments (tRFs) we used SeqCluster tool. SeqCluster uses a heuristic iterative algorithm to deal with multi-mapped events. Sequences are consistently and non-redundantly grouped if they map onto the same genomic site defining ‘clusters’ of co-expressed sRNAs. Clusters are then classified according to the type of precursor where sRNAs map, including rRNA-and tRF-clusters. On the other hand, during the pre-processing step, ExceRpt filters out highly abundant sRNAs mapping onto rRNAs and makes an oversimplification of the highly complex tRFs landscape by only considering the type of isoacceptor. We generated ExceRpt and SeqCluster count matrices with raw reads of each sRNA biotype in every sample. To detect outlier samples in the RNAseq analysis, we generated principal component analysis (PCA) plots with the normalized sRNAs sequence count matrix and we did not identify any outlier.

For downstream analyses, we considered RNAs with 10 or more reads in at least half of the samples. The R prcomp() function (R v4.3.2) was used for non-supervised multivariate analysis [72] and R packages tidyverse (v2.0.0) and ggfortify (v0.4.16) were used for multivariate analysis and generation of the PCA plots. Differential expression (DE) of the sRNAs between control and schizophrenia samples was performed using the negative binomial linear models implemented within the DESEq2 R/Bioconductor (v4.0.0) package [73]. Code is available from the corresponding authors upon reasonable request. Sequencing data has been deposited in NCBI’s Gene Expression Omnibus and are accessible through GEO Series accession number (GSE325449).

To discover further possible implications of DE sRNAs in patients with schizophrenia and explore the functional roles of some of those DE miRNAs we used EnrichR (https://maayanlab.cloud/Enrichr/) [74], where we introduced the list of target genes available on the mirTarBase curated miRNA–target interactions database (https://mirtarbase.cuhk.edu.cn/~miRTarBase/miRTarBase_2022/php/index.php) [17]. Functional annotations for modules of interest were generated using the web server SynGO (https://www.syngoportal.org/) [18].

### Animals

All mice used in the experiments were 3 to 4-months-old wild-type males. The animals were housed with access to food and water *ad libitum* in a colony room kept at 19–22 °C and 40–60% humidity, under an inverted 12:12 h light/dark cycle (from 08:00 to 20:00). For all the experiments, C57BL/6JOlaHsd mice from Envigo were used. All animal procedures were approved by local and national committees [Universitat de Barcelona, CEEA (315/18) and Generalitat de Catalunya (DAAM 10141)], in accordance with the European Communities Council Directive (86/609/EU).

### Mouse stereotaxic surgeries and hippocampal infusion of human small RNA

Animals (*N* = 10 per experimental group) were stereotaxically injected with one of the following pooled sRNAs isolated from human *post-mortem* hippocampi: mice injected with sRNAs pooled from 9 control patients (Pool Control), mice injected with sRNAs pooled from 10 patients with schizophrenia (Pool 1) and mice injected with sRNAs pooled from a second cohort of other 10 patients with schizophrenia (Pool 2). Artificial cerebrospinal fluid (Tocris Bioscience, 3525) was used as a vehicle control in a fourth group of mice (Vehicle).

To implant the cannulas, mice were anaesthetized with isoflurane (2% induction, 1.5% maintenance) and 2% oxygen, and placed into a stereotaxic apparatus in a flat skull position. Body temperature was maintained constant during the intervention using a heating pad. Prior to surgery, a topical anaesthetic (EMLA) was applied to the surgical site. Stainless steel bilateral cannulas (26-gauge, Bilaney Consultants GmbH) were implanted in the CA1 of the dorsal hippocampus. Cannulas consisted in a horizontal plate containing two guide cannulas in which either dummy cannulas (without projection) or infusion cannulas (0.4 mm projection) could be introduced to reach the desired coordinates from the bregma (millimetres): anteroposterior, –2.0; mediolateral, ±1.5; and dorsoventral, −1.4. After surgery, all mice received a subcutaneous injection of meloxicam (Metacam, 0.2 mL of 2 mg/mL) for postoperative analgesia and were kept in a warm place until recovered from anaesthesia. One week after the surgery, two bilateral infusions of 2 µL of vehicle or sRNAs each at [0.24 µg/µL] separated by a time lapse of 48 hours between both injections were performed at 0.25 µL/min using an infusion pump in freely moving mice, following a similar experimental design as described elsewhere [26], which allowed the detection of both molecular and behavioural alterations. To ensure complete diffusion and avoid reflux, infusion cannulas were left in place for 5 minutes after the injection. Animals were subjected to different behavioural tasks 24 hours after the last infusion, and they were sacrificed by cervical dislocation 24 hours (for RNA extraction/immunofluorescence) or 5 days (for Golgi staining) after the last behavioural test.

### Behavioral tests

#### Novel object recognition test (NORT)

To study recognition memory, we used the NORT. Briefly, a grey square open-top arena of 40 × 40 × 40 cm (length, width and height, respectively) was used. Mice were first habituated to the arena (1 day, 20 min), also as an open field test. On day 2, two identical objects were placed on one side of the arena and mice were allowed to explore them for 10 min. Exploration was considered when the mouse was in contact with the object and sniffed it. 24 h later (day 3), one object was replaced for a new one and mice were allowed to explore the arena and the objects for 10 min. At the end of each trial, defecations were removed, and both the arena and the objects were cleaned with 30% ethanol. Animals were tracked and recorded with SMART junior v3.0 software (Panlab). Time exploring the new vs old object (%), number of nose pokes on the new vs old object (%), and Discrimination Index were analyzed. The object preference was determined by calculating the time spent (or number of nose pokes) in each object × 100/ total time spent in both objects (old and new); whereas Discrimination Index was calculated as (time exploring the new object – time exploring the old object) / total time spent in both objects (old and new).

#### T-maze test

To evaluate spatial short-term memory, we used a T-maze. Briefly, the apparatus used was a maze consisting of three arms, two of them situated at 180° from each other, and the third, representing the stem arm of the T, situated at 90° with respect to the other two. All arms were 45 cm long, 8 cm wide and enclosed by a 20 cm wall. Light intensity was 10-15 lux throughout the maze. Two identical guillotine doors were placed in the entry of the arms situated at 180°. The maze was elevated 60 cm above the floor. In the training trial, one arm was closed (novel arm) and mice were placed in the stem arm of the T (home arm) and allowed to explore this arm and the other available arm (familiar arm) for 10 min, after which they were returned to the home cage. After an inter-trial interval of 1 hour, mice were placed in the stem arm of the T-maze and allowed to freely explore all three arms for 5 min. At the end of each trial, defecations were removed, the apparatus was cleaned with 30% ethanol. Animals were tracked and recorded with Smart junior 3.0 software (Panlab). Distance, number of entries and time spent in the novel vs familiar arm (%) were analyzed. The arm preference was determined by calculating the time (or entries or distance) spent in each arm × 100/time (or entries or distance) spent in both arms (familiar and novel).

### Total RNA extraction from mouse hippocampal samples

For the hippocampal RNA extraction and quality control, mouse samples were homogenized in QIAzol solution (QIAGEN, 79306) and lysed using a polytron, followed by RNA extraction using the miRNeasy mini kit (QIAGEN, 217004) according to the manufacturer’s instructions. All samples were eluted in 40 µL of RNAse-free water and total RNA quantity and purity were measured using a ND-1000 spectrophotometer (Thermo Fisher Scientific). RNA integrity was assessed using a 2100 Bioanalyser (Agilent Technologies Inc.), according to manufacturers’ protocols. The average RIN value for our mouse samples was 8.5.

### RNA sequencing of mouse hippocampal samples

Libraries were prepared using the TruSeq Stranded mRNA Sample Prep kit v2 (RS-122-2101/2) according to the manufacturer’s protocol. Briefly, 500 ng of total RNA were used for poly(A)-mRNA selection using streptavidin-coated magnetic beads and were subsequently fragmented to ∼300 bp. cDNA was synthesized using reverse transcriptase (SuperScript II, Invitrogen, 18064-014) and random primers. The second strand of the cDNA incorporated dUTP in place of dTTP. Double-stranded DNA was further used for library preparation. dsDNA was subjected to A-tailing and ligation of the barcoded Truseq adapters. Library amplification was performed by PCR using the primer cocktail supplied in the kit. All purification steps were performed using AMPure XP beads. Final libraries were analysed using Fragment Analyser to estimate the quantity and check size distribution and were then quantified by qPCR using the KAPA Library Quantification kit (KK4835, KapaBiosystems) before amplification with Illumina’s cBot. Sequencing was performed using the HiSeq2500 equipment (Illumina), and 50 nt single-end reads were generated, using the v4 chemistry.

### RNA sequencing data processing and analysis

The quality of the sequencing data was checked using the FastQC software (v0.11.5). FastQC: a quality control tool for high throughput sequence data (available online at http://www.bioinformatics.babraham.ac.uk/projects/fastqc). An estimation of ribosomal RNA in the raw data was obtained using riboPicker (v0.4.3). Reads were aligned to the GENCODE version of the Mus musculus genome, release M20 (GRMm38/mm10 assembly) using the STAR mapper (v2.5.3a). The raw read counts per gene was also obtained using STAR (–quantMode TranscriptomeSAM GeneCounts option) and the GENCODE release M20 annotation (ftp://ftp.ebi.ac.uk/pub/databases/gencode/Gencode_mouse/release_M20/gencode.vM20.annotation.gtf.gz) [75]. The R/Bioconductor package DESeq2 (v1.22.2) [73] (R v3.5.0) was used to assess the differentially expressed genes (DEGs) between experimental groups, using the Wald statistical test and the False Discovery Rate for the p-value correction. Before the differential expression analysis, genes with the sum of raw counts across all samples below 10 were discarded, the library sizes were normalized using the default DeSeq2 method, and the read counts were log2 transformed. To exclude false positive genes, genes with low expression levels (baseMean < 10) were excluded from the list of DEGs. Sequencing data has been deposited in NCBI’s Gene Expression Omnibus and are accessible through GEO Series accession number (GSE325450).

For the gene functional enrichment analysis and to discover further functional implications of those DEGs we used EnrichR (https://maayanlab.cloud/Enrichr/) [74]. Functional annotations for modules of interest were generated using the web server SynGO (https://www.syngoportal.org/) [18].

### Golgi staining and quantification

Animals were sacrificed, and half of the brain was obtained and kept in Golgi-Cox solution for 3 weeks. Then, 200 μm slices were obtained using a vibratome (Leica, VT 1000S) and processed as previously described [76]. Secondary dendrites from CA1 pyramidal neurons were photographed using a Widefield AF6000 Monochroma Camera Leica microscope. A maximum of 3 dendrites from the same neuron were taken. Z-stacks from 0.2 μm were obtained in a bright field and 63×/1.40 numerical aperture oil objective. Dendritic segments (average 40 μm) were traced with FIJI/ImageJ software, and spine density was quantified using the cell counter plugin. Around 84–113 dendrites were analyzed per condition. From each condition, 5 mice were quantified. Image analysis was performed blindly.

### Immunofluorescence

Mice were sacrificed and half of the brain was dissected out and maintained in 4% paraformaldehyde (PFA) 0.1 M for 5 days in agitation at 4 °C. After fixation, free-floating coronal sections (30 μm) were obtained using a vibratome (Leica, VT 1000S). Immunofluorescence was performed as previously described [34]. Briefly, brain sections were washed and incubated for 30 min in 50 mM NH_4_Cl followed by blocking and permeabilization for 1 hour in PBS-Triton X-100 with 0.02% sodium azide, 3% normal donkey serum (Jackson ImmunoResearch Europe Ltd., 017-000-121) and 0.2% bovine serum albumin (Sigma, A9647). For human tissue, an initial step for antigen retrieval incubating for 30 min at 80 °C with 10 mM Trisodium Citrate with 0.05% Tween-20 was included. Slices were incubated overnight by shaking at 4 °C with the following primary antibodies: Synaptotagmin 2 (1:500, Abcam, #ab154035), IBA1 (1:500, Abcam, #ab5076), GFAP (1:500, Dako, #Z0334) and Parvalbumin (1:1250, Swant, #PV27a). Sections were then incubated for 2 hours at room temperature with the specific fluorescent secondary AlexaFluor-488 anti-rabbit (Jackson Immunoresearch, #711-545-152) and Cy3 AffiniPure anti-goat and anti-mouse (Jackson Immunoresearch, #705-165-003 and #715-165-150 respectively). All secondary antibodies were diluted 1:200 in blocking solution. Finally, nuclei were stained for 10 min with 4′,6-diamidino-2- phenylindole (DAPI, Sigma-Aldrich, #D9542). No signal was detected in control sections incubated in the absence of the primary antibody.

### Image acquisition and analysis

Immunostained tissue sections (30-μm thick) containing the dorsal hippocampus were imaged using a Leica Confocal SP5-II (40×/1.25 numerical aperture or 63×/1.40 numerical aperture oil objectives, 5×digital zoom, 1-Airy unit pinhole). At least two slices per mouse were analyzed, and up to two representative hippocampal images were obtained from each slice. Seven frames were averaged per z-step throughout the study. Confocal z-stacks were taken at 1024×1024-pixel resolution every 2 μm. For human tissues, one slice per patient and at least two representative images per slice were analyzed. Estimation of the number of SYT2-positive puncta were quantified as previously described [77]. Quantification of shape parameters and mean grey value (mean intensity) in IBA1 and GFAP staining were determined as described elsewhere [34].

### Statistics

Results obtained from the behavior, Golgi staining and immunofluorescence were analyzed using GraphPad Prism software version 10.0 (License ID75521). Data were represented as mean ± standard error of the mean (SEM). Sample sizes were determined by using power calculations: 0.05 alpha value, 1 estimated sigma value, and 75% of power detection. All mice bred for the experiments were used for pre-planned experiments and randomized to experimental groups. Outliers were identified with the ROUT method. Data distribution was analyzed by applying the Shapiro-Wilk normality test. As indicated in the figure legends, for multi-component variables comparison one-way ANOVA followed by Tukey’s or Dunnet’s *post hoc* tests, and two-way ANOVA followed by Bonferroni’s *post hoc* test were employed. A 95% confidence interval was used and a *p*-value < 0.05 was considered significant. The number of samples (N) and p-values are specified in each figure legend.

## Supplementary information

Supplementary figures and tables

## Data Availability

Sequencing data has been deposited in NCBI’s Gene Expression Omnibus and are accessible through GEO Series accession number (GSE325449). Rest of data and materials will be made available upon reasonable request.
