# Supplementary material for: Hippocampal small RNAs from patients with schizophrenia induce specific cognitive and neural phenotypes in mice
Source: Cell Death Discov. 2026 May 25;12:315. doi: 10.1038/s41420-026-03166-z (PMC13385819; doi:10.1038/s41420-026-03166-z)
Supplement: Supplementary file 1 — Supplementary figures and tables [file 41420_2026_3166_MOESM1_ESM.docx]

**
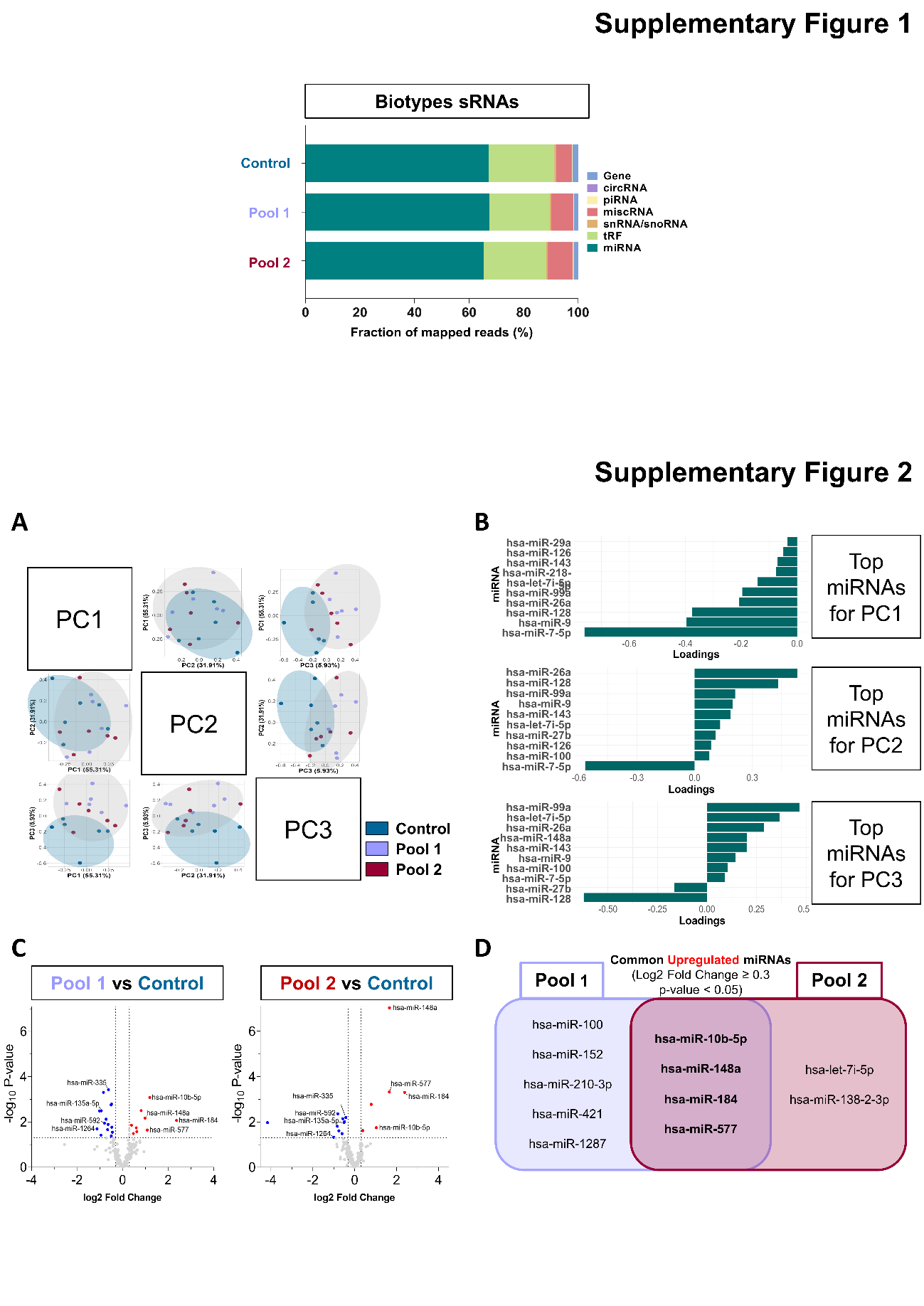
**

**Supplementary Figure 1: Characterization of small RNAs from *post-mortem* hippocampi of patients with schizophrenia and unaffected controls using ExceRpt annotation tool.**

sRNAs’ biotypes using ExceRpt tool are shown. Fraction of mapped reads in each sRNA pool (Control, Pool 1 and Pool 2) annotating onto the different sRNA biotypes identified with ExceRpt (N = 6 patients/pool). miRNA = microRNAs. tRF = tRNA-derived fragments. snRNA/snoRNA = small nuclear/nucleolar RNAs. miscRNA = miscellaneous RNA. piRNA = Piwi-interacting RNA. circRNA = circular RNA. Gene = gene fragments.


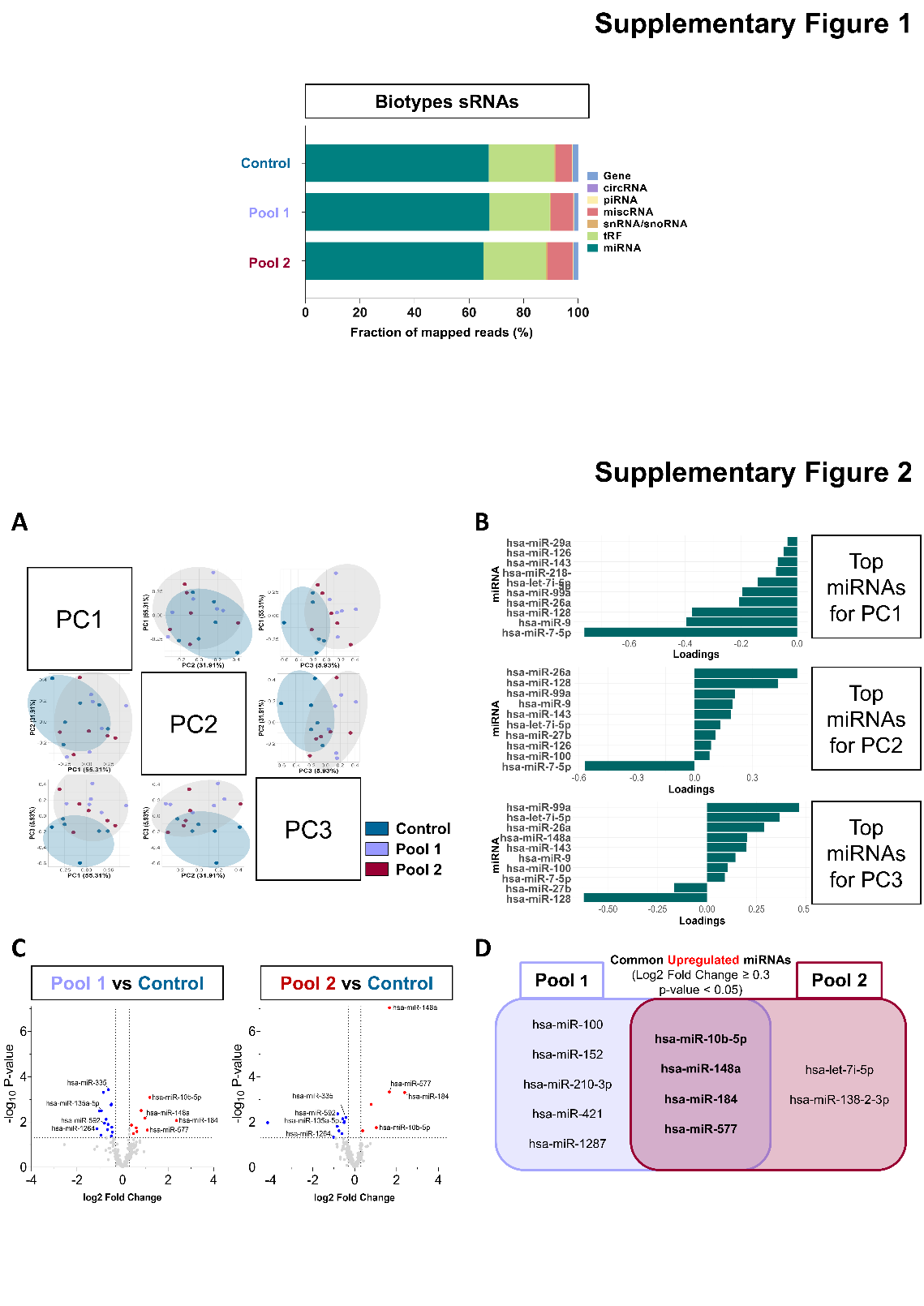


**Supplementary Figure 2: Characterization of microRNAs from *post-mortem* hippocampi of patients with schizophrenia and unaffected controls using SeqCluster annotation tool.**

miRNA PCA plots (**a**). PCA plots constructed with the matrix of counts of only miRNAs obtained using SeqCluster tool and combining the first three components that account for more than 90% of the variance explained (N = 6 patients/pool). miRNA PC loadings (**b**). PC loadings of the top 10 miRNAs species contributing to the separation of control and schizophrenia samples in each of the three components. DE miRNAs in the hippocampus of schizophrenia compared to control individuals (**c**). Volcano plots constructed with the DE miRNAs of Pool 1 and Pool 2 vs Control using the miRNA matrix of counts obtained with SeqCluster. Red dots highlight the significantly upregulated miRNAs (Log2 Fold Change ≥ 0.3, p-value < 0.05) and blue dots illustrate the downregulated miRNAs (Log2 Fold Change ≤ -0.3, p-value < 0.05). Dots representing illustrative miRNA species are labelled. Venn diagram showing the overlap between significantly upregulated miRNAs detected in both pools of patients with schizophrenia (**d**). Screening threshold used was Log2 Fold Change ≥ 0.3 and p-value < 0.05 for both Pool 1 and Pool 2 groups of patients.

**
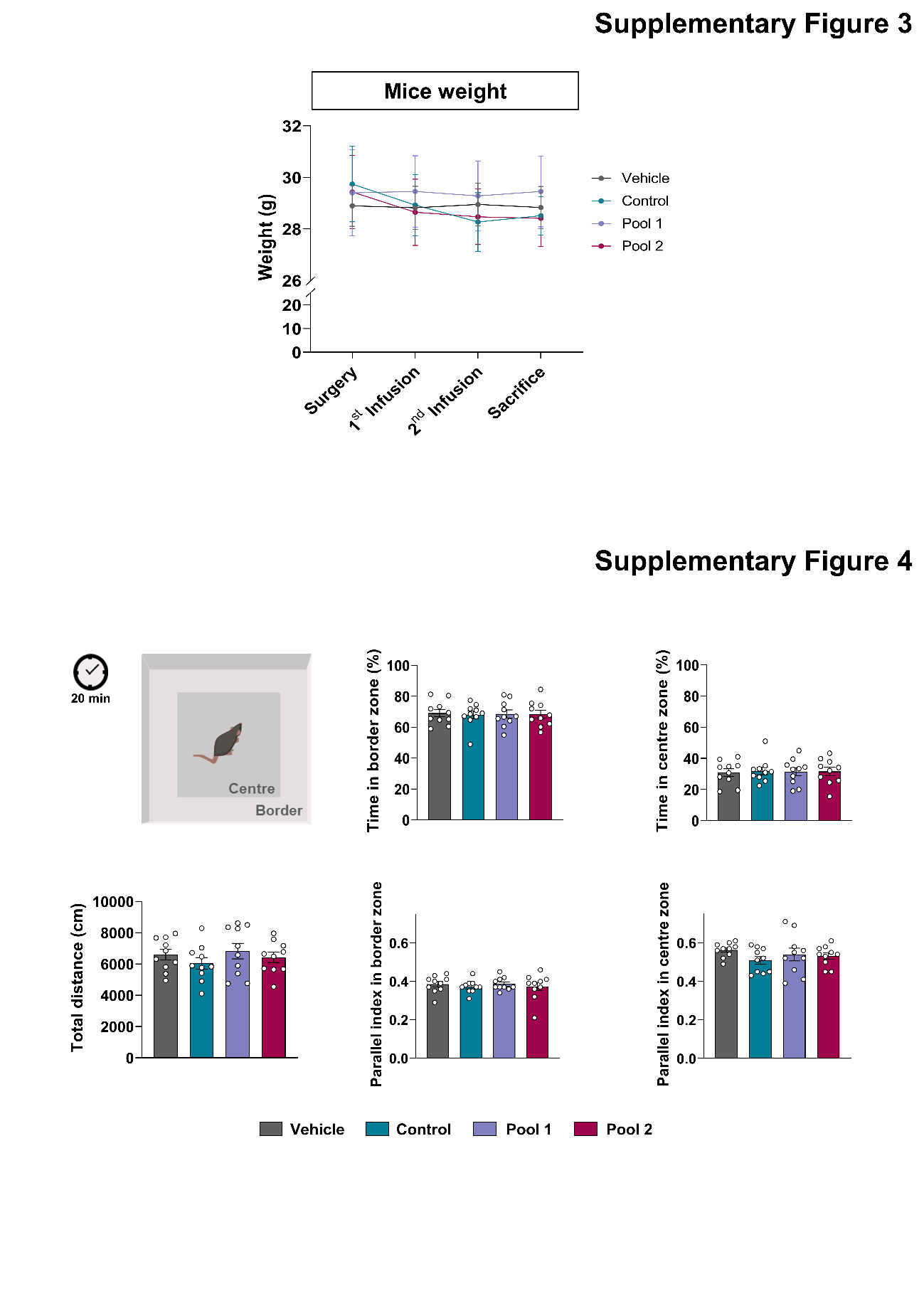
**

**Supplementary Figure 3: Effects of the injection of small RNAs on mice body weight.**

Dot plot with connecting line shows no significant changes in the mean body weight of mice between the different experimental groups (Vehicle, Control, Pool 1 and Pool 2) during the experimental procedure (at surgery, first infusion of sRNAs, second infusion of sRNAs and day of sacrifice) (two-way ANOVA, group effect F(_3,27_)=0.06349, P=0.9787; interaction effect F(_9,81_)=1.159, P=0.3327). Bonferroni’s *post hoc* test was performed, and values are mean ± SEM. N = 7-9 mice/group.


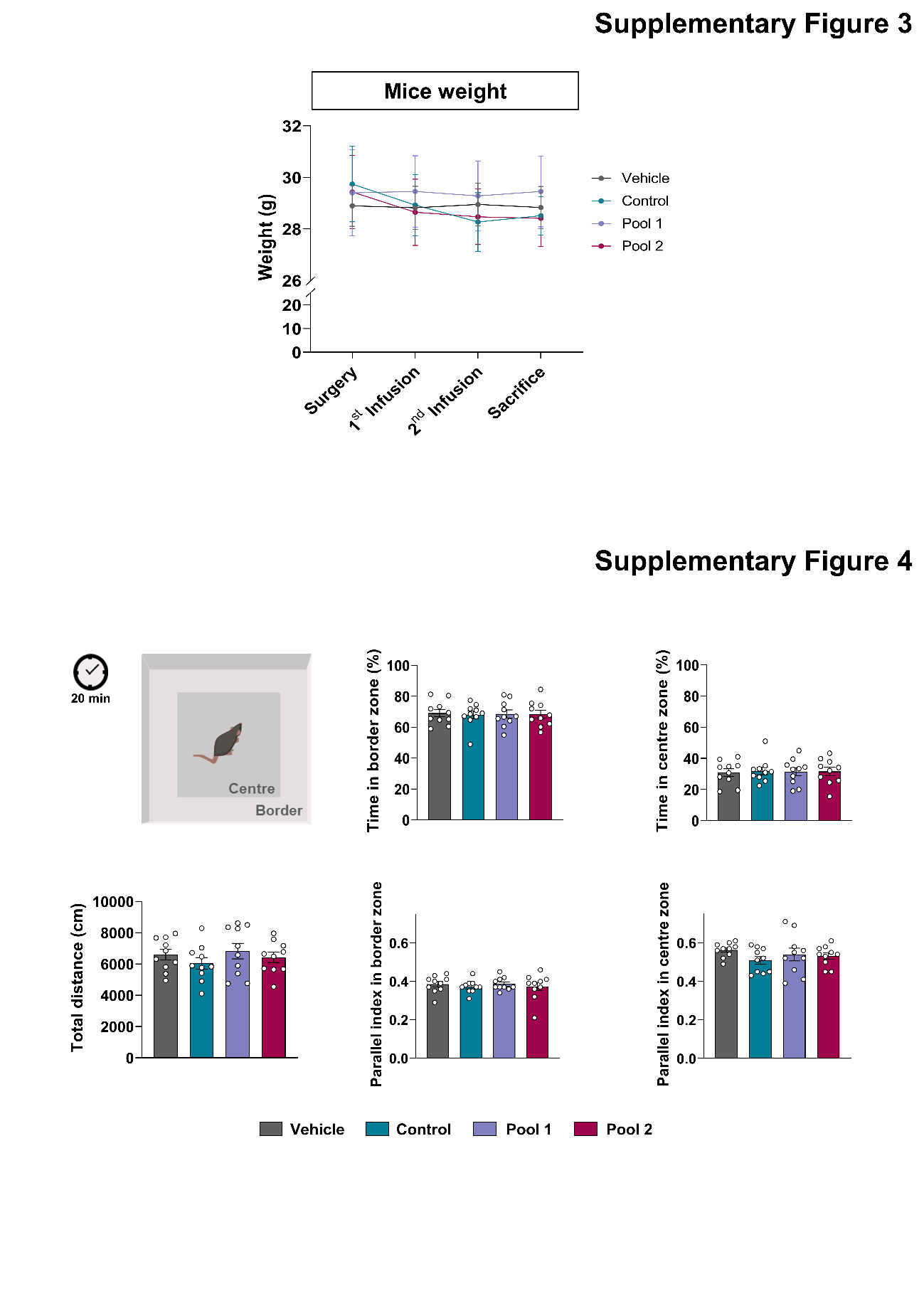


**Supplementary Figure 4: Behavioral effects on the open field test after intra-hippocampal injection of small RNAs from *post-mortem* hippocampi of patients with schizophrenia and unaffected controls.**

Results from the open field test to assess anxiety-like levels as well as locomotor activity and exploration in mice. Two parameters measuring anxiety-like levels were analysed: percentage of time in border zone (one-way ANOVA, F(_3,36_)=0.03375, P=0.9915) and percentage of time in centre zone (one-way ANOVA, F(_3,36_)=0.03375, P=0.9915). Three parameters assessing locomotor activity and navigation of the mice were analysed: total distance (one-way ANOVA, F(_3,36_)=0.7305, P=0.5406), parallel index in border zone (one-way ANOVA, F(_3,36_)=0.2561, P=0.8565) and parallel index in centre zone (one-way ANOVA, F(_3,36_)=1.032, P=0.3898). Tukey’s *post hoc* test was performed and values are mean ± SEM. N = 10 mice/group.

**
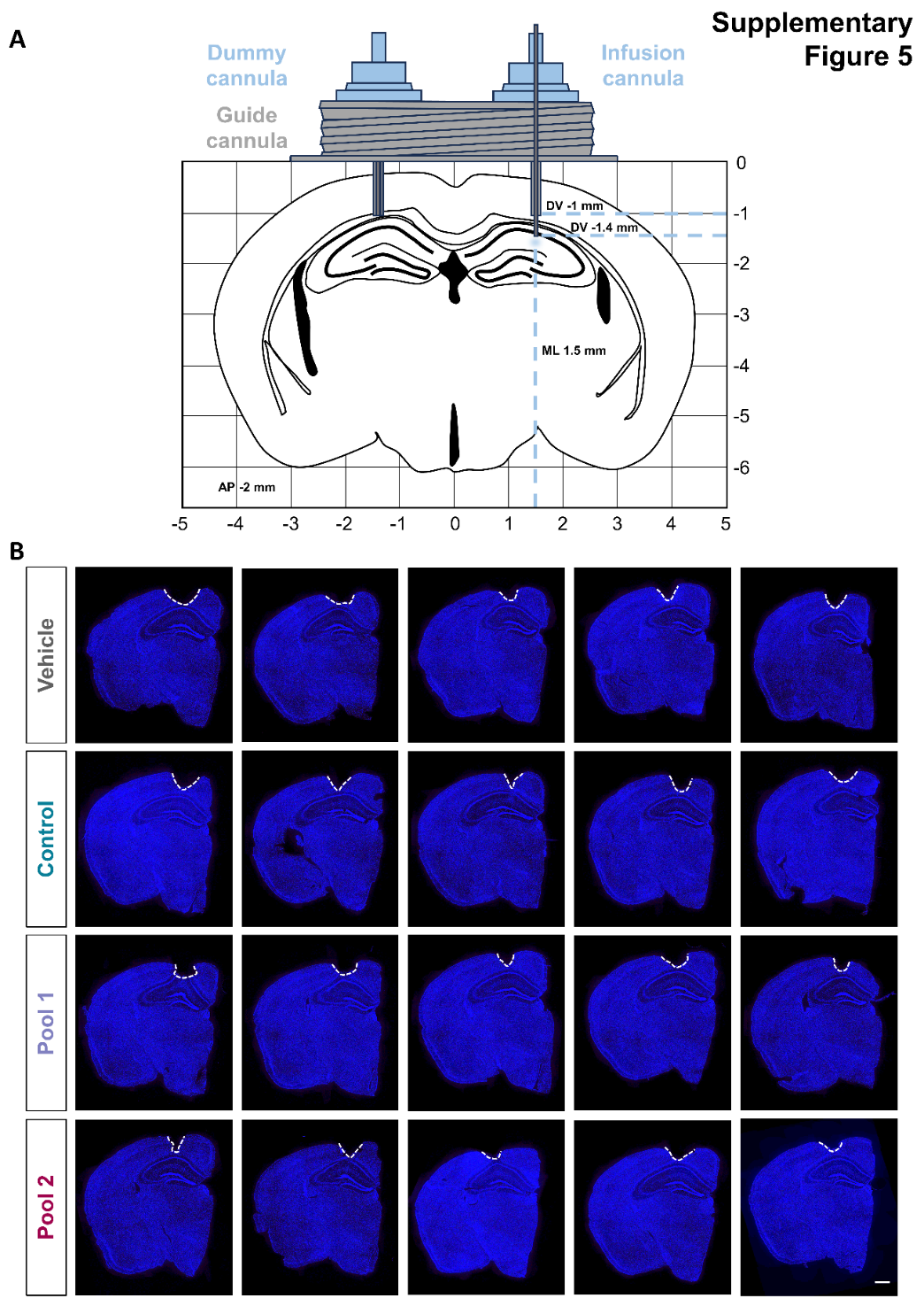
**

**Supplementary Figure 5: Intra-hippocampal injection of small RNAs using cannulas located at the CA1 region.**

Schematic representation of a hippocampal bilateral cannula implanted in the mouse brain (**a**). Specific coordinates of implantation of the guide cannula into CA1 hippocampal region with a dummy cannula (left) and an infusion cannula (right) are detailed. AP = anteroposterior. DV = dorsoventral. ML = mediolateral. Representative hippocampal sections stained with DAPI are depicted (N = 5 mice/group) (**b**). White dotted lines show correct implantation of the cannulas for sRNAs injection at the desired CA1 coordinates. Scale bar: 500 microns.

**Supplementary Table 1. Human *post-mortem* hippocampal samples**. For each subject, information details about age, sex, pathological diagnosis, *post-mortem* delay (PMD), Positive and Negative Syndrome Scale (PANSS) and treatment information are provided.

| ID | Age | Sex | Pathological diagnosis | PMD (hh:mm) | PANSS + | PANSS - | PANSS General | Antipsychotic treatment |
| --- | --- | --- | --- | --- | --- | --- | --- | --- |
| 1557 | 86 | M | Control | 7:25 | n.a. | n.a. | n.a. | n.a. |
| 1679 | 90 | F | Control | 13:40 | n.a. | n.a. | n.a. | n.a. |
| 1818 | 78 | M | Control | 5:00 | n.a. | n.a. | n.a. | n.a. |
| 1858 | 83 | F | Control | 7:30 | n.a. | n.a. | n.a. | n.a. |
| 1870 | 97 | F | Control | 7:20 | n.a. | n.a. | n.a. | n.a. |
| 1888 | 93 | F | Control | 5:30 | n.a. | n.a. | n.a. | n.a. |
| 1937 | 83 | F | Control | 7:33 | n.a. | n.a. | n.a. | n.a. |
| 1949 | 86 | M | Control | 7:35 | n.a. | n.a. | n.a. | n.a. |
| 2193 | 89 | F | Control | 8:50 | n.a. | n.a. | n.a. | n.a. |
| 001 | 80 | M | Schizophrenia | 2:00 | -- | -- | -- | No treatment |
| 026 | 77 | M | Schizophrenia | 5:30 | -- | -- | -- | Olanzapine |
| 027 | 84 | M | Schizophrenia | 2:20 | 24 | 34 | 59 | Olanzapine |
| 028 | 79 | M | Schizophrenia | 2:30 | 43 | 30 | 77 | No treatment |
| 031 | -- | -- | Schizophrenia | -- | -- | -- | -- | -- |
| 038 | 75 | M | Schizophrenia | 5:45 | 24 | 20 | 45 | Olanzapine |
| 044 | 78 | M | Schizophrenia | 7:20 | -- | -- | -- | No treatment |
| 046 | 82 | M | Schizophrenia | 6:00 | -- | -- | -- | Risperidone |
| 048 | -- | -- | Schizophrenia | -- | -- | -- | -- | -- |
| 056 | 83 | M | Schizophrenia | 4 | -- | -- | -- | Quetiapine |
| 069 | 86 | M | Schizophrenia | 5:30 | -- | -- | -- | -- |
| 074 | 85 | M | Schizophrenia | 2:15 | 22 | 21 | 26 | Haloperidol |
| 077 | 84 | M | Schizophrenia | 2:50 | 29 | 47 | 62 | Amisulpride |
| 084 | 71 | M | Schizophrenia | 7:30 | 34 | 38 | 80 | Quetiapine |
| 087 | 74 | M | Schizophrenia | 3:30 | 13 | 35 | 41 | Haloperidol and Quetiapine |
| 091 | 91 | M | Schizophrenia | 3:00 | 35 | 42 | 65 | No treatment |
| 092 | 78 | M | Schizophrenia | 6:00 | 19 | 30 | 42 | Haloperidol |
| 097 | 50 | M | Schizophrenia | 1:30 | 19 | 35 | 58 | Haloperidol |
| 100 | 69 | M | Schizophrenia | 2:40 | 16 | 22 | 41 | -- |
| 117 | 62 | M | Schizophrenia | 7:30 | 13 | 15 | 22 | Clozapine |

**Supplementary Table 2. Human RNA samples used for sRNA isolation and mouse hippocampal infusions**. For each subject, pathological diagnosis, RNA Integrity Number (RIN) and their distribution in the different Pools are detailed.

| ID | Pathological diagnosis | RIN | Pool |
| --- | --- | --- | --- |
| 1557 | Control | 5.8 | Control |
| 1679 | Control | 6 | Control |
| 1818 | Control | 4.8 | Control |
| 1858 | Control | 6.5 | Control |
| 1870 | Control | 5.9 | Control |
| 1888 | Control | 6.8 | Control |
| 1937 | Control | 5.2 | Control |
| 1949 | Control | 6.2 | Control |
| 2193 | Control | 5.1 | Control |
| 026 | Schizophrenia | 5.5 | Pool 1 |
| 027 | Schizophrenia | 7.1 | Pool 1 |
| 031 | Schizophrenia | 6.2 | Pool 1 |
| 038 | Schizophrenia | 7.5 | Pool 1 |
| 046 | Schizophrenia | 6.5 | Pool 1 |
| 048 | Schizophrenia | 6.6 | Pool 1 |
| 074 | Schizophrenia | 5.7 | Pool 1 |
| 084 | Schizophrenia | 5.3 | Pool 1 |
| 087 | Schizophrenia | 7.1 | Pool 1 |
| 097 | Schizophrenia | 7.4 | Pool 1 |
| 001 | Schizophrenia | 5.3 | Pool 2 |
| 028 | Schizophrenia | 6.8 | Pool 2 |
| 044 | Schizophrenia | 7 | Pool 2 |
| 056 | Schizophrenia | 7.3 | Pool 2 |
| 069 | Schizophrenia | 7.1 | Pool 2 |
| 077 | Schizophrenia | 6.1 | Pool 2 |
| 091 | Schizophrenia | 6.4 | Pool 2 |
| 092 | Schizophrenia | 6.4 | Pool 2 |
| 100 | Schizophrenia | 7.5 | Pool 2 |
| 117 | Schizophrenia | 5.6 | Pool 2 |

**Supplementary Table 3A. Dysregulated sRNAs in Pool 1 vs Control patients (Log2 Fold Change ≥ 0.3 and ≤ -0.3, p-value < 0.05) using SeqCluster annotation tool**.

| Annotation | Chromosomal location | Biotype | baseMean | log2FC | pvalue | padj |
| --- | --- | --- | --- | --- | --- | --- |
| hsa-miR-335 | chr7-130496125-130496182 | miRNA | 1919.51 | -0.60 | 1.76E-05 | 0.0096 |
| scRNA | chr3-49270020-49270093 | miscRNA | 133.88 | 2.82 | 0.0001 | 0.0377 |
| LINE | chr17-35462422-35462445 | Repeat | 390.65 | -0.85 | 0.0002 | 0.0452 |
| DNA | chr7-90421966-90421985 | Repeat | 267.28 | -0.92 | 0.0003 | 0.0466 |
| SINE | chrY-21331448-21331466 | Repeat | 199.51 | -0.93 | 0.0005 | 0.0509 |
| hsa-miR-128 | chr2-135665390-135665467 | miRNA | 368219.78 | -0.80 | 0.0006 | 0.0509 |
| hsa-miR-885 | chr3-10394498-10394552 | miRNA | 101.92 | -0.73 | 0.0008 | 0.0571 |
| SNORD60 | chr16-2155073-2155105 | snRNA/snoRNA | 220.88 | 0.83 | 0.0009 | 0.0571 |
| hsa-miR-184 | chr15-79209839-79209861 | miRNA | 836.70 | 2.42 | 0.0010 | 0.0571 |
| hsa-miR-876 | chr9-28863635-28863713 | miRNA | 126.45 | -1.02 | 0.0010 | 0.0571 |
| hsa-miR-135a-5p | chr12-97563833-97563893 | miRNA | 1752.28 | -0.92 | 0.0012 | 0.0581 |
| Intergenic | chr8-136090887-136090906 | Intergenic | 599.26 | -0.85 | 0.0013 | 0.0581 |
| LINC01512 | chr6-43900446-43900466 | Gene | 882.78 | -0.77 | 0.0024 | 0.0989 |
| LOC101929380 | chr5-87159890-87159913 | Gene | 132.62 | -0.90 | 0.0030 | 0.1097 |
| tRNA-Ala-CGC | chr2-156400768-156400792 | tRF | 129.26 | 1.89 | 0.0030 | 0.1097 |
| tRNA-Lys-TTT | chr1-204506522-204506599 | tRF | 231629.53 | -1.77 | 0.0034 | 0.1129 |
| Intergenic | chrM-9954-10046 | Intergenic | 69.89 | -1.29 | 0.0035 | 0.1129 |
| hsa-miR-95 | chr4-8005311-8005369 | miRNA | 6154.89 | -0.47 | 0.0047 | 0.1337 |
| Intergenic | chrM-7541-7669 | Intergenic | 77.10 | -2.14 | 0.0047 | 0.1337 |
| hsa-miR-149-5p | chr2-240456014-240456037 | miRNA | 2632.64 | -0.49 | 0.0049 | 0.1337 |
| SNORD38A | chr1-44777841-44777861 | snRNA/snoRNA | 221.68 | 0.53 | 0.0056 | 0.1384 |
| LTR | chr15-88538642-88538667 | Repeat | 71.39 | -1.28 | 0.0056 | 0.1384 |
| hsa-miR-1287 | chr10-98395231-98395292 | miRNA | 107.26 | 0.86 | 0.0059 | 0.1384 |
| hsa-miR-421 | chrX-74218370-74218467 | miRNA | 793.57 | 0.51 | 0.0063 | 0.1431 |
| hsa-miR-10b-5p | chr2-176150328-176150350 | miRNA | 62.18 | 1.23 | 0.0067 | 0.1449 |
| Intergenic | chr20-57269443-57269463 | Intergenic | 738.68 | -0.79 | 0.0069 | 0.1449 |
| tRNA-Asn-GTT | chr1-145287765-145287838 | tRF | 282.30 | 1.04 | 0.0076 | 0.1540 |
| Intergenic | chrM-5618-5879 | Intergenic | 1263.18 | -0.74 | 0.0086 | 0.1662 |
| Repeat RNA | chr6-52995815-52995914 | Repeat | 24.11 | -1.85 | 0.0087 | NA |
| hsa-miR-148a | chr7-25949919-25949981 | miRNA | 87835.59 | 1.00 | 0.0109 | 0.2028 |
| SNORD116 | chr15-25094037-25094125 | snRNA/snoRNA | 147.77 | -1.34 | 0.0117 | 0.2059 |
| SNORD116 | chr15-25097663-25097690 | snRNA/snoRNA | 44.15 | -2.07 | 0.0120 | NA |
| hsa-miR-100 | chr11-122152238-122152296 | miRNA | 63669.28 | 0.64 | 0.0122 | 0.2059 |
| SNORD98 | chr10-68755171-68755239 | snRNA/snoRNA | 77.59 | 0.74 | 0.0125 | 0.2059 |
| RNA28S, RNA45S | chrUn-113714-114242 | rRNA | 773.51 | -2.05 | 0.0130 | 0.2075 |
| hsa-let-7e | chr19-51692792-51692859 | miRNA | 1907.84 | -0.47 | 0.0133 | 0.2075 |
| LINE | chr13-110862862-110862883 | Repeat | 44.50 | -0.87 | 0.0136 | NA |
| hsa-miR-125a | chr19-51693249-51693325 | miRNA | 13542.21 | -0.42 | 0.0154 | 0.2320 |
| snRNA | chr1-16740523-16740682 | snRNA/snoRNA | 1305.48 | -1.76 | 0.0158 | 0.2320 |
| hsa-miR-532 | chrX-50003165-50003188 | miRNA | 1315.96 | 0.31 | 0.0178 | 0.2485 |
| hsa-miR-592 | chr7-127058145-127058169 | miRNA | 195.31 | -0.75 | 0.0179 | 0.2485 |
| Intergenic | chr21-31856020-31856043 | Intergenic | 295.54 | 0.97 | 0.0183 | 0.2485 |
| tRNA-Ala-TGC | chr6-28673816-28673907 | tRF | 726.27 | 1.00 | 0.0200 | 0.2657 |
| RNA18S, RNA45S | chr21-8210124-8210197 | rRNA | 78.93 | -1.62 | 0.0206 | 0.2674 |
| hsa-miR-210-3p | chr11-568110-568133 | miRNA | 402.00 | 0.66 | 0.0256 | 0.3233 |
| hsa-miR-1264 | chrX-114652654-114652717 | miRNA | 82.12 | -1.12 | 0.0275 | 0.3396 |
| tRNA-Gln-TTG | chr17-49192527-49192600 | tRF | 223.57 | -0.99 | 0.0292 | 0.3529 |
| snRNA | chr12-120291755-120291896 | snRNA/snoRNA | 86.43 | -1.17 | 0.0300 | 0.3529 |
| hsa-miR-3200 | chr22-30731568-30731631 | miRNA | 306.34 | -0.33 | 0.0305 | 0.3529 |
| RNA45S, RNA28S | chr21-8398958-8399099 | rRNA | 11.20 | -4.64 | 0.0324 | NA |
| ABCA2, ABCA2 | chr9-137007562-137007586 | Gene | 113.40 | 0.89 | 0.0325 | 0.3612 |
| PCBD2 | chr5-134925581-134925608 | Gene | 17.92 | -1.51 | 0.0331 | NA |
| SNORD82 | chr2-231460372-231460437 | snRNA/snoRNA | 488.32 | 0.61 | 0.0339 | 0.3612 |
| KCND3 | chr1-111986330-111986351 | Gene | 245.83 | 0.61 | 0.0340 | 0.3612 |
| RNA28S, RNA45S | chr21-8216670-8217303 | rRNA | 4254.79 | -2.04 | 0.0340 | 0.3612 |
| hsa-miR-651-5p | chrX-8126979-8127002 | miRNA | 113.75 | -0.70 | 0.0361 | 0.3612 |
| scRNA | chr15-30673807-30673841 | miscRNA | 129.73 | -0.95 | 0.0368 | 0.3612 |
| hsa-miR-485 | chr14-101055424-101055485 | miRNA | 1235.36 | -0.62 | 0.0372 | 0.3612 |
| RNA28S, RNA45S | chr21-8443187-8443333 | rRNA | 119.38 | -1.84 | 0.0373 | 0.3612 |
| hsa-miR-577 | chr4-114656772-114656832 | miRNA | 364.94 | 1.13 | 0.0375 | 0.3612 |
| Intergenic | chrM-15885-16064 | Intergenic | 788.50 | -0.96 | 0.0378 | 0.3612 |
| RNA28S, RNA45S | chr22-134630-134986 | rRNA | 1027.96 | 0.48 | 0.0394 | 0.3686 |
| SNORD17 | chr20-17962708-17962739 | snRNA/snoRNA | 723.66 | 0.42 | 0.0400 | 0.3686 |
| tRNA-Gln-CTG | chr15-65869050-65869133 | tRF | 2616.08 | -0.41 | 0.0408 | 0.3703 |
| scRNA | chr7-40128121-40128193 | miscRNA | 157.49 | 1.45 | 0.0425 | 0.3791 |
| Intergenic | chrM-3414-3439 | Intergenic | 124.98 | -0.84 | 0.0440 | 0.3860 |
| hsa-miR-219 | chr9-128392629-128392696 | miRNA | 29707.79 | 0.84 | 0.0456 | 0.3891 |
| hsa-miR-181b | chr9-124693723-124693781 | miRNA | 9138.32 | 0.67 | 0.0467 | 0.3891 |
| Intergenic | chrM-1569-1707 | Intergenic | 1550.53 | -0.48 | 0.0472 | 0.3891 |
| RNA18S, RNA28S, RNA45S | chr21-8209695-8210083 | rRNA | 6072.96 | -0.44 | 0.0484 | 0.3891 |
| LINE | chr11-117555248-117555268 | Repeat | 179.41 | 0.50 | 0.0487 | 0.3891 |
| SNORD49A | chr17-16440034-16440106 | snRNA/snoRNA | 682.85 | 0.91 | 0.0491 | 0.3891 |
| hsa-miR-93 | chr7-100093775-100093837 | miRNA | 385.71 | 0.51 | 0.0494 | 0.3891 |

**Supplementary Table 3B. Dysregulated sRNAs in Pool 2 vs Control patients (Log2 Fold Change ≥ 0.3 and ≤ -0.3, p-value < 0.05) using SeqCluster annotation tool**.

| Annotation | Chromosomal location | Biotype | baseMean | log2FC | pvalue | padj |
| --- | --- | --- | --- | --- | --- | --- |
| hsa-miR-148a | chr7-25949919-25949981 | miRNA | 87835.59 | 1.65 | 2.53E-05 | 0.0166 |
| Intergenic | chrM-7541-7669 | Intergenic | 77.10 | -2.69 | 0.0004 | 0.1400 |
| Intergenic | chrM-5618-5879 | Intergenic | 1263.18 | -0.95 | 0.0007 | 0.1606 |
| hsa-miR-184 | chr15-79209839-79209861 | miRNA | 836.70 | 2.40 | 0.0011 | 0.1806 |
| hsa-miR-335 | chr7-130496125-130496182 | miRNA | 1919.51 | -0.44 | 0.0017 | 0.1806 |
| hsa-miR-577 | chr4-114656772-114656832 | miRNA | 364.94 | 1.69 | 0.0019 | 0.1806 |
| hsa-miR-372-3p | chr19-53787930-53787952 | miRNA | 75.37 | -4.29 | 0.0019 | 0.1806 |
| hsa-miR-138-2-3p | chr16-56858573-56858595 | miRNA | 114.88 | 0.77 | 0.0031 | 0.2426 |
| tRNA-Ala-CGC | chr2-156400768-156400792 | tRF | 129.26 | 1.87 | 0.0033 | 0.2426 |
| Intergenic | chrM-15885-16064 | Intergenic | 788.50 | -1.27 | 0.0058 | 0.3847 |
| VTRNA1-1 | chr5-140711273-140711373 | Gene | 179.39 | -1.16 | 0.0068 | 0.4084 |
| hsa-miR-592 | chr7-127058145-127058169 | miRNA | 195.31 | -0.83 | 0.0086 | 0.4722 |
| tRNA-Pro | chr16-3184129-3184204 | tRF | 2071.30 | 1.53 | 0.0097 | 0.4914 |
| Intergenic | chrM-5900-5925 | Intergenic | 371.96 | 0.90 | 0.0106 | 0.4978 |
| Intergenic | chrM-12146-12336 | Intergenic | 9569.21 | -0.39 | 0.0124 | 0.5456 |
| LTR | chr13-37683202-37683221 | Repeat | 871.49 | 0.43 | 0.0137 | 0.5643 |
| hsa-miR-760 | chr1-93846878-93846897 | miRNA | 268.96 | -0.59 | 0.0151 | 0.5845 |
| RN7SK | chr6-52995621-52995707 | Repeat | 87.41 | -0.77 | 0.0160 | 0.5862 |
| Intergenic | chrM-2331-2378 | Intergenic | 24.64 | 1.36 | 0.0187 | 0.6127 |
| scRNA | chr1-23370253-23370285 | miscRNA | 1075.55 | 1.22 | 0.0188 | 0.6127 |
| hsa-miR-10b-5p | chr2-176150328-176150350 | miRNA | 62.18 | 1.06 | 0.0198 | 0.6127 |
| hsa-miR-874 | chr5-137647579-137647640 | miRNA | 1685.04 | -0.51 | 0.0205 | 0.6127 |
| KIAA0825 | chrM-14644-14742 | Gene | 2800.67 | -1.01 | 0.0223 | 0.6352 |
| hsa-let-7i-5p | chr12-62603671-62603712 | miRNA | 271135.92 | 0.39 | 0.0240 | 0.6352 |
| scRNA | chr3-49270020-49270093 | miscRNA | 133.88 | 1.67 | 0.0241 | 0.6352 |
| scRNA | chr17-42932881-42932942 | miscRNA | 749.28 | 1.12 | 0.0277 | 0.6906 |
| tRNA-Ala | chr6-28673816-28673907 | tRF | 726.27 | 0.93 | 0.0301 | 0.6906 |
| tRNA-Arg-ACG | chr6-26328139-26328235 | tRF | 32.92 | -0.97 | 0.0302 | 0.6906 |
| LGMN | chr14-92719226-92719337 | Repeat | 10.90 | -1.90 | 0.0305 | 0.6906 |
| LINE | chr17-35462422-35462445 | Repeat | 390.65 | -0.49 | 0.0320 | 0.6906 |
| DNA | chr7-90421966-90421985 | Repeat | 267.28 | -0.55 | 0.0325 | 0.6906 |
| tRNA-Leu-CAG | chr16-57300464-57300562 | tRF | 23.92 | 1.03 | 0.0349 | 0.7193 |
| hsa-miR-1264 | chrX-114652654-114652717 | miRNA | 82.12 | -1.06 | 0.0372 | 0.7433 |
| SNORD103C | chr1-30968164-30968234 | snRNA/snoRNA | 442.71 | 0.33 | 0.0429 | 0.8317 |
| RNA28S, RNA45S | chrUn -113714-114242 | rRNA | 773.51 | -1.66 | 0.0443 | 0.8349 |
| scRNA | chr17-42997970-42998007 | miscRNA | 426.58 | 1.01 | 0.0460 | 0.8424 |
| RNA28S, RNA45S | chr21-8217740-8217799 | rRNA | 29.73 | -1.96 | 0.0474 | 0.8447 |
| hsa-let-7d-3p | chr9-94178894-94178915 | miRNA | 145.74 | -0.83 | 0.0494 | 0.8504 |

**Supplementary Table 4A. Upregulated miRNAs in Pool 1 vs Control patients (Log2 Fold Change ≥ 0.3, p-value < 0.05) using ExceRpt annotation tool**.

| Annotation | baseMean | log2FC | pvalue | padj |
| --- | --- | --- | --- | --- |
| hsa-miR-148a-5p | 106.00 | 1.49 | 0.0001 | 0.0375 |
| hsa-miR-10b-5p | 72.86 | 1.06 | 0.0006 | 0.0866 |
| hsa-miR-548av-5p \| hsa-miR-548k | 24.93 | 0.91 | 0.0015 | 0.1320 |
| hsa-miR-1287-5p | 121.39 | 0.77 | 0.0022 | 0.1431 |
| hsa-miR-607 | 14.35 | 0.99 | 0.0053 | 0.2221 |
| hsa-let-7a-2 | 5.88 | 2.39 | 0.0081 | 0.2738 |
| hsa-miR-184 | 722.61 | 2.28 | 0.0083 | 0.2738 |
| hsa-miR-148a-3p | 66855.15 | 0.98 | 0.0092 | 0.2761 |
| hsa-miR-548ba | 21.04 | 1.69 | 0.0115 | 0.3140 |
| hsa-miR-216b-5p | 16.43 | 1.11 | 0.0182 | 0.3940 |
| hsa-let-7a-3 | 5.54 | 2.02 | 0.0185 | 0.3940 |
| hsa-miR-676-3p | 13.46 | 0.84 | 0.0195 | 0.3940 |
| hsa-miR-100-5p | 69267.08 | 0.58 | 0.0222 | 0.4154 |
| hsa-miR-152-3p | 3469.03 | 0.38 | 0.0222 | 0.4154 |
| hsa-miR-577 | 283.05 | 1.04 | 0.0262 | 0.4561 |
| hsa-miR-1249-3p | 37.86 | 0.79 | 0.0310 | 0.4662 |
| hsa-miR-421 | 705.96 | 0.53 | 0.0315 | 0.4662 |
| hsa-miR-210-3p | 516.54 | 0.58 | 0.0320 | 0.4662 |
| hsa-miR-34c-3p | 26.43 | 1.16 | 0.0323 | 0.4662 |
| hsa-miR-216a-5p | 8.45 | 1.20 | 0.0336 | 0.4662 |
| hsa-miR-2355-3p | 40.59 | 0.60 | 0.0346 | 0.4662 |
| hsa-miR-145-5p | 113.16 | 0.75 | 0.0382 | 0.4662 |
| hsa-miR-151b | 87.57 | 0.55 | 0.0385 | 0.4662 |
| hsa-miR-490-5p | 36.68 | 0.87 | 0.0436 | 0.5071 |
| hsa-miR-1911-5p | 10.47 | 1.29 | 0.0447 | 0.5071 |
| hsa-miR-193b-3p | 35.04 | 0.85 | 0.0454 | 0.5071 |

**Supplementary Table 4B. Downregulated miRNAs in Pool 1 vs Control patients (Log2 Fold Change ≤ -0.3, p-value < 0.05) using ExceRpt annotation tool**.

| Annotation | baseMean | log2FC | pvalue | padj |
| --- | --- | --- | --- | --- |
| hsa-miR-335-3p | 1125.86 | -0.83 | 1.04E-05 | 0.0065 |
| hsa-miR-337-3p | 71.12 | -0.92 | 0.0004 | 0.0848 |
| hsa-miR-876-3p | 122.62 | -1.03 | 0.0007 | 0.0866 |
| hsa-miR-128-3p | 380244.39 | -0.85 | 0.0009 | 0.0944 |
| hsa-miR-135a-5p | 1929.59 | -0.94 | 0.0023 | 0.1431 |
| hsa-miR-411-3p | 325.57 | -0.72 | 0.0024 | 0.1431 |
| hsa-miR-95-3p | 6396.38 | -0.50 | 0.0025 | 0.1431 |
| hsa-miR-149-5p | 2850.80 | -0.50 | 0.0039 | 0.1955 |
| hsa-miR-331-5p | 196.56 | -0.48 | 0.0041 | 0.1955 |
| hsa-miR-618 | 126.18 | -0.78 | 0.0049 | 0.2199 |
| hsa-miR-135b-5p | 110.46 | -0.77 | 0.0060 | 0.2226 |
| hsa-miR-5586-5p | 45.70 | -0.87 | 0.0060 | 0.2226 |
| hsa-miR-592 | 247.19 | -0.78 | 0.0093 | 0.2761 |
| hsa-miR-885-5p | 87.43 | -0.82 | 0.0097 | 0.2766 |
| hsa-let-7e-5p | 1985.66 | -0.49 | 0.0140 | 0.3650 |
| hsa-miR-376a-2-5p | 13.86 | -0.99 | 0.0150 | 0.3750 |
| hsa-miR-5701 | 4.63 | -1.42 | 0.0159 | 0.3750 |
| hsa-miR-449c-5p | 4.31 | -1.65 | 0.0162 | 0.3750 |
| hsa-miR-519a-3p | 7.89 | -1.16 | 0.0195 | 0.3940 |
| hsa-miR-485-5p | 840.21 | -0.57 | 0.0226 | 0.4154 |
| hsa-miR-222-3p | 12629.46 | -0.66 | 0.0241 | 0.4313 |
| hsa-miR-522-3p | 13.70 | -1.17 | 0.0279 | 0.4662 |
| hsa-miR-125a-5p | 13261.46 | -0.46 | 0.0314 | 0.4662 |
| hsa-miR-485-3p | 447.15 | -0.77 | 0.0315 | 0.4662 |
| hsa-miR-1264 | 78.92 | -0.90 | 0.0370 | 0.4662 |
| hsa-miR-203a-3p | 195.96 | -0.87 | 0.0374 | 0.4662 |
| hsa-miR-3681-5p | 12.52 | -0.88 | 0.0377 | 0.4662 |
| hsa-miR-5683 | 23.27 | -0.98 | 0.0387 | 0.4662 |
| hsa-miR-132-3p | 615.70 | -0.42 | 0.0438 | 0.5071 |
| hsa-miR-372-3p | 117.98 | -2.41 | 0.0471 | 0.5124 |
| hsa-miR-4443 | 6.83 | -1.24 | 0.0482 | 0.5124 |
| hsa-miR-3200-3p | 312.79 | -0.35 | 0.0483 | 0.5124 |

**Supplementary Table 4C. Upregulated miRNAs in Pool 2 vs Control patients (Log2 Fold Change ≥ 0.3, p-value < 0.05) using ExceRpt annotation tool**.

| Annotation | baseMean | log2FC | pvalue | padj |
| --- | --- | --- | --- | --- |
| hsa-miR-148a-3p | 90146.31 | 1.65 | 2.52E-07 | 0.0001 |
| hsa-miR-148a-5p | 105.03 | 1.54 | 4.24E-07 | 0.0001 |
| hsa-miR-184 | 722.74 | 2.33 | 0.0004 | 0.0793 |
| hsa-miR-607 | 16.40 | 1.33 | 0.0005 | 0.0831 |
| hsa-miR-577 | 367.27 | 1.64 | 0.0007 | 0.0919 |
| hsa-let-7c | 11.08 | 1.74 | 0.0022 | 0.1719 |
| hsa-miR-138-2-3p | 129.08 | 0.67 | 0.0043 | 0.2980 |
| hsa-miR-10b-5p | 65.26 | 0.91 | 0.0158 | 0.5212 |
| hsa-miR-1911-5p | 11.31 | 1.52 | 0.0178 | 0.5567 |
| hsa-miR-548av-5p \| hsa-miR-548k | 23.19 | 0.83 | 0.0207 | 0.5876 |
| hsa-miR-206 | 23.56 | 0.81 | 0.0290 | 0.7253 |
| hsa-let-7i-5p | 268395.06 | 0.38 | 0.0309 | 0.7311 |
| hsa-miR-548ad-5p \| hsa-miR-548ae-5p | 14.30 | 0.82 | 0.0408 | 0.8013 |
| hsa-miR-22-5p | 3942.36 | 0.31 | 0.0440 | 0.8106 |
| hsa-miR-6866-5p | 41.00 | 0.88 | 0.0494 | 0.8842 |

**Supplementary Table 4D. Downregulated miRNAs in Pool 2 vs Control patients (Log2 Fold Change ≤ -0.3, p-value < 0.05) using ExceRpt annotation tool**.

| Annotation | baseMean | log2FC | pvalue | padj |
| --- | --- | --- | --- | --- |
| hsa-miR-372-3p | 102.36 | -3.79 | 0.0011 | 0.0981 |
| hsa-miR-122-5p | 39.81 | -1.57 | 0.0011 | 0.0981 |
| hsa-miR-760 | 267.36 | -0.56 | 0.0049 | 0.3073 |
| hsa-miR-592 | 241.23 | -0.73 | 0.0055 | 0.3125 |
| hsa-miR-132-3p | 602.72 | -0.37 | 0.0084 | 0.4405 |
| hsa-miR-135a-5p | 2082.61 | -0.50 | 0.0092 | 0.4409 |
| hsa-miR-335-5p | 982.72 | -0.44 | 0.0104 | 0.4431 |
| hsa-miR-874-3p | 1718.38 | -0.47 | 0.0110 | 0.4431 |
| hsa-miR-224-5p | 72.79 | -0.94 | 0.0113 | 0.4431 |
| hsa-miR-124-5p | 93.24 | -0.64 | 0.0140 | 0.5153 |
| hsa-let-7d-3p | 171.53 | -0.77 | 0.0151 | 0.5212 |
| hsa-miR-383-5p | 475.05 | -0.75 | 0.0198 | 0.5876 |
| hsa-miR-490-3p | 221.59 | -0.62 | 0.0226 | 0.6163 |
| hsa-miR-519a-3p | 7.73 | -1.02 | 0.0288 | 0.7253 |
| hsa-miR-335-3p | 1222.57 | -0.39 | 0.0315 | 0.7311 |
| hsa-miR-1226-3p | 5.92 | -1.07 | 0.0381 | 0.8013 |
| hsa-miR-1264 | 75.39 | -0.94 | 0.0386 | 0.8013 |
| hsa-miR-522-3p | 13.85 | -0.97 | 0.0392 | 0.8013 |
| hsa-miR-129-5p | 9502.72 | -0.60 | 0.0410 | 0.8013 |
| hsa-miR-15b-5p | 28.71 | -0.61 | 0.0427 | 0.8101 |

**Supplementary Table 5A. Upregulated miRNAs in Pool 1 vs Control patients (Log2 Fold Change ≥ 0.3, p-value < 0.05) using SeqCluster annotation tool**.

| Annotation | baseMean | log2FC | pvalue | padj |
| --- | --- | --- | --- | --- |
| hsa-miR-10b-5p | 59.97 | 1.20 | 0.0008 | 0.0695 |
| hsa-miR-1287 | 114.70 | 0.82 | 0.0032 | 0.1031 |
| hsa-miR-148a | 67190.47 | 0.99 | 0.0068 | 0.1904 |
| hsa-miR-184 | 708.51 | 2.37 | 0.0086 | 0.1971 |
| hsa-miR-152 | 3405.40 | 0.39 | 0.0139 | 0.2516 |
| hsa-miR-100 | 70711.97 | 0.60 | 0.0182 | 0.2869 |
| hsa-miR-577 | 285.85 | 1.09 | 0.0227 | 0.2869 |
| hsa-miR-210-3p | 470.98 | 0.63 | 0.0265 | 0.3194 |
| hsa-miR-421 | 855.97 | 0.48 | 0.0325 | 0.3570 |

**Supplementary Table 5B. Downregulated miRNAs in Pool 1 vs Control patients (Log2 Fold Change ≤ -0.3, p-value < 0.05) using SeqCluster annotation tool**.

| Annotation | baseMean | log2FC | pvalue | padj |
| --- | --- | --- | --- | --- |
| hsa-miR-335 | 2089.51 | -0.62 | 0.0004 | 0.0627 |
| hsa-miR-128 | 388926.01 | -0.84 | 0.0005 | 0.0627 |
| hsa-miR-95 | 6498.90 | -0.48 | 0.0016 | 0.0894 |
| hsa-miR-149-5p | 2785.15 | -0.50 | 0.0018 | 0.0894 |
| hsa-miR-135a-5p | 1903.57 | -0.93 | 0.0032 | 0.1031 |
| hsa-miR-876 | 134.86 | -1.01 | 0.0033 | 0.1031 |
| hsa-miR-885 | 107.31 | -0.72 | 0.0076 | 0.1920 |
| hsa-miR-592 | 224.54 | -0.79 | 0.0113 | 0.2388 |
| hsa-miR-485 | 1232.78 | -0.63 | 0.0129 | 0.2502 |
| hsa-let-7e | 2020.00 | -0.47 | 0.0171 | 0.2869 |
| hsa-miR-1264 | 95.61 | -1.12 | 0.0201 | 0.2869 |
| hsa-miR-222 | 12521.37 | -0.65 | 0.0219 | 0.2869 |
| hsa-miR-125a | 13543.60 | -0.44 | 0.0283 | 0.3249 |
| hsa-miR-203 | 179.69 | -0.95 | 0.0383 | 0.3877 |
| hsa-miR-124 | 11213.26 | -0.49 | 0.0415 | 0.4039 |

**Supplementary Table 5C. Upregulated miRNAs in Pool 2 vs Control patients (Log2 Fold Change ≥ 0.3, p-value < 0.05) using SeqCluster annotation tool**.

| Annotation | baseMean | log2FC | pvalue | padj |
| --- | --- | --- | --- | --- |
| hsa-miR-148a | 90257.56 | 1.66 | 9.15E-08 | 2.31E-05 |
| hsa-miR-577 | 364.37 | 1.66 | 0.0005 | 0.0419 |
| hsa-miR-184 | 688.36 | 2.39 | 0.0005 | 0.0419 |
| hsa-miR-138-2-3p | 115.03 | 0.79 | 0.0017 | 0.1055 |
| hsa-miR-10b-5p | 53.15 | 1.04 | 0.0180 | 0.3785 |
| hsa-let-7i-5p | 271689.57 | 0.39 | 0.0240 | 0.4419 |

**Supplementary Table 5D. Downregulated miRNAs in Pool 2 vs Control patients (Log2 Fold Change ≤ -0.3, p-value < 0.05) using SeqCluster annotation tool**.

| Annotation | baseMean | log2FC | pvalue | padj |
| --- | --- | --- | --- | --- |
| hsa-miR-592 | 214.97 | -0.80 | 0.0043 | 0.2193 |
| hsa-miR-335 | 2123.73 | -0.42 | 0.0064 | 0.2597 |
| hsa-miR-760 | 278.65 | -0.57 | 0.0072 | 0.2597 |
| hsa-miR-874 | 1675.83 | -0.49 | 0.0097 | 0.2703 |
| hsa-miR-135a-5p | 2040.78 | -0.51 | 0.0104 | 0.2703 |
| hsa-miR-372-3p | 95.62 | -4.16 | 0.0107 | 0.2703 |
| hsa-let-7d-3p | 155.31 | -0.83 | 0.0155 | 0.3557 |
| hsa-miR-383-5p | 461.33 | -0.75 | 0.0245 | 0.4419 |
| hsa-miR-129 | 9559.20 | -0.60 | 0.0325 | 0.5481 |
| hsa-miR-1264 | 94.71 | -1.00 | 0.0472 | 0.7469 |

**Supplementary Table 6A. Upregulated genes in Pool 1 vs Control mice (Log2 Fold Change ≥ 0.3, p-value < 0.05)**.

| Gene symbol | Chromosomal location | Biotype | baseMean | log2FC | pvalue | padj |
| --- | --- | --- | --- | --- | --- | --- |
| *Gm23935* | chr16-11144125-11144181 | miRNA | 2259.45 | 2.83 | 1.53E-06 | 0.0078 |
| *Gm42418* | chr17-39846958-39848788 | lncRNA | 89954.62 | 2.64 | 2.81E-06 | 0.0115 |
| *Mir6236* | chr9-110281287-110281409 | miRNA | 112.80 | 2.14 | 7.40E-06 | 0.0252 |
| *Gm24270* | chr9-56223715-56223771 | miRNA | 118.27 | 2.72 | 2.84E-05 | 0.0726 |
| *Rn7sk* | chr9-78175303-78175633 | miscRNA | 582.43 | 3.09 | 4.10E-05 | 0.0913 |
| *Lars2* | chr9-123366927-123462666 | protein coding | 10078.79 | 2.10 | 4.89E-05 | 0.0913 |
| *Gm24187* | chr13-9834469-9834525 | miRNA | 822.87 | 2.59 | 4.91E-05 | 0.0913 |
| *n-R5-8s1* | chr18-73533403-73533550 | rRNA | 29.34 | 2.70 | 0.0001 | 0.1477 |
| *Gm24265* | chr5-115489459-115489599 | snRNA | 12.20 | 2.95 | 0.0002 | 0.2148 |
| *Gm24601* | chr6-94826781-94826923 | rRNA | 2.48 | 4.56 | 0.0002 | 0.2317 |
| *Gm26917* | chr17-39843013-39846341 | lncRNA | 624.31 | 2.08 | 0.0002 | 0.2329 |
| *Gm22009* | chr3-69085105-69085447 | scaRNA | 13.07 | 1.98 | 0.0003 | 0.2334 |
| *Gm15564* | chr16-35966752-35983230 | lncRNA | 57.85 | 1.42 | 0.0003 | 0.2676 |
| *Rny3* | chr6-47781624-47781725 | miscRNA | 22.54 | 3.02 | 0.0004 | 0.3007 |
| *Scarna2* | chr3-108554338-108554751 | scaRNA | 3.74 | 3.91 | 0.0004 | 0.3085 |
| *Gm24245* | chr4-139338304-139338360 | miRNA | 12.23 | 2.44 | 0.0005 | 0.3549 |
| *Gm22513* | chr12-54696782-54696945 | snRNA | 5.57 | 4.16 | 0.0006 | 0.3552 |
| *CT010467.1* | chr17-39846353-39848827 | rRNA | 14.12 | 2.63 | 0.0007 | 0.3960 |
| *Rny1* | chr6-47788069-47788180 | miscRNA | 69.91 | 2.65 | 0.0009 | 0.4426 |
| *Snord17* | chr2-144265979-144266216 | snoRNA | 11.15 | 2.35 | 0.0009 | 0.4426 |
| *Gm25939* | chr1-72255008-72255198 | snRNA | 6.88 | 2.39 | 0.0009 | 0.4426 |
| *Prokr2* | chr2-132337733-132385447 | protein coding | 91.71 | 0.59 | 0.0011 | 0.4459 |
| *Gm46526* | chr15-84106658-84108851 | lncRNA | 1.26 | 3.95 | 0.0013 | NA |
| *Pcdhga12* | chr18-37765580-37841873 | protein coding | 363.51 | 0.38 | 0.0013 | 0.4543 |
| *Cntnap4* | chr8-112570043-112882717 | protein coding | 527.70 | 0.36 | 0.0013 | 0.4543 |
| *Sh2d3c* | chr2-32721055-32755512 | protein coding | 308.96 | 0.34 | 0.0016 | 0.5009 |
| *Xlr4b* | chrX-73214333-73222453 | protein coding | 5.10 | 2.74 | 0.0016 | 0.5009 |
| *Abca12* | chr1-71242276-71414910 | protein coding | 3.63 | 2.79 | 0.0017 | 0.5075 |
| *5730405O15Rik* | chrX-13042015-13045297 | lncRNA | 30.53 | 0.79 | 0.0019 | 0.5206 |
| *Gm42918* | chr5-121385522-121387101 | lncRNA | 24.61 | 0.82 | 0.0022 | 0.5782 |
| *Ephb6* | chr6-41605482-41620509 | protein coding | 1722.45 | 0.30 | 0.0022 | 0.5782 |
| *Kif21b* | chr1-136131389-136177998 | protein coding | 2001.05 | 0.38 | 0.0025 | 0.6132 |
| *Palmd* | chr3-116918258-116968987 | protein coding | 526.20 | 0.39 | 0.0026 | 0.6132 |
| *Sla2* | chr2-156872457-156887192 | protein coding | 27.85 | 0.74 | 0.0027 | 0.6216 |
| *Tle3* | chr9-61372366-61418497 | protein coding | 891.79 | 0.41 | 0.0028 | 0.6216 |
| *Megf8* | chr7-25317164-25365917 | protein coding | 4504.64 | 0.31 | 0.0029 | 0.6263 |
| *Cpne7* | chr8-123117374-123135182 | protein coding | 3251.69 | 0.53 | 0.0032 | 0.6570 |
| *Stum* | chr1-180432387-180483504 | protein coding | 16189.76 | 0.34 | 0.0033 | 0.6570 |
| *Spred3* | chr7-29158829-29170411 | protein coding | 1232.88 | 0.33 | 0.0037 | 0.6776 |
| *Gm38379* | chr8-120109441-120110965 | TEC | 3.81 | 2.35 | 0.0038 | 0.6864 |
| *Dnmbp* | chr19-43846821-43940191 | protein coding | 270.85 | 0.39 | 0.0041 | 0.6975 |
| *Gm15496* | chr3-31285587-31316891 | lncRNA | 33.94 | 0.67 | 0.0045 | 0.7022 |
| *Bmf* | chr2-118528757-118549687 | protein coding | 96.86 | 0.50 | 0.0046 | 0.7022 |
| *Kcnk12* | chr17-87745801-87797994 | protein coding | 121.80 | 0.42 | 0.0053 | 0.7042 |
| *Sbpl* | chr17-23953084-23955274 | protein coding | 1.76 | 4.56 | 0.0057 | NA |
| *Siah3* | chr14-75455982-75529708 | protein coding | 229.08 | 0.35 | 0.0058 | 0.7042 |
| *Gm43429* | chr3-135860348-135862598 | TEC | 1.54 | 3.60 | 0.0058 | NA |
| *Nfatc1* | chr18-80606205-80713071 | protein coding | 133.78 | 0.35 | 0.0058 | 0.7042 |
| *Gramd2* | chr9-59680144-59718874 | protein coding | 120.35 | 0.38 | 0.0059 | 0.7042 |
| *Gm44798* | chr8-9618153-9620562 | TEC | 2.40 | 3.60 | 0.0062 | 0.7042 |
| *Col4a2* | chr8-11312805-11449287 | protein coding | 1822.11 | 0.37 | 0.0065 | 0.7042 |
| *Kcna5* | chr6-126532551-126535412 | protein coding | 21.70 | 0.98 | 0.0065 | 0.7042 |
| *Ebf4* | chr2-130295169-130370481 | protein coding | 266.65 | 0.31 | 0.0067 | 0.7042 |
| *D7Bwg0826e* | chr7-44894305-44898099 | TEC | 12.96 | 1.01 | 0.0069 | 0.7082 |
| *Ly6g6e* | chr17-35076902-35078804 | protein coding | 21.85 | 1.64 | 0.0073 | 0.7082 |
| *Tmem44* | chr16-30511855-30550842 | protein coding | 933.39 | 0.41 | 0.0076 | 0.7082 |
| *Hps1* | chr19-42755105-42779978 | protein coding | 430.40 | 0.30 | 0.0078 | 0.7082 |
| *Meltf* | chr16-31878810-31899020 | protein coding | 10.45 | 1.25 | 0.0078 | 0.7082 |
| *Gm42891* | chr3-117255132-117255820 | lncRNA | 3.01 | 2.40 | 0.0081 | 0.7082 |
| *Pcdhga2* | chr18-37668953-37841870 | protein coding | 320.43 | 0.40 | 0.0081 | 0.7082 |
| *Gm12756* | chr7-34665413-34673999 | lncRNA | 1.36 | 3.30 | 0.0081 | NA |
| *Gm50394* | chr19-53528609-53529655 | lncRNA | 8.22 | 1.31 | 0.0083 | 0.7082 |
| *Gm48129* | chr9-40341618-40342103 | lncRNA | 1.17 | 3.32 | 0.0083 | NA |
| *Kit* | chr5-75574916-75656722 | protein coding | 1705.50 | 0.35 | 0.0085 | 0.7082 |
| *Rbp3* | chr14-33954003-33964216 | protein coding | 11.47 | 2.11 | 0.0087 | 0.7082 |
| *Gm26796* | chr12-80758406-80762793 | lncRNA | 3.55 | 2.05 | 0.0089 | 0.7111 |
| *Rnu12* | chr15-83149644-83149794 | snRNA | 2.23 | 2.97 | 0.0093 | 0.7136 |
| *Ccdc88b* | chr19-6844623-6858211 | protein coding | 110.14 | 0.40 | 0.0094 | 0.7136 |
| *Vgf* | chr5-137026392-137033351 | protein coding | 3244.77 | 0.37 | 0.0097 | 0.7227 |
| *Gm43272* | chr5-101810492-101812075 | TEC | 104.57 | 0.75 | 0.0100 | 0.7358 |
| *Gm26982* | chr6-113714971-113716121 | lncRNA | 96.58 | 0.36 | 0.0105 | 0.7552 |
| *Snora73b* | chr4-132352322-132352526 | snoRNA | 11.63 | 1.26 | 0.0110 | 0.7643 |
| *Apc2* | chr10-80295977-80318263 | protein coding | 3494.47 | 0.30 | 0.0113 | 0.7643 |
| *Gm19619* | chr5-91257571-91283421 | lncRNA | 17.14 | 0.92 | 0.0114 | 0.7644 |
| *Pcnx2* | chr8-125751508-125898317 | protein coding | 2378.27 | 0.30 | 0.0116 | 0.7660 |
| *Gm16322* | chr9-42474555-42505662 | lncRNA | 2.37 | 2.36 | 0.0118 | 0.7719 |
| *Rdh1* | chr10-127759721-127768297 | protein coding | 2.31 | 2.92 | 0.0120 | 0.7781 |
| *Gm17814* | chr17-26073146-26073988 | processed pseudogene | 1.06 | 3.48 | 0.0121 | NA |
| *Plxna1* | chr6-89316314-89362620 | protein coding | 5333.11 | 0.35 | 0.0123 | 0.7923 |
| *Pcdha3* | chr18-36946105-37187657 | protein coding | 57.01 | 0.56 | 0.0124 | 0.7923 |
| *Gm10253* | chr3-88738753-88739692 | TEC | 2.44 | 2.53 | 0.0124 | 0.7923 |
| *BC006965* | chr11-112663927-112781976 | lncRNA | 24.77 | 0.64 | 0.0125 | 0.7986 |
| *Gm11730* | chr11-117094948-117097067 | lncRNA | 1.88 | 3.15 | 0.0127 | NA |
| *Gm9768* | chr4-152006759-152009619 | TEC | 11.17 | 0.93 | 0.0127 | 0.7988 |
| *Pars2* | chr4-106651069-106655282 | protein coding | 191.99 | 0.33 | 0.0134 | 0.8075 |
| *C330011M18Rik* | chr8-84065236-84067287 | lncRNA | 59.21 | 0.43 | 0.0136 | 0.8075 |
| *9130213A22Rik* | chr11-69120054-69122589 | lncRNA | 19.53 | 0.85 | 0.0136 | 0.8075 |
| *Gm25911* | chr1-167340369-167340424 | miRNA | 0.77 | 3.45 | 0.0138 | NA |
| *Tnxb* | chr17-34660457-34719815 | protein coding | 585.24 | 0.41 | 0.0140 | 0.8134 |
| *B4galnt3* | chr6-120203073-120294559 | protein coding | 31.26 | 0.71 | 0.0145 | 0.8134 |
| *C78859* | chr8-94322158-94328100 | lncRNA | 960.76 | 0.32 | 0.0148 | 0.8134 |
| *Chd5* | chr4-152338651-152390194 | protein coding | 4869.64 | 0.31 | 0.0149 | 0.8134 |
| *Kif26a* | chr12-112146208-112181747 | protein coding | 165.46 | 0.40 | 0.0151 | 0.8156 |
| *Tmem132d* | chr5-127781630-128433077 | protein coding | 343.87 | 0.41 | 0.0154 | 0.8170 |
| *Gm12473* | chr4-43631539-43641533 | lncRNA | 3.74 | 1.72 | 0.0154 | 0.8170 |
| *Helz2* | chr2-181227615-181242027 | protein coding | 126.21 | 0.45 | 0.0157 | 0.8178 |
| *Sdk1* | chr5-141241490-142215586 | protein coding | 129.93 | 0.34 | 0.0159 | 0.8178 |
| *Ddx59* | chr1-136415271-136440158 | protein coding | 174.48 | 0.31 | 0.0163 | 0.8249 |
| *Gm22175* | chr12-113425150-113425227 | miRNA | 1.53 | 2.95 | 0.0165 | NA |
| *Oxgr1* | chr14-120019585-120042435 | protein coding | 4.46 | 1.52 | 0.0166 | 0.8249 |
| *Soat2* | chr15-102150526-102163469 | protein coding | 2.36 | 2.90 | 0.0170 | 0.8278 |
| *Grtp1* | chr8-13172022-13200620 | protein coding | 70.67 | 0.42 | 0.0173 | 0.8348 |
| *Pomc* | chr12-3954951-3960642 | protein coding | 27.43 | 1.09 | 0.0178 | 0.8448 |
| *Tacstd2* | chr6-67534062-67535796 | protein coding | 1.26 | 3.12 | 0.0179 | NA |
| *Etv5* | chr16-22381309-22439719 | protein coding | 1251.93 | 0.34 | 0.0186 | 0.8557 |
| *Gm17098* | chr9-66834699-66835534 | lncRNA | 4.36 | 1.49 | 0.0186 | 0.8557 |
| *Tenm4* | chr7-96171246-96911093 | protein coding | 2349.49 | 0.42 | 0.0188 | 0.8557 |
| *Pip5kl1* | chr2-32574797-32583782 | protein coding | 54.73 | 0.50 | 0.0190 | 0.8557 |
| *6030466F02Rik* | chr8-123733958-123739941 | lncRNA | 1.17 | 3.15 | 0.0193 | NA |
| *Syt2* | chr1-134646677-134762593 | protein coding | 651.43 | 0.50 | 0.0195 | 0.8557 |
| *Gm19531* | chr9-75310335-75314871 | lncRNA | 44.15 | 0.53 | 0.0197 | 0.8557 |
| *Igsf5* | chr16-96361668-96422121 | protein coding | 3.01 | 2.00 | 0.0204 | 0.8557 |
| *Jcad* | chr18-4634878-4682869 | protein coding | 1915.67 | 0.31 | 0.0206 | 0.8557 |
| *F630040K05Rik* | chr17-45734506-45788594 | lncRNA | 25.70 | 0.60 | 0.0208 | 0.8557 |
| *Npsr1* | chr9-24097996-24316398 | protein coding | 11.62 | 1.35 | 0.0209 | 0.8557 |
| *Gm26682* | chr18-5162839-5165731 | lncRNA | 4.56 | 1.38 | 0.0210 | 0.8557 |
| *Cnga4* | chr7-105404568-105408742 | protein coding | 18.98 | 0.70 | 0.0216 | 0.8557 |
| *Fcgbp* | chr7-28071236-28120862 | protein coding | 21.97 | 0.81 | 0.0217 | 0.8557 |
| *Gm44013* | chr6-140620162-140622423 | TEC | 0.94 | 3.16 | 0.0220 | NA |
| *Ddx60* | chr8-61928087-62038244 | protein coding | 33.99 | 0.81 | 0.0221 | 0.8557 |
| *Cacna1i* | chr15-80287238-80398279 | protein coding | 1557.66 | 0.35 | 0.0221 | 0.8557 |
| *Gm38336* | chr1-34809360-34811908 | lncRNA | 4.50 | 1.70 | 0.0224 | 0.8557 |
| *Fancd2* | chr6-113531682-113597017 | protein coding | 119.22 | 0.41 | 0.0226 | 0.8557 |
| *Col5a3* | chr9-20770050-20815067 | protein coding | 125.98 | 0.52 | 0.0226 | 0.8557 |
| *Clca3a2* | chr3-144796559-144819494 | protein coding | 0.97 | 3.49 | 0.0227 | NA |
| *Gm37628* | chr1-25686062-25691501 | TEC | 1.69 | 3.00 | 0.0228 | NA |
| *Mir341* | chr12-109611500-109611595 | miRNA | 11.16 | 1.02 | 0.0229 | 0.8557 |
| *Gm18406* | chr1-112404187-112404855 | processed pseudogene | 12.91 | 1.01 | 0.0232 | 0.8557 |
| *Arhgap8* | chr15-84720052-84772207 | protein coding | 1.65 | 2.69 | 0.0236 | NA |
| *Hmcn2* | chr2-31314415-31460738 | protein coding | 18.04 | 0.69 | 0.0237 | 0.8557 |
| *Alk* | chr17-71868972-72604627 | protein coding | 139.29 | 0.37 | 0.0239 | 0.8557 |
| *Fbn1* | chr2-125300594-125507993 | protein coding | 372.75 | 0.43 | 0.0239 | 0.8557 |
| *Vpreb3* | chr10-75943057-75949657 | protein coding | 1.37 | 3.18 | 0.0242 | NA |
| *Tgm4* | chr9-123034726-123067561 | protein coding | 17.51 | 0.74 | 0.0242 | 0.8557 |
| *Pde6b* | chr5-108388391-108432397 | protein coding | 4.95 | 1.66 | 0.0247 | 0.8557 |
| *Gm30606* | chr8-93707015-93719975 | lncRNA | 1.98 | 2.60 | 0.0247 | NA |
| *Cldn23* | chr8-35824712-35826559 | protein coding | 14.09 | 1.01 | 0.0249 | 0.8557 |
| *Gm25835* | chr11-69668852-69668995 | snoRNA | 10.78 | 1.13 | 0.0258 | 0.8557 |
| *A930035D04Rik* | chr6-47453776-47454320 | pseudogene | 12.47 | 0.74 | 0.0258 | 0.8557 |
| *Prss12* | chr3-123446913-123506597 | protein coding | 298.67 | 0.58 | 0.0261 | 0.8557 |
| *Pirt* | chr11-66911981-66929876 | protein coding | 31.56 | 0.88 | 0.0261 | 0.8557 |
| *Gm12312* | chr11-70358138-70358781 | processed pseudogene | 3.78 | 2.90 | 0.0262 | 0.8557 |
| *Gm15351* | chr8-12899146-12915758 | lncRNA | 13.96 | 0.96 | 0.0265 | 0.8557 |
| *E130201H02Rik* | chr7-120597625-120598475 | processed pseudogene | 3.00 | 1.67 | 0.0268 | 0.8557 |
| *Vwa5b2* | chr16-20589471-20605377 | protein coding | 599.77 | 0.32 | 0.0270 | 0.8557 |
| *Gm43176* | chr5-52139585-52142573 | TEC | 1.48 | 2.74 | 0.0270 | NA |
| *Gm36736* | chr7-121314810-121393101 | lncRNA | 1.10 | 3.14 | 0.0270 | NA |
| *Gm6483* | chr8-19682449-19697736 | unprocessed pseudogene | 51.58 | 0.47 | 0.0273 | 0.8557 |
| *Zfp541* | chr7-16061814-16096334 | protein coding | 22.89 | 0.67 | 0.0273 | 0.8557 |
| *Mir344i* | chr7-62085223-62085310 | miRNA | 1.10 | 3.15 | 0.0280 | NA |
| *Gm47483* | chr10-29351879-29353893 | TEC | 65.76 | 0.61 | 0.0280 | 0.8557 |
| *Gm16759* | chr9-63398601-63568691 | lncRNA | 12.04 | 0.91 | 0.0282 | 0.8557 |
| *Ldoc1* | chrX-61709497-61710955 | protein coding | 39.85 | 0.48 | 0.0283 | 0.8557 |
| *G630022F23Rik* | chr5-33720043-33723669 | TEC | 11.34 | 0.79 | 0.0284 | 0.8557 |
| *Ceacam15* | chr7-16671331-16675705 | protein coding | 1.47 | 2.67 | 0.0288 | NA |
| *Gm34939* | chr15-99702664-99703976 | lncRNA | 26.05 | 0.59 | 0.0292 | 0.8557 |
| *Gm25360* | chr1-72226240-72226430 | snRNA | 1.55 | 3.90 | 0.0292 | NA |
| *Ccrl2* | chr9-111054486-111057519 | protein coding | 20.76 | 0.66 | 0.0295 | 0.8557 |
| *Tmem54* | chr4-129105548-129111626 | protein coding | 7.58 | 1.17 | 0.0301 | 0.8557 |
| *Map3k9* | chr12-81721010-81781175 | protein coding | 469.83 | 0.48 | 0.0303 | 0.8557 |
| *Gm35040* | chr7-79536043-79560033 | lncRNA | 697.65 | 0.33 | 0.0307 | 0.8557 |
| *Dennd3* | chr15-73512560-73572242 | protein coding | 128.12 | 0.37 | 0.0309 | 0.8557 |
| *Gm34680* | chr16-32550211-32553708 | lncRNA | 1.17 | 3.03 | 0.0311 | NA |
| *5730414N17Rik* | chr15-98872644-98874082 | lncRNA | 36.57 | 0.45 | 0.0311 | 0.8557 |
| *Mir6913* | chr10-81386347-81386412 | miRNA | 0.84 | 3.15 | 0.0312 | NA |
| *Otud3* | chr4-138895379-138913945 | protein coding | 174.93 | 0.34 | 0.0312 | 0.8557 |
| *Gm45178* | chr7-90995309-90999418 | TEC | 1.31 | 2.88 | 0.0315 | NA |
| *Greb1l* | chr18-10325177-10562940 | protein coding | 210.93 | 0.34 | 0.0319 | 0.8557 |
| *Tulp1* | chr17-28351515-28365222 | protein coding | 3.02 | 2.41 | 0.0324 | 0.8557 |
| *E130317F20Rik* | chr10-79851381-79854971 | lncRNA | 86.48 | 0.33 | 0.0325 | 0.8557 |
| *Gm49654* | chr12-81717830-81720052 | lncRNA | 272.04 | 0.35 | 0.0325 | 0.8557 |
| *Mstn* | chr1-53061640-53068079 | protein coding | 5.82 | 1.45 | 0.0327 | 0.8557 |
| *Gm28800* | chr1-127959516-127967452 | lncRNA | 18.31 | 0.65 | 0.0327 | 0.8557 |
| *Hkdc1* | chr10-62383137-62422491 | protein coding | 90.29 | 0.48 | 0.0327 | 0.8557 |
| *Gm47133* | chr9-122531746-122535020 | lncRNA | 4.32 | 1.52 | 0.0329 | 0.8557 |
| *Nudt8* | chr19-4000580-4002103 | protein coding | 3.24 | 1.61 | 0.0331 | 0.8557 |
| *Slc52a3* | chr2-151996511-152009258 | protein coding | 34.83 | 0.50 | 0.0334 | 0.8557 |
| *Cntnap3* | chr13-64736182-64903955 | protein coding | 114.17 | 0.40 | 0.0335 | 0.8557 |
| *2610206C17Rik* | chr7-84689640-84779053 | lncRNA | 1.20 | 2.93 | 0.0342 | NA |
| *Gm45263* | chr8-54959819-54961193 | TEC | 2.96 | 1.84 | 0.0344 | 0.8557 |
| *Xylt1* | chr7-117380979-117673580 | protein coding | 1262.52 | 0.32 | 0.0345 | 0.8557 |
| *Slc2a2* | chr3-28697903-28731359 | protein coding | 1.17 | 2.92 | 0.0345 | NA |
| *Trim34b* | chr7-104329471-104336909 | protein coding | 3.11 | 4.65 | 0.0347 | 0.8557 |
| *Alox15* | chr11-70344152-70352031 | protein coding | 1.12 | 2.97 | 0.0349 | NA |
| *Chrna1* | chr2-73563215-73580338 | protein coding | 37.64 | 0.51 | 0.0352 | 0.8557 |
| *Fras1* | chr5-96373955-96784728 | protein coding | 472.06 | 0.58 | 0.0352 | 0.8557 |
| *Tssk4* | chr14-55650184-55652539 | protein coding | 11.28 | 0.80 | 0.0354 | 0.8557 |
| *Gm35835* | chr9-41680147-41737398 | lncRNA | 7.05 | 1.04 | 0.0356 | 0.8557 |
| *Xkr8* | chr4-132724897-132732546 | protein coding | 78.71 | 0.31 | 0.0358 | 0.8557 |
| *Gm37242* | chr1-52653808-52655892 | TEC | 6.29 | 1.13 | 0.0359 | 0.8557 |
| *Zmynd12* | chr4-119422814-119467444 | protein coding | 10.72 | 0.87 | 0.0360 | 0.8557 |
| *Nxph3* | chr11-95509845-95514570 | protein coding | 147.05 | 1.50 | 0.0361 | 0.8557 |
| *Gemin4* | chr11-76210571-76217664 | protein coding | 5.02 | 1.19 | 0.0361 | 0.8557 |
| *Gm13522* | chr2-52367063-52390307 | lncRNA | 3.21 | 1.76 | 0.0362 | 0.8557 |
| *mt-Te* | chrM-14071-14139 | Mt_tRNA | 12.74 | 0.75 | 0.0363 | 0.8557 |
| *Gm19936* | chr9-56932431-56933268 | processed pseudogene | 3.27 | 1.64 | 0.0365 | 0.8557 |
| *Gm17146* | chr3-88722090-88722670 | lncRNA | 6.47 | 1.09 | 0.0365 | 0.8557 |
| *Ptges3l* | chr11-101418812-101425333 | protein coding | 14.90 | 0.75 | 0.0368 | 0.8557 |
| *6430571L13Rik* | chr9-107340602-107352422 | protein coding | 73.25 | 0.40 | 0.0374 | 0.8557 |
| *Zan* | chr5-137378637-137477064 | protein coding | 9.51 | 0.90 | 0.0375 | 0.8557 |
| *Gm42707* | chr3-143878393-143954774 | lncRNA | 2.32 | 2.66 | 0.0375 | 0.8557 |
| *Scml2* | chrX-161082525-161258213 | protein coding | 20.51 | 0.60 | 0.0378 | 0.8557 |
| *A830052D11Rik* | chr18-32359053-32378278 | lncRNA | 20.31 | 0.68 | 0.0379 | 0.8557 |
| *Mamstr* | chr7-45639977-45646521 | protein coding | 39.17 | 0.48 | 0.0380 | 0.8557 |
| *Rpl13-ps1* | chr11-85111126-85111793 | processed pseudogene | 3.71 | 1.37 | 0.0381 | 0.8557 |
| *Ank1* | chr8-22974844-23150497 | protein coding | 1063.58 | 0.34 | 0.0389 | 0.8557 |
| *Ucn2* | chr9-108986010-108987164 | protein coding | 1.24 | 2.72 | 0.0391 | NA |
| *Gm37107* | chr2-4178110-4180661 | lncRNA | 1.75 | 2.68 | 0.0392 | NA |
| *D930019O06Rik* | chr1-39650176-39657390 | lncRNA | 102.21 | 0.31 | 0.0392 | 0.8557 |
| *Klhl1* | chr14-96102736-96519102 | protein coding | 34.72 | 0.93 | 0.0394 | 0.8557 |
| *Cds1* | chr5-101765130-101823858 | protein coding | 1702.99 | 0.41 | 0.0394 | 0.8557 |
| *Bub1b* | chr2-118598211-118641591 | protein coding | 67.75 | 0.82 | 0.0394 | 0.8557 |
| *Gm48751* | chr10-9482929-9487202 | TEC | 1.26 | 2.85 | 0.0397 | NA |
| *Gm42595* | chr5-110661392-110662998 | lncRNA | 46.26 | 0.42 | 0.0397 | 0.8584 |
| *Hdc* | chr2-126593667-126619299 | protein coding | 48.16 | 0.74 | 0.0402 | 0.8589 |
| *6030407O03Rik* | chr1-73618088-73864466 | lncRNA | 7.66 | 1.06 | 0.0403 | 0.8595 |
| *Brca1* | chr11-101488764-101551955 | protein coding | 35.63 | 0.44 | 0.0404 | 0.8595 |
| *Gm42635* | chr5-57724393-57726963 | TEC | 2.12 | 1.95 | 0.0406 | NA |
| *Hapln3* | chr7-79115102-79131018 | protein coding | 24.49 | 0.52 | 0.0407 | 0.8595 |
| *Adcy9* | chr16-4287529-4420498 | protein coding | 2456.74 | 0.33 | 0.0410 | 0.8595 |
| *Fam83d* | chr2-158768093-158786637 | protein coding | 25.35 | 0.57 | 0.0411 | 0.8595 |
| *Btc* | chr5-91357261-91402994 | protein coding | 11.14 | 0.82 | 0.0412 | 0.8595 |
| *Gm2087* | chr9-83431547-83454573 | lncRNA | 1.54 | 2.66 | 0.0412 | NA |
| *Sp100* | chr1-85649988-85709998 | protein coding | 97.46 | 0.40 | 0.0414 | 0.8625 |
| *Chia1* | chr3-106113229-106132120 | protein coding | 27.61 | 0.54 | 0.0415 | 0.8625 |
| *Gpr152* | chr19-4139787-4145740 | protein coding | 1.61 | 2.58 | 0.0416 | NA |
| *Hp* | chr8-109575128-109579172 | protein coding | 3.90 | 2.57 | 0.0420 | 0.8659 |
| *Gm7074* | chr19-5324014-5325823 | processed pseudogene | 5.56 | 1.13 | 0.0425 | 0.8659 |
| *Gm45094* | chr7-35052975-35053591 | TEC | 6.26 | 1.08 | 0.0429 | 0.8659 |
| *Ccdc88c* | chr12-100911523-101029056 | protein coding | 1530.77 | 0.37 | 0.0429 | 0.8659 |
| *Gm48086* | chr10-61446902-61450717 | lncRNA | 2.53 | 1.95 | 0.0434 | 0.8659 |
| *Gm42608* | chr3-55517569-55519586 | TEC | 70.38 | 0.53 | 0.0437 | 0.8659 |
| *Gm24186* | chr10-94821529-94821655 | rRNA | 0.92 | 3.12 | 0.0437 | NA |
| *Rab43* | chr6-87788853-87812164 | protein coding | 13.54 | 0.70 | 0.0439 | 0.8659 |
| *Mirt1* | chr19-53443230-53464796 | lncRNA | 89.13 | 0.89 | 0.0441 | 0.8659 |
| *Vegfd* | chrX-164373378-164402650 | protein coding | 24.74 | 0.61 | 0.0441 | 0.8659 |
| *Gm13025* | chr4-140679753-140684617 | lncRNA | 7.39 | 0.87 | 0.0441 | 0.8659 |
| *Mir6240* | chr5-114851948-114852064 | miRNA | 2.65 | 2.32 | 0.0443 | 0.8659 |
| *Plec* | chr15-76170974-76232574 | protein coding | 4296.78 | 0.32 | 0.0445 | 0.8659 |
| *Pfkfb1* | chrX-150588229-150643878 | protein coding | 16.65 | 0.62 | 0.0447 | 0.8659 |
| *Gm13630* | chr2-66409494-66421588 | lncRNA | 23.85 | 0.55 | 0.0448 | 0.8659 |
| *F2rl1* | chr13-95511732-95525227 | protein coding | 4.11 | 1.67 | 0.0453 | 0.8671 |
| *Coro6* | chr11-77462411-77470484 | protein coding | 482.68 | 0.49 | 0.0456 | 0.8675 |
| *Ndst4* | chr3-125404076-125728899 | protein coding | 994.33 | 0.48 | 0.0458 | 0.8675 |
| *Gm27320* | chr8-105307792-105307900 | miRNA | 7.79 | 1.13 | 0.0459 | 0.8675 |
| *Plekha7* | chr7-116123485-116308376 | protein coding | 380.67 | 0.46 | 0.0459 | 0.8675 |
| *Gm6569* | chr15-73834561-73839908 | protein coding | 2.02 | 2.19 | 0.0459 | NA |
| *Gjd2* | chr2-114009601-114013619 | protein coding | 101.18 | 0.32 | 0.0459 | 0.8675 |
| *Gm47585* | chr13-63273600-63275911 | TEC | 1.13 | 2.66 | 0.0461 | NA |
| *Gm25395* | chr1-87776938-87777209 | scaRNA | 9.16 | 1.71 | 0.0462 | 0.8686 |
| *Mcm10* | chr2-4989714-5012791 | protein coding | 14.27 | 0.72 | 0.0462 | 0.8686 |
| *Gm15483* | chr7-133738479-133738934 | processed pseudogene | 1.41 | 2.70 | 0.0464 | NA |
| *Gm13470* | chr2-46419535-46442710 | lncRNA | 4.86 | 1.32 | 0.0464 | 0.8686 |
| *Cacna1f* | chrX-7607083-7635196 | protein coding | 3.23 | 1.55 | 0.0464 | 0.8686 |
| *Gm49674* | chr16-74873039-74875938 | TEC | 1.46 | 2.55 | 0.0468 | NA |
| *Proser2* | chr2-6097607-6130211 | protein coding | 35.72 | 0.49 | 0.0471 | 0.8686 |
| *Pvrig* | chr5-138341961-138343557 | protein coding | 2.16 | 2.04 | 0.0472 | 0.8686 |
| *Tas2r137* | chr6-40491233-40492305 | protein coding | 0.95 | 2.76 | 0.0473 | NA |
| *Mylpf* | chr7-127208890-127214298 | protein coding | 16.97 | 0.64 | 0.0474 | 0.8686 |
| *Zfp46* | chr4-136284683-136293942 | protein coding | 1302.17 | 0.31 | 0.0474 | 0.8686 |
| *Gm39326* | chr9-44877891-44884397 | lncRNA | 69.04 | 0.51 | 0.0477 | 0.8694 |
| *Gm43046* | chr5-49069010-49071460 | TEC | 2.95 | 1.87 | 0.0477 | 0.8694 |
| *Adamtsl5* | chr10-80339110-80348412 | protein coding | 88.81 | 0.38 | 0.0478 | 0.8694 |
| *Dio3os* | chr12-110275385-110278172 | lncRNA | 2.53 | 1.99 | 0.0481 | 0.8702 |
| *Mfsd4b3-ps* | chr10-39946912-39960184 | transcribed unprocessed pseudogene | 1.12 | 2.74 | 0.0489 | NA |
| *Atad3aos* | chr4-155761178-155769264 | lncRNA | 19.49 | 0.65 | 0.0492 | 0.8702 |
| *Htr6* | chr4-139061108-139075570 | protein coding | 91.37 | 0.34 | 0.0495 | 0.8702 |
| *Gm19410* | chr8-35765790-35818047 | protein coding | 519.91 | 0.64 | 0.0498 | 0.8702 |

**Supplementary Table 6B. Downregulated genes in Pool 1 vs Control mice (Log2 Fold Change ≤ -0.3, p-value < 0.05)**.

| Gene symbol | Chromosomal location | Biotype | baseMean | log2FC | pvalue | padj |
| --- | --- | --- | --- | --- | --- | --- |
| *Eef1a1-ps1* | chr19-17926623-17928009 | processed pseudogene | 61.11 | -2.24 | 8.60E-09 | 0.0001 |
| *Gm5869* | chr5-86997159-86998415 | processed pseudogene | 25.41 | -5.31 | 1.12E-08 | 0.0001 |
| *Gm13456* | chr2-40555752-40557116 | processed pseudogene | 218.43 | -2.32 | 2.31E-07 | 0.0016 |
| *Rpl37rt* | chr5-115102923-115110268 | transcribed processed pseudogene | 159.72 | -0.67 | 6.07E-05 | 0.0999 |
| *H3f3a* | chr1-180800832-180813943 | protein coding | 900.57 | -0.44 | 6.35E-05 | 0.0999 |
| *2610001J05Rik* | chr6-13867735-13871518 | protein coding | 681.92 | -0.42 | 0.0001 | 0.1870 |
| *Mrrf* | chr2-36136389-36190647 | protein coding | 370.35 | -0.32 | 0.0002 | 0.2094 |
| *Gbx2* | chr1-89927956-89931179 | protein coding | 5.34 | -4.93 | 0.0002 | 0.2334 |
| *Tph2* | chr10-115078641-115185022 | protein coding | 57.87 | -0.70 | 0.0003 | 0.2334 |
| *Otx2* | chr14-48657677-48673819 | protein coding | 94.01 | -1.28 | 0.0003 | 0.2601 |
| *Med21* | chr6-146642547-146650732 | protein coding | 413.00 | -0.41 | 0.0005 | 0.3435 |
| *Cxcl5* | chr5-90759360-90761624 | protein coding | 28.78 | -1.48 | 0.0006 | 0.3552 |
| *3632454L22Rik* | chrX-135022734-135060336 | lncRNA | 19.75 | -1.07 | 0.0006 | 0.3556 |
| *Rpl3-ps1* | chrX-13202571-13203780 | processed pseudogene | 110.05 | -2.26 | 0.0008 | 0.4426 |
| *H3c15* | chr3-96238108-96239127 | protein coding | 36.39 | -0.91 | 0.0009 | 0.4426 |
| *Ctxn3* | chr18-57468516-57478133 | protein coding | 38.04 | -1.63 | 0.0009 | 0.4426 |
| *Tmem126a* | chr7-90450700-90457229 | protein coding | 490.75 | -0.38 | 0.0009 | 0.4426 |
| *Gfap* | chr11-102887336-102900912 | protein coding | 12820.26 | -0.48 | 0.0010 | 0.4459 |
| *2810006K23Rik* | chr5-124328089-124341844 | protein coding | 278.22 | -0.35 | 0.0010 | 0.4459 |
| *Rps16-ps2* | chr5-129128077-129128517 | processed pseudogene | 61.03 | -0.85 | 0.0010 | 0.4459 |
| *Acyp1* | chr12-85272398-85288438 | protein coding | 303.26 | -0.38 | 0.0011 | 0.4459 |
| *Cript* | chr17-87025550-87035810 | protein coding | 943.23 | -0.32 | 0.0011 | 0.4459 |
| *Etfrf1* | chr6-145211134-145216939 | protein coding | 449.84 | -0.32 | 0.0011 | 0.4459 |
| *Pcp4* | chr16-96467606-96525793 | protein coding | 175.28 | -0.64 | 0.0012 | 0.4459 |
| *Rpl38-ps2* | chr6-140746960-140747172 | processed pseudogene | 38.77 | -0.86 | 0.0012 | 0.4459 |
| *Hopx* | chr5-77086988-77115121 | protein coding | 1575.80 | -0.38 | 0.0012 | 0.4484 |
| *Gm2000* | chr1-156366040-156366484 | protein coding | 54.65 | -0.81 | 0.0012 | 0.4484 |
| *Cetn2* | chrX-72913532-72918411 | protein coding | 717.15 | -0.30 | 0.0012 | 0.4484 |
| *Sptssa* | chr12-54645392-54656573 | protein coding | 448.35 | -0.38 | 0.0014 | 0.4844 |
| *Anapc10* | chr8-79711820-79779059 | protein coding | 238.75 | -0.31 | 0.0015 | 0.4844 |
| *Bbip1* | chr19-53929861-53944627 | protein coding | 1035.71 | -0.32 | 0.0017 | 0.5075 |
| *Gm5619* | chr9-85781868-85782627 | processed pseudogene | 3.63 | -2.62 | 0.0017 | 0.5075 |
| *Gm11273* | chr13-21501029-21501418 | protein coding | 42.29 | -0.76 | 0.0017 | 0.5075 |
| *Lcn2* | chr2-32384633-32388252 | protein coding | 13.54 | -4.06 | 0.0018 | 0.5075 |
| *Mrpl32* | chr13-14608184-14613165 | protein coding | 414.89 | -0.30 | 0.0018 | 0.5075 |
| *Zfp521* | chr18-13687013-13972787 | protein coding | 246.24 | -0.31 | 0.0018 | 0.5206 |
| *Gm10443* | chr6-86334031-86334240 | processed pseudogene | 37.50 | -0.91 | 0.0022 | 0.5782 |
| *Gm28153* | chr1-17741915-17805417 | lncRNA | 1.33 | -3.99 | 0.0022 | NA |
| *Zic1* | chr9-91358058-91365810 | protein coding | 611.83 | -1.12 | 0.0022 | 0.5782 |
| *Gm17750* | chr13-84025297-84064772 | lncRNA | 90.93 | -0.67 | 0.0022 | 0.5782 |
| *Chat* | chr14-32408203-32465989 | protein coding | 5.41 | -3.89 | 0.0024 | 0.6044 |
| *sep-10* | chr10-59141627-59221847 | protein coding | 138.62 | -0.37 | 0.0024 | 0.6049 |
| *Gm5539* | chr3-59767275-59768240 | processed pseudogene | 1.21 | -3.91 | 0.0025 | NA |
| *Ormdl1* | chr1-53297095-53310309 | protein coding | 108.00 | -0.43 | 0.0026 | 0.6132 |
| *Zcchc10* | chr11-53324679-53333301 | protein coding | 179.91 | -0.42 | 0.0026 | 0.6132 |
| *Lbhd2* | chr12-111406809-111412090 | protein coding | 19.31 | -1.93 | 0.0027 | 0.6216 |
| *Rpl19-ps11* | chr8-19492936-19493526 | processed pseudogene | 46.36 | -0.57 | 0.0029 | 0.6263 |
| *Gm9843* | chr16-76403251-76403652 | processed pseudogene | 54.39 | -0.76 | 0.0029 | 0.6263 |
| *Med6* | chr12-81573557-81595008 | protein coding | 367.68 | -0.33 | 0.0029 | 0.6263 |
| *Cd59a* | chr2-104095801-104115354 | protein coding | 90.85 | -0.64 | 0.0030 | 0.6373 |
| *Gm10288* | chr3-146838811-146839347 | processed pseudogene | 60.31 | -0.50 | 0.0031 | 0.6508 |
| *Magt1* | chrX-105968084-106011906 | protein coding | 398.29 | -0.41 | 0.0032 | 0.6570 |
| *Gm10275* | chr10-29698746-29699380 | transcribed processed pseudogene | 45.12 | -0.74 | 0.0036 | 0.6776 |
| *Pdcd2* | chr17-15519208-15527301 | protein coding | 211.68 | -0.42 | 0.0036 | 0.6776 |
| *Gm13341* | chr2-22587291-22587962 | unprocessed pseudogene | 75.77 | -0.98 | 0.0036 | 0.6776 |
| *Nfe2l2* | chr2-75675513-75704641 | protein coding | 409.06 | -0.30 | 0.0037 | 0.6776 |
| *Gm28437* | chr1-24613189-24613971 | unprocessed pseudogene | 77.27 | -0.92 | 0.0037 | 0.6776 |
| *Otos* | chr1-92644218-92648841 | protein coding | 44.28 | -1.33 | 0.0038 | 0.6864 |
| *Ak6* | chr13-100650979-100666359 | protein coding | 137.88 | -0.40 | 0.0039 | 0.6872 |
| *Gm13436* | chr2-36807826-36808392 | processed pseudogene | 26.76 | -0.75 | 0.0040 | 0.6882 |
| *Gm10086* | chr5-108517179-108517532 | processed pseudogene | 9.36 | -1.37 | 0.0040 | 0.6893 |
| *A230056P14Rik* | chr7-55962531-55980868 | lncRNA | 54.39 | -0.54 | 0.0042 | 0.6987 |
| *Immp1l* | chr2-105904638-105965558 | protein coding | 217.95 | -0.40 | 0.0043 | 0.7022 |
| *Baiap2l1* | chr5-144264526-144358112 | protein coding | 29.47 | -0.90 | 0.0043 | 0.7022 |
| *Iltifb* | chr10-118289629-118295038 | protein coding | 2.30 | -3.78 | 0.0044 | 0.7022 |
| *Rpl17-ps4* | chr4-63895545-63896099 | processed pseudogene | 2.17 | -3.19 | 0.0045 | 0.7022 |
| *Syt9* | chr7-107370728-107548656 | protein coding | 151.83 | -0.84 | 0.0045 | 0.7022 |
| *Snrnp27* | chr6-86675151-86684522 | protein coding | 793.26 | -0.31 | 0.0045 | 0.7022 |
| *Zfp133-ps* | chr2-144459280-144500267 | transcribed_unitary_pseudogene | 70.00 | -0.47 | 0.0046 | 0.7022 |
| *Gm14303* | chr2-172509521-172509691 | processed pseudogene | 164.56 | -0.79 | 0.0047 | 0.7022 |
| *Otx2os1* | chr14-48668708-48870683 | lncRNA | 5.54 | -1.99 | 0.0047 | 0.7022 |
| *Ost4* | chr5-30905885-30907788 | protein coding | 490.37 | -0.37 | 0.0048 | 0.7022 |
| *Gstk1* | chr6-42245935-42250447 | protein coding | 190.64 | -0.46 | 0.0048 | 0.7022 |
| *Mgst1* | chr6-138140316-138156755 | protein coding | 187.00 | -0.40 | 0.0049 | 0.7022 |
| *Gm45871* | chr18-90579777-90592528 | protein coding | 204.45 | -0.32 | 0.0049 | 0.7025 |
| *Snapc5* | chr9-64179274-64183132 | protein coding | 285.97 | -0.36 | 0.0049 | 0.7025 |
| *Tdgf1* | chr9-110939603-110946158 | protein coding | 18.99 | -0.92 | 0.0051 | 0.7042 |
| *Kif18a* | chr2-109280738-109341747 | protein coding | 29.86 | -0.75 | 0.0051 | 0.7042 |
| *Ciao2a* | chr9-66126611-66138955 | protein coding | 401.39 | -0.30 | 0.0052 | 0.7042 |
| *Gm26403* | chr18-61138509-61138613 | snRNA | 1.72 | -3.39 | 0.0053 | NA |
| *Rps26* | chr10-128624534-128626747 | protein coding | 1823.19 | -0.36 | 0.0054 | 0.7042 |
| *Rps24-ps3* | chrX-79195047-79195448 | processed pseudogene | 102.00 | -0.67 | 0.0054 | 0.7042 |
| *Tpt1* | chr14-75845093-75848525 | protein coding | 5563.18 | -0.41 | 0.0054 | 0.7042 |
| *Gpx4-ps2* | chr10-45875291-45876086 | transcribed processed pseudogene | 64.44 | -0.81 | 0.0054 | 0.7042 |
| *H2bc4* | chr13-23684199-23692908 | protein coding | 544.47 | -0.42 | 0.0055 | 0.7042 |
| *Rpl36a* | chrX-134585654-134588062 | protein coding | 905.55 | -0.35 | 0.0055 | 0.7042 |
| *Lsm2* | chr17-34981862-34985891 | protein coding | 161.49 | -0.35 | 0.0057 | 0.7042 |
| *Zic3* | chrX-58022700-58041736 | protein coding | 76.69 | -0.61 | 0.0058 | 0.7042 |
| *Zic4* | chr9-91362413-91389348 | protein coding | 138.98 | -0.85 | 0.0058 | 0.7042 |
| *Gm6444* | chr5-123066242-123066699 | processed pseudogene | 11.58 | -1.25 | 0.0060 | 0.7042 |
| *Gm29216* | chr1-24615706-24616197 | unprocessed pseudogene | 74.50 | -0.81 | 0.0061 | 0.7042 |
| *Cthrc1* | chr15-39076932-39087121 | protein coding | 120.69 | -0.43 | 0.0061 | 0.7042 |
| *Il7r* | chr15-9505788-9530176 | protein coding | 13.72 | -1.00 | 0.0062 | 0.7042 |
| *Snord89* | chr1-39548745-39548854 | snoRNA | 8.34 | -1.29 | 0.0062 | 0.7042 |
| *F630042J09Rik* | chr13-67278577-67283361 | lncRNA | 8.45 | -1.56 | 0.0063 | 0.7042 |
| *Lin9* | chr1-180641150-180690694 | protein coding | 103.37 | -0.46 | 0.0064 | 0.7042 |
| *Gm20383* | chr6-81984321-81992413 | lncRNA | 23.65 | -0.86 | 0.0065 | 0.7042 |
| *Rpp30* | chr19-36083716-36104777 | protein coding | 256.24 | -0.34 | 0.0065 | 0.7042 |
| *Smim22* | chr16-5007288-5008309 | protein coding | 2.91 | -2.90 | 0.0065 | 0.7042 |
| *Gm26793* | chr7-144894560-144897461 | lncRNA | 4.01 | -2.13 | 0.0067 | 0.7042 |
| *Tuba1c* | chr15-99029891-99038110 | protein coding | 31.72 | -0.98 | 0.0067 | 0.7042 |
| *Nit2* | chr16-57156665-57167341 | protein coding | 186.54 | -0.34 | 0.0067 | 0.7042 |
| *Gm28661* | chr1-24614885-24615565 | unprocessed pseudogene | 103.29 | -0.89 | 0.0068 | 0.7082 |
| *Wfdc2* | chr2-164562413-164568510 | protein coding | 12.46 | -1.96 | 0.0070 | 0.7082 |
| *9330198I05Rik* | chr5-108360187-108363885 | lncRNA | 6.93 | -1.38 | 0.0070 | 0.7082 |
| *Gm21814* | chr6-149566090-149583656 | lncRNA | 7.89 | -1.30 | 0.0070 | 0.7082 |
| *Spata1* | chr3-146457196-146499753 | protein coding | 48.16 | -0.59 | 0.0071 | 0.7082 |
| *H4c9* | chr13-22040636-22041362 | protein coding | 46.30 | -0.57 | 0.0072 | 0.7082 |
| *F2rl3* | chr8-72761880-72763874 | protein coding | 3.48 | -2.29 | 0.0072 | 0.7082 |
| *Smim11* | chr16-92301286-92313041 | protein coding | 175.21 | -0.46 | 0.0074 | 0.7082 |
| *Gm7172* | chr12-51133551-51134702 | processed pseudogene | 11.91 | -1.05 | 0.0076 | 0.7082 |
| *2310009A05Rik* | chr9-73039718-73042779 | protein coding | 129.82 | -0.44 | 0.0076 | 0.7082 |
| *Sycp1* | chr3-102818499-102936100 | protein coding | 2.67 | -3.23 | 0.0077 | 0.7082 |
| *Zeb2os* | chr2-45110707-45114087 | lncRNA | 54.63 | -0.49 | 0.0077 | 0.7082 |
| *Pdia4* | chr6-47796141-47813430 | protein coding | 969.15 | -0.31 | 0.0077 | 0.7082 |
| *Rida* | chr15-34484021-34495255 | protein coding | 825.61 | -0.35 | 0.0081 | 0.7082 |
| *Irx1* | chr13-71957921-71963723 | protein coding | 15.42 | -1.34 | 0.0081 | 0.7082 |
| *Hspa5* | chr2-34771970-34777547 | protein coding | 5501.39 | -0.31 | 0.0082 | 0.7082 |
| *Pnp* | chr14-50931082-50965237 | protein coding | 93.49 | -0.55 | 0.0083 | 0.7082 |
| *Cox7b* | chrX-106015700-106022450 | protein coding | 2774.16 | -0.38 | 0.0086 | 0.7082 |
| *Gm12338* | chr11-75599763-75599954 | processed pseudogene | 45.68 | -0.78 | 0.0086 | 0.7082 |
| *Thrsp* | chr7-97412938-97417730 | protein coding | 676.10 | -0.32 | 0.0087 | 0.7082 |
| *Gm26512* | chr12-90738201-90869235 | lncRNA | 53.86 | -0.48 | 0.0087 | 0.7082 |
| *4921524J17Rik* | chr8-85408759-85432833 | protein coding | 264.09 | -0.32 | 0.0087 | 0.7082 |
| *Rpa3* | chr6-8255936-8259173 | protein coding | 140.33 | -0.43 | 0.0087 | 0.7082 |
| *Cst7* | chr2-150570415-150578944 | protein coding | 18.94 | -1.85 | 0.0088 | 0.7082 |
| *Mtln* | chr2-127791388-127792488 | protein coding | 546.42 | -0.35 | 0.0089 | 0.7111 |
| *Lhx1os* | chr11-84525660-84535831 | lncRNA | 8.75 | -1.39 | 0.0089 | 0.7111 |
| *Cd9* | chr6-125460266-125494791 | protein coding | 1243.09 | -0.49 | 0.0092 | 0.7136 |
| *Cpq* | chr15-33083129-33594552 | protein coding | 260.54 | -0.32 | 0.0092 | 0.7136 |
| *Lif* | chr11-4257557-4272514 | protein coding | 23.80 | -0.69 | 0.0092 | 0.7136 |
| *Dctpp1* | chr7-127256959-127260709 | protein coding | 150.49 | -0.41 | 0.0094 | 0.7136 |
| *Rpl38* | chr11-114668524-114672331 | protein coding | 1794.03 | -0.38 | 0.0095 | 0.7136 |
| *Bud31* | chr5-145140362-145148078 | protein coding | 816.24 | -0.36 | 0.0095 | 0.7136 |
| *Creld2* | chr15-88819646-88826683 | protein coding | 294.67 | -0.35 | 0.0096 | 0.7161 |
| *Gm5210* | chr15-8917798-8929171 | processed pseudogene | 1.06 | -3.57 | 0.0097 | NA |
| *Scgn* | chr13-23953456-23991214 | protein coding | 51.45 | -0.60 | 0.0102 | 0.7448 |
| *Gm35857* | chr8-72531031-72531208 | processed pseudogene | 6.92 | -1.33 | 0.0102 | 0.7448 |
| *Gm11808* | chr4-3973092-3973595 | protein coding | 51.15 | -0.61 | 0.0106 | 0.7552 |
| *Gm4540* | chr3-106034664-106035092 | processed pseudogene | 9.06 | -1.28 | 0.0107 | 0.7552 |
| *Asf1a* | chr10-53596757-53609225 | protein coding | 234.49 | -0.31 | 0.0107 | 0.7552 |
| *Gm43398* | chr5-143395503-143398244 | TEC | 73.00 | -0.96 | 0.0109 | 0.7632 |
| *Cebpzos* | chr17-78916500-78920306 | protein coding | 324.05 | -0.33 | 0.0110 | 0.7643 |
| *Llph* | chr10-120227070-120232582 | protein coding | 179.51 | -0.33 | 0.0111 | 0.7643 |
| *Gm10222* | chr1-24612407-24612700 | unprocessed pseudogene | 4.92 | -1.74 | 0.0113 | 0.7643 |
| *Gm15577* | chr3-156903863-156920780 | lncRNA | 2.63 | -2.76 | 0.0113 | 0.7643 |
| *AI987944* | chr7-41372923-41393379 | protein coding | 219.04 | -0.30 | 0.0114 | 0.7644 |
| *Gm10925* | chr1-24613974-24614651 | unprocessed pseudogene | 127.92 | -0.84 | 0.0114 | 0.7644 |
| *Gpr84* | chr15-103308235-103310612 | protein coding | 37.55 | -0.80 | 0.0118 | 0.7719 |
| *Mkks* | chr2-136873780-136891389 | protein coding | 263.45 | -0.36 | 0.0127 | 0.7988 |
| *Gm13594* | chr2-65238756-65239675 | lncRNA | 2.52 | -2.11 | 0.0127 | 0.7988 |
| *Gm9349* | chr17-74329533-74329888 | processed pseudogene | 4.62 | -1.72 | 0.0128 | 0.7988 |
| *Ormdl2* | chr10-128817902-128821645 | protein coding | 122.26 | -0.45 | 0.0129 | 0.8040 |
| *Gm6789* | chr18-63543751-63545135 | processed pseudogene | 6.99 | -1.52 | 0.0132 | 0.8075 |
| *Cenpx* | chr11-120710942-120713738 | protein coding | 163.25 | -0.43 | 0.0134 | 0.8075 |
| *Rpl31-ps8* | chr15-90764279-90764656 | processed pseudogene | 44.75 | -0.57 | 0.0135 | 0.8075 |
| *Manf* | chr9-106838312-106891979 | protein coding | 933.36 | -0.37 | 0.0137 | 0.8075 |
| *Tomm5* | chr4-45105208-45108114 | protein coding | 375.57 | -0.36 | 0.0139 | 0.8134 |
| *Serpine1* | chr5-137061504-137072268 | protein coding | 10.87 | -1.05 | 0.0140 | 0.8134 |
| *Gm14866* | chrX-100736983-100740929 | lncRNA | 3.35 | -2.63 | 0.0141 | 0.8134 |
| *Npy5r* | chr8-66679965-66688128 | protein coding | 184.81 | -0.33 | 0.0144 | 0.8134 |
| *Map2k3os* | chr11-60920917-60931867 | lncRNA | 20.82 | -0.75 | 0.0144 | 0.8134 |
| *B430218F22Rik* | chr13-118386630-118388124 | lncRNA | 4.10 | -1.55 | 0.0145 | 0.8134 |
| *Scx* | chr15-76457452-76459458 | protein coding | 9.18 | -1.24 | 0.0145 | 0.8134 |
| *Ly86* | chr13-37345208-37419036 | protein coding | 330.37 | -0.48 | 0.0145 | 0.8134 |
| *Snhg5* | chr9-88495268-88523562 | lncRNA | 193.04 | -0.35 | 0.0145 | 0.8134 |
| *Pter* | chr2-12924041-13003455 | protein coding | 48.26 | -0.50 | 0.0146 | 0.8134 |
| *Slc6a5* | chr7-49910146-49963856 | protein coding | 9.38 | -1.21 | 0.0147 | 0.8134 |
| *Frrs1* | chr3-116859464-116908177 | protein coding | 70.26 | -0.46 | 0.0147 | 0.8134 |
| *Aard* | chr15-52040107-52045722 | protein coding | 40.22 | -0.56 | 0.0148 | 0.8134 |
| *Nrk* | chrX-138914430-139010532 | protein coding | 9.54 | -1.44 | 0.0148 | 0.8134 |
| *Trdn* | chr10-33080554-33476709 | protein coding | 3.83 | -2.01 | 0.0148 | 0.8134 |
| *Cd180* | chr13-102693558-102739629 | protein coding | 98.88 | -0.50 | 0.0148 | 0.8134 |
| *Gm5089* | chr14-122365239-122406743 | lncRNA | 117.52 | -0.41 | 0.0149 | 0.8134 |
| *Rpl36a-ps2* | chr3-145879020-145879340 | processed pseudogene | 37.54 | -0.72 | 0.0150 | 0.8156 |
| *S100a1* | chr3-90511034-90514392 | protein coding | 911.29 | -0.35 | 0.0156 | 0.8178 |
| *Tpt1-ps3* | chr6-99877816-99878334 | processed pseudogene | 166.83 | -0.49 | 0.0156 | 0.8178 |
| *Lrrc1* | chr9-77430823-77544870 | protein coding | 189.07 | -0.44 | 0.0157 | 0.8178 |
| *Acyp2* | chr11-30505991-30649587 | protein coding | 335.73 | -0.40 | 0.0160 | 0.8212 |
| *Gm39465* | chr9-122019528-122051463 | lncRNA | 1.83 | -2.76 | 0.0161 | NA |
| *Ttr* | chr18-20665280-20674321 | protein coding | 10056.97 | -2.19 | 0.0161 | 0.8212 |
| *Spata4* | chr8-54600781-54610098 | protein coding | 1.34 | -2.99 | 0.0161 | NA |
| *Gm6170* | chr1-128520073-128521460 | processed pseudogene | 8.12 | -1.34 | 0.0164 | 0.8249 |
| *Gm15500* | chr7-114705217-114706235 | transcribed processed pseudogene | 60.87 | -0.50 | 0.0166 | 0.8249 |
| *Snrpd2* | chr7-19149722-19153542 | protein coding | 604.74 | -0.33 | 0.0166 | 0.8249 |
| *Gm14017* | chr2-121481129-121481514 | processed pseudogene | 2.60 | -2.58 | 0.0167 | 0.8255 |
| *Ripply3* | chr16-94328420-94336935 | protein coding | 3.96 | -1.65 | 0.0168 | 0.8271 |
| *Mterf1b* | chr5-4192367-4197651 | protein coding | 37.62 | -0.54 | 0.0168 | 0.8271 |
| *Gm48122* | chr12-4077403-4077624 | processed pseudogene | 6.39 | -1.84 | 0.0169 | 0.8271 |
| *Asb5* | chr8-54529580-54587875 | protein coding | 11.95 | -0.89 | 0.0170 | 0.8274 |
| *Rps19-ps6* | chr12-110898114-110898551 | processed pseudogene | 11.54 | -0.92 | 0.0171 | 0.8307 |
| *Ndufb4* | chr16-37647170-37654453 | protein coding | 244.80 | -0.30 | 0.0174 | 0.8381 |
| *Gm11836* | chr4-14433891-14434331 | processed pseudogene | 1.54 | -3.38 | 0.0176 | NA |
| *Gm5855* | chr3-130929366-130930670 | processed pseudogene | 4.24 | -1.78 | 0.0179 | 0.8501 |
| *Gm15421* | chr5-22528321-22529244 | transcribed processed pseudogene | 14.19 | -0.83 | 0.0182 | 0.8557 |
| *Klk6* | chr7-43824499-43832030 | protein coding | 60.04 | -1.32 | 0.0183 | 0.8557 |
| *Olfr570* | chr7-102894577-102901710 | protein coding | 1.72 | -3.59 | 0.0184 | NA |
| *Chrna6* | chr8-27403212-27413944 | protein coding | 9.53 | -1.79 | 0.0184 | 0.8557 |
| *Rpf2* | chr10-40223246-40247036 | protein coding | 442.92 | -0.33 | 0.0185 | 0.8557 |
| *Gm11478* | chr11-86630428-86631026 | processed pseudogene | 66.68 | -0.58 | 0.0188 | 0.8557 |
| *Shox2* | chr3-66971727-66981771 | protein coding | 59.42 | -1.93 | 0.0192 | 0.8557 |
| *Gm17511* | chr7-127286044-127286385 | processed pseudogene | 17.33 | -1.46 | 0.0192 | 0.8557 |
| *Esd* | chr14-74732297-74750765 | protein coding | 1216.06 | -0.31 | 0.0193 | 0.8557 |
| *Gm36742* | chr16-39187463-39188906 | lncRNA | 61.09 | -0.57 | 0.0194 | 0.8557 |
| *Gm7027* | chr7-101573460-101574321 | processed pseudogene | 6.95 | -1.18 | 0.0195 | 0.8557 |
| *Rps24* | chr14-24487125-24496959 | protein coding | 3916.61 | -0.35 | 0.0196 | 0.8557 |
| *Gm19196* | chr12-21543307-21554521 | transcribed_unprocessed pseudogene | 81.55 | -0.46 | 0.0197 | 0.8557 |
| *Rps11-ps2* | chr11-116382750-116383205 | processed pseudogene | 2.98 | -1.96 | 0.0200 | 0.8557 |
| *Msx1* | chr5-37820485-37824583 | protein coding | 146.19 | -0.59 | 0.0203 | 0.8557 |
| *Pnp2* | chr14-50955992-50964749 | protein coding | 8.48 | -0.98 | 0.0203 | 0.8557 |
| *Pecr* | chr1-72259167-72284314 | protein coding | 120.26 | -0.33 | 0.0204 | 0.8557 |
| *Kcne2* | chr16-92292389-92298129 | protein coding | 71.61 | -2.34 | 0.0206 | 0.8557 |
| *Gm15459* | chr5-5781615-5783555 | processed pseudogene | 151.77 | -0.44 | 0.0207 | 0.8557 |
| *Tgm1* | chr14-55700009-55713926 | protein coding | 9.22 | -2.16 | 0.0208 | 0.8557 |
| *Gm14322* | chr2-177759288-177770472 | protein coding | 76.94 | -0.43 | 0.0209 | 0.8557 |
| *Hspb11* | chr4-107253593-107279938 | protein coding | 168.09 | -0.35 | 0.0210 | 0.8557 |
| *Gm12254* | chr11-58508791-58509261 | processed pseudogene | 9.45 | -1.20 | 0.0210 | 0.8557 |
| *Gm4366* | chr7-116824510-116825851 | processed pseudogene | 50.60 | -0.50 | 0.0211 | 0.8557 |
| *Rdh5* | chr10-128913593-128922888 | protein coding | 71.01 | -0.74 | 0.0214 | 0.8557 |
| *Evi2a* | chr11-79526560-79530609 | protein coding | 405.93 | -0.43 | 0.0215 | 0.8557 |
| *H2-Q7* | chr17-35439155-35443773 | protein coding | 19.43 | -1.04 | 0.0215 | 0.8557 |
| *A2m* | chr6-121635376-121679227 | protein coding | 252.36 | -0.55 | 0.0216 | 0.8557 |
| *Epyc* | chr10-97644068-97682454 | protein coding | 1.37 | -3.01 | 0.0216 | NA |
| *Med31* | chr11-72211724-72215592 | protein coding | 214.12 | -0.30 | 0.0217 | 0.8557 |
| *Pdgfd* | chr9-6168584-6378850 | protein coding | 140.11 | -0.41 | 0.0218 | 0.8557 |
| *Fam151b* | chr13-92449625-92484015 | protein coding | 146.97 | -0.33 | 0.0218 | 0.8557 |
| *Gm8326* | chr1-46414502-46416932 | processed pseudogene | 5.70 | -1.38 | 0.0219 | 0.8557 |
| *Gm39244* | chr8-108526937-108537221 | lncRNA | 8.65 | -1.28 | 0.0219 | 0.8557 |
| *Mt1* | chr8-94179082-94180327 | protein coding | 3127.22 | -0.34 | 0.0223 | 0.8557 |
| *Gm8430* | chr6-122456904-122457374 | processed pseudogene | 14.45 | -1.11 | 0.0223 | 0.8557 |
| *Gm20501* | chr7-141943704-141949539 | lncRNA | 52.56 | -0.41 | 0.0223 | 0.8557 |
| *Igf2bp2* | chr16-22059009-22163299 | protein coding | 15.51 | -0.80 | 0.0224 | 0.8557 |
| *Rpl11* | chr4-136028265-136053428 | protein coding | 1550.73 | -0.34 | 0.0224 | 0.8557 |
| *2310016G11Rik* | chr7-44668233-44678235 | lncRNA | 3.95 | -1.79 | 0.0225 | 0.8557 |
| *Mrpl23-ps1* | chr2-74591330-74591909 | processed pseudogene | 9.73 | -1.04 | 0.0227 | 0.8557 |
| *Amd2* | chr10-35708675-35711892 | protein coding | 14.40 | -0.75 | 0.0227 | 0.8557 |
| *Rassf6* | chr5-90603076-90640657 | protein coding | 13.55 | -1.04 | 0.0229 | 0.8557 |
| *BC028528* | chr3-95883954-95892005 | protein coding | 52.76 | -0.49 | 0.0230 | 0.8557 |
| *Gm20939* | chr17-94864918-94878321 | lncRNA | 40.18 | -0.51 | 0.0232 | 0.8557 |
| *Dpy30* | chr17-74299474-74323944 | protein coding | 391.93 | -0.38 | 0.0235 | 0.8557 |
| *Gm10243* | chr6-48523518-48523850 | processed pseudogene | 2.10 | -2.20 | 0.0236 | NA |
| *Gm10221* | chr5-24581554-24581862 | processed pseudogene | 46.39 | -0.54 | 0.0237 | 0.8557 |
| *Gm13601* | chr2-67491044-67516525 | lncRNA | 2.68 | -2.31 | 0.0239 | 0.8557 |
| *Pigx* | chr16-32084416-32099740 | protein coding | 223.66 | -0.31 | 0.0240 | 0.8557 |
| *Igfbp2* | chr1-72824503-72852474 | protein coding | 586.32 | -0.50 | 0.0240 | 0.8557 |
| *Rpl32* | chr6-115805505-115808747 | protein coding | 2869.99 | -0.37 | 0.0241 | 0.8557 |
| *Itgam* | chr7-128062640-128118491 | protein coding | 179.90 | -0.37 | 0.0242 | 0.8557 |
| *Gng5* | chr3-146499807-146505572 | protein coding | 123.35 | -0.36 | 0.0243 | 0.8557 |
| *Gm12901* | chr4-123258046-123258904 | processed pseudogene | 2.15 | -2.85 | 0.0244 | 0.8557 |
| *Rpl35* | chr2-39001580-39005624 | protein coding | 1224.73 | -0.40 | 0.0246 | 0.8557 |
| *Rpl10a-ps1* | chr1-64993951-64994604 | processed pseudogene | 41.06 | -0.58 | 0.0246 | 0.8557 |
| *Zfp185* | chrX-72987339-73031543 | protein coding | 80.17 | -0.55 | 0.0250 | 0.8557 |
| *Cbln1* | chr8-87468405-87472609 | protein coding | 149.97 | -0.48 | 0.0251 | 0.8557 |
| *Gm7054* | chr13-106475936-106476755 | processed pseudogene | 2.43 | -2.15 | 0.0253 | 0.8557 |
| *Uqcrb* | chr13-66900617-66905378 | protein coding | 1405.55 | -0.35 | 0.0256 | 0.8557 |
| *D830030K20Rik* | chr14-3208488-3234351 | protein coding | 10.68 | -1.00 | 0.0256 | 0.8557 |
| *Mamdc4* | chr2-25563115-25574845 | protein coding | 44.60 | -0.53 | 0.0257 | 0.8557 |
| *Ppp1r17* | chr6-56017497-56032689 | protein coding | 34.76 | -1.05 | 0.0257 | 0.8557 |
| *Psenen-ps* | chr4-40775259-40775564 | processed pseudogene | 16.79 | -0.73 | 0.0258 | 0.8557 |
| *Rps27rt* | chr9-114982366-114982739 | protein coding | 39.32 | -0.68 | 0.0259 | 0.8557 |
| *Zfp931* | chr2-178067695-178078476 | protein coding | 319.13 | -0.35 | 0.0261 | 0.8557 |
| *F5* | chr1-164151838-164220277 | protein coding | 151.86 | -1.62 | 0.0264 | 0.8557 |
| *Lrrc3b* | chr14-15357515-15438987 | protein coding | 144.96 | -0.37 | 0.0264 | 0.8557 |
| *Mesp2* | chr7-79810727-79813439 | protein coding | 10.08 | -1.02 | 0.0265 | 0.8557 |
| *Prdm12* | chr2-31640037-31655795 | protein coding | 8.56 | -1.76 | 0.0265 | 0.8557 |
| *Ndufb1-ps* | chr12-101968090-101977068 | protein coding | 1125.23 | -0.30 | 0.0266 | 0.8557 |
| *2900040C04Rik* | chr14-76251457-76252327 | lncRNA | 33.63 | -1.41 | 0.0267 | 0.8557 |
| *Rpl41* | chr10-128548114-128549305 | protein coding | 3944.18 | -0.34 | 0.0268 | 0.8557 |
| *Hbq1a* | chr11-32300069-32300873 | protein coding | 2.20 | -2.81 | 0.0268 | 0.8557 |
| *Foxp2* | chr6-14901349-15441977 | protein coding | 53.27 | -0.77 | 0.0268 | 0.8557 |
| *Timd4* | chr11-46810800-46844332 | protein coding | 1.65 | -3.29 | 0.0269 | NA |
| *Irak4* | chr15-94543643-94581815 | protein coding | 48.33 | -0.43 | 0.0269 | 0.8557 |
| *Isl1* | chr13-116298281-116309689 | protein coding | 2.36 | -2.89 | 0.0269 | 0.8557 |
| *Slc34a3* | chr2-25228898-25234364 | protein coding | 2.54 | -2.37 | 0.0269 | 0.8557 |
| *Rpl17* | chr18-74998558-75003381 | protein coding | 3088.44 | -0.35 | 0.0270 | 0.8557 |
| *Inmt* | chr6-55170626-55175043 | protein coding | 20.67 | -0.77 | 0.0271 | 0.8557 |
| *Gm11523* | chr11-96862997-96873954 | lncRNA | 4.35 | -1.96 | 0.0274 | 0.8557 |
| *Gm7536* | chr3-24333068-24333511 | processed pseudogene | 27.60 | -0.65 | 0.0278 | 0.8557 |
| *6330562C20Rik* | chr3-95307076-95315127 | lncRNA | 11.11 | -0.85 | 0.0278 | 0.8557 |
| *Gm8203* | chr6-116173737-116174123 | processed pseudogene | 6.94 | -1.06 | 0.0279 | 0.8557 |
| *Gm44126* | chr6-76284416-76284929 | processed pseudogene | 6.06 | -1.27 | 0.0280 | 0.8557 |
| *Cryzl2* | chr1-157458577-157492638 | protein coding | 136.49 | -0.32 | 0.0280 | 0.8557 |
| *Vnn1* | chr10-23894688-23905343 | protein coding | 4.57 | -1.40 | 0.0281 | 0.8557 |
| *Banp* | chr8-121949750-122029258 | protein coding | 715.51 | -0.37 | 0.0285 | 0.8557 |
| *Gm9294* | chr7-42953600-42954388 | processed pseudogene | 4.36 | -1.32 | 0.0289 | 0.8557 |
| *Cox8b* | chr7-140898945-140900446 | protein coding | 11.25 | -1.31 | 0.0290 | 0.8557 |
| *Ndufb11* | chrX-20615326-20617619 | protein coding | 1860.35 | -0.31 | 0.0292 | 0.8557 |
| *Eef1akmt4* | chr16-20611593-20619011 | protein coding | 9.08 | -0.97 | 0.0296 | 0.8557 |
| *Gm5526* | chr1-45857332-45857754 | processed pseudogene | 22.79 | -0.77 | 0.0297 | 0.8557 |
| *Gm16160* | chr3-102203956-102206266 | lncRNA | 1.15 | -2.81 | 0.0298 | NA |
| *Gm38534* | chr11-88253368-88256990 | lncRNA | 5.16 | -1.95 | 0.0298 | 0.8557 |
| *Rps6-ps2* | chr8-88806391-88807137 | processed pseudogene | 3.53 | -1.53 | 0.0300 | 0.8557 |
| *Tpt1-ps6* | chrX-101768535-101769057 | processed pseudogene | 5.00 | -1.38 | 0.0301 | 0.8557 |
| *Gm15423* | chr1-176927999-176932711 | lncRNA | 20.01 | -0.67 | 0.0302 | 0.8557 |
| *Gm42783* | chr3-96724526-96725320 | TEC | 11.61 | -0.96 | 0.0303 | 0.8557 |
| *Polr2j* | chr5-136116631-136122947 | protein coding | 494.23 | -0.34 | 0.0305 | 0.8557 |
| *Vim* | chr2-13573927-13582826 | protein coding | 1282.44 | -0.49 | 0.0306 | 0.8557 |
| *Gm15928* | chr7-3993239-3993518 | processed pseudogene | 1.10 | -2.85 | 0.0306 | NA |
| *Arhgap25* | chr6-87458545-87533259 | protein coding | 84.14 | -0.47 | 0.0307 | 0.8557 |
| *Hint2* | chr4-43654227-43656466 | protein coding | 280.69 | -0.30 | 0.0310 | 0.8557 |
| *Rpl14-ps1* | chr7-45324965-45325617 | processed pseudogene | 176.00 | -0.32 | 0.0311 | 0.8557 |
| *Hsd17b10* | chrX-152001845-152004442 | protein coding | 583.52 | -0.30 | 0.0312 | 0.8557 |
| *Zfp975* | chr7-42660105-42692742 | protein coding | 103.28 | -0.37 | 0.0312 | 0.8557 |
| *Lmx1a* | chr1-167689237-167848741 | protein coding | 10.32 | -1.42 | 0.0313 | 0.8557 |
| *Rpl7a-ps7* | chr3-129461972-129462747 | processed pseudogene | 6.90 | -1.03 | 0.0315 | 0.8557 |
| *Gm4734* | chr9-109970433-109971209 | processed pseudogene | 6.34 | -1.28 | 0.0315 | 0.8557 |
| *Gm12297* | chr11-66563607-66564585 | processed pseudogene | 15.07 | -0.75 | 0.0316 | 0.8557 |
| *Gm5138* | chr6-83874379-83875380 | processed pseudogene | 3.27 | -1.69 | 0.0318 | 0.8557 |
| *Dab2* | chr15-6299788-6440712 | protein coding | 232.33 | -0.32 | 0.0319 | 0.8557 |
| *Gm3200* | chr7-100760805-100761806 | processed pseudogene | 5.78 | -1.27 | 0.0320 | 0.8557 |
| *Rpl34-ps2* | chr13-116153781-116154134 | processed pseudogene | 3.19 | -1.64 | 0.0321 | 0.8557 |
| *Gm8864* | chrX-88572051-88573060 | processed pseudogene | 3.55 | -1.63 | 0.0321 | 0.8557 |
| *Gm11488* | chr4-73497615-73498477 | processed pseudogene | 1.48 | -3.10 | 0.0323 | NA |
| *Cldn3* | chr5-134986214-134987472 | protein coding | 4.06 | -1.98 | 0.0325 | 0.8557 |
| *Mrpl15* | chr1-4773206-4785739 | protein coding | 705.60 | -0.30 | 0.0327 | 0.8557 |
| *Spint1* | chr2-119237362-119249527 | protein coding | 40.26 | -0.52 | 0.0327 | 0.8557 |
| *Lrrc69* | chr4-14623620-14796060 | protein coding | 1.25 | -2.69 | 0.0329 | NA |
| *Aqp6* | chr15-99601400-99605477 | protein coding | 3.97 | -1.68 | 0.0330 | 0.8557 |
| *Gm10076* | chr14-105681828-105682211 | lncRNA | 20.30 | -0.78 | 0.0330 | 0.8557 |
| *Gm33467* | chr12-108695486-108696446 | lncRNA | 2.03 | -2.14 | 0.0331 | NA |
| *Igfbpl1* | chr4-45809468-45826923 | protein coding | 83.40 | -0.96 | 0.0331 | 0.8557 |
| *Atp5j2* | chr5-145183698-145192062 | protein coding | 2104.53 | -0.33 | 0.0332 | 0.8557 |
| *Uqcrq* | chr11-53427922-53430831 | protein coding | 2128.27 | -0.37 | 0.0333 | 0.8557 |
| *Gm18086* | chr18-42186175-42187772 | processed pseudogene | 1.81 | -2.59 | 0.0333 | NA |
| *Ecrg4* | chr1-43730602-43742578 | protein coding | 149.75 | -1.54 | 0.0334 | 0.8557 |
| *Gm9521* | chr7-18229777-18231292 | processed pseudogene | 1.54 | -3.45 | 0.0334 | NA |
| *Gm17087* | chr17-8565852-8566950 | protein coding | 1.03 | -3.14 | 0.0335 | NA |
| *A730009L09Rik* | chr16-84515121-84523888 | protein coding | 10.62 | -0.84 | 0.0337 | 0.8557 |
| *Kif20b* | chr19-34922361-34975745 | protein coding | 19.37 | -0.72 | 0.0339 | 0.8557 |
| *Ano2* | chr6-125690419-126040126 | protein coding | 42.31 | -0.66 | 0.0339 | 0.8557 |
| *Pcp2* | chr8-3623371-3625545 | protein coding | 47.85 | -1.42 | 0.0341 | 0.8557 |
| *Gm21596* | chr2-113919247-113920562 | processed pseudogene | 2.03 | -2.05 | 0.0342 | NA |
| *Dao* | chr5-114003703-114025682 | protein coding | 11.33 | -1.09 | 0.0343 | 0.8557 |
| *Prkg2* | chr5-98929773-99037351 | protein coding | 67.50 | -0.87 | 0.0343 | 0.8557 |
| *Ighg2b* | chr12-113302965-113307933 | IG C gene | 3.67 | -2.98 | 0.0344 | 0.8557 |
| *Polr2k* | chr15-36174010-36177010 | protein coding | 175.10 | -0.34 | 0.0346 | 0.8557 |
| *Serpinb1b* | chr13-33078575-33094380 | protein coding | 18.12 | -0.88 | 0.0346 | 0.8557 |
| *Stap1* | chr5-86071746-86106125 | protein coding | 1.26 | -2.87 | 0.0346 | NA |
| *Gm48339* | chr14-20057852-20058297 | processed pseudogene | 1.66 | -2.69 | 0.0347 | NA |
| *Gm6988* | chr12-110315990-110316364 | processed pseudogene | 17.15 | -0.84 | 0.0347 | 0.8557 |
| *9430025C20Rik* | chr4-3806635-3808751 | pseudogene | 16.24 | -0.85 | 0.0348 | 0.8557 |
| *Igf2* | chr7-142650766-142666816 | protein coding | 21.42 | -0.85 | 0.0348 | 0.8557 |
| *Rps16* | chr7-28350652-28353155 | protein coding | 1317.45 | -0.38 | 0.0350 | 0.8557 |
| *Tafa4* | chr6-96831203-97060413 | protein coding | 21.50 | -1.32 | 0.0352 | 0.8557 |
| *Fhl5* | chr4-25199908-25242876 | protein coding | 2.30 | -2.65 | 0.0352 | 0.8557 |
| *Mdfic* | chr6-15720661-15802169 | protein coding | 88.81 | -0.62 | 0.0353 | 0.8557 |
| *Cenpa* | chr5-30666777-30674830 | protein coding | 45.06 | -0.46 | 0.0354 | 0.8557 |
| *Khdrbs2* | chr1-32172714-32658568 | protein coding | 194.70 | -0.36 | 0.0354 | 0.8557 |
| *H4c11* | chr13-21735064-21735837 | protein coding | 8.35 | -0.91 | 0.0354 | 0.8557 |
| *Sln* | chr9-53850164-53854560 | protein coding | 12.57 | -1.21 | 0.0355 | 0.8557 |
| *Snhg10* | chr12-105030617-105032279 | lncRNA | 49.54 | -0.46 | 0.0356 | 0.8557 |
| *Lmx1b* | chr2-33560965-33640511 | protein coding | 2.96 | -2.30 | 0.0357 | 0.8557 |
| *Gpx8* | chr13-113042753-113046410 | protein coding | 107.82 | -0.50 | 0.0357 | 0.8557 |
| *Rpl37a* | chr1-72711290-72713813 | protein coding | 1685.01 | -0.35 | 0.0357 | 0.8557 |
| *Gm15753* | chr5-137720354-137721740 | unprocessed pseudogene | 8.72 | -1.02 | 0.0358 | 0.8557 |
| *Zic5* | chr14-122456795-122465677 | protein coding | 97.48 | -0.49 | 0.0359 | 0.8557 |
| *Serpinb1a* | chr13-32842092-32851185 | protein coding | 226.45 | -0.49 | 0.0360 | 0.8557 |
| *Mael* | chr1-166201201-166238747 | protein coding | 3.54 | -2.26 | 0.0361 | 0.8557 |
| *Rpl38-ps1* | chr4-138425788-138426002 | processed pseudogene | 5.09 | -1.25 | 0.0362 | 0.8557 |
| *Aim2* | chr1-173350879-173466040 | protein coding | 140.92 | -0.33 | 0.0363 | 0.8557 |
| *Oit3* | chr10-59422958-59441778 | protein coding | 2.66 | -1.98 | 0.0364 | 0.8557 |
| *Pinx1* | chr14-63860364-63919859 | protein coding | 112.24 | -0.36 | 0.0365 | 0.8557 |
| *Svbp* | chr4-119195306-119201298 | protein coding | 173.84 | -0.37 | 0.0366 | 0.8557 |
| *Gm31645* | chr17-74519665-74528094 | lncRNA | 6.99 | -1.03 | 0.0368 | 0.8557 |
| *Lbx2* | chr6-83086367-83088243 | protein coding | 3.59 | -1.46 | 0.0368 | 0.8557 |
| *E230013L22Rik* | chr8-11477929-11480241 | lncRNA | 3.39 | -2.32 | 0.0369 | 0.8557 |
| *Batf* | chr12-85686669-85709087 | protein coding | 3.02 | -2.04 | 0.0369 | 0.8557 |
| *Gm12263* | chr11-59474284-59475765 | processed pseudogene | 3.16 | -1.69 | 0.0370 | 0.8557 |
| *Cd302* | chr2-60251993-60284488 | protein coding | 264.98 | -0.33 | 0.0371 | 0.8557 |
| *Gm4945* | chr17-47042587-47043039 | processed pseudogene | 0.89 | -2.69 | 0.0373 | NA |
| *Gm40117* | chr3-104994032-105004005 | lncRNA | 6.70 | -1.09 | 0.0373 | 0.8557 |
| *Gm26725* | chr5-66151212-66174945 | lncRNA | 1.20 | -3.08 | 0.0375 | NA |
| *A930005H10Rik* | chr3-115881581-115888130 | lncRNA | 173.20 | -0.38 | 0.0375 | 0.8557 |
| *Tbca* | chr13-94788910-94842922 | protein coding | 707.58 | -0.35 | 0.0376 | 0.8557 |
| *Gm8773* | chr5-5573799-5576203 | protein coding | 1.66 | -2.96 | 0.0377 | NA |
| *Mir99ahg* | chr16-77236317-77773427 | lncRNA | 159.94 | -0.35 | 0.0378 | 0.8557 |
| *Smim27* | chr4-40269579-40270940 | protein coding | 33.57 | -0.48 | 0.0380 | 0.8557 |
| *Hacl1* | chr14-31598730-31641286 | protein coding | 5.80 | -1.16 | 0.0380 | 0.8557 |
| *Gm4841* | chr18-60268305-60273272 | protein coding | 2.37 | -2.28 | 0.0381 | 0.8557 |
| *Gm26861* | chr13-10357595-10797502 | lncRNA | 1.51 | -2.81 | 0.0382 | NA |
| *Rpsa-ps12* | chr4-149142497-149143388 | processed pseudogene | 3.31 | -1.86 | 0.0385 | 0.8557 |
| *Gm13340* | chr2-22588103-22589647 | unprocessed pseudogene | 86.92 | -0.59 | 0.0386 | 0.8557 |
| *Ap1s2* | chrX-163909017-163933666 | protein coding | 956.18 | -0.33 | 0.0386 | 0.8557 |
| *Dleu2* | chr14-61602839-61682373 | lncRNA | 193.08 | -0.32 | 0.0388 | 0.8557 |
| *Gm5711* | chr3-121380259-121380679 | processed pseudogene | 3.82 | -1.77 | 0.0388 | 0.8557 |
| *Nt5dc1* | chr10-34288288-34418552 | protein coding | 62.66 | -0.37 | 0.0388 | 0.8557 |
| *Gm8121* | chr5-57608569-57609067 | processed pseudogene | 37.76 | -0.56 | 0.0389 | 0.8557 |
| *Gm8186* | chr17-26098991-26099221 | processed pseudogene | 11.51 | -0.86 | 0.0390 | 0.8557 |
| *Gm44250* | chr6-5216258-5220486 | TEC | 88.49 | -0.70 | 0.0393 | 0.8557 |
| *Rom1* | chr19-8927391-8929356 | protein coding | 99.73 | -0.34 | 0.0393 | 0.8557 |
| *Gm6392* | chr15-60468124-60469477 | processed pseudogene | 4.03 | -1.58 | 0.0398 | 0.8584 |
| *Knstrn* | chr2-118814003-118853957 | protein coding | 33.59 | -0.44 | 0.0398 | 0.8584 |
| *4933429O19Rik* | chr14-48878586-48887112 | lncRNA | 4.04 | -1.85 | 0.0399 | 0.8584 |
| *Mpp7* | chr18-7347959-7626866 | protein coding | 95.35 | -0.42 | 0.0401 | 0.8589 |
| *Gm37983* | chr9-72315131-72317136 | TEC | 6.49 | -1.35 | 0.0403 | 0.8595 |
| *Gm11993* | chr11-9149778-9150597 | processed pseudogene | 3.57 | -1.36 | 0.0407 | 0.8595 |
| *Tcf7l2* | chr19-55741820-55933654 | protein coding | 249.83 | -0.95 | 0.0408 | 0.8595 |
| *Pax7* | chr4-139737062-139833528 | protein coding | 12.79 | -1.68 | 0.0409 | 0.8595 |
| *Rps25* | chr9-44407139-44410427 | protein coding | 2867.25 | -0.32 | 0.0409 | 0.8595 |
| *Dynlrb1* | chr2-155236533-155250277 | protein coding | 2579.97 | -0.34 | 0.0411 | 0.8595 |
| *Gm9512* | chr13-15118632-15119128 | processed pseudogene | 3.76 | -1.42 | 0.0412 | 0.8595 |
| *Gm12315* | chr11-70623027-70625561 | lncRNA | 1.90 | -2.80 | 0.0415 | NA |
| *Grcc10* | chr6-124739183-124741374 | protein coding | 98.42 | -0.34 | 0.0416 | 0.8627 |
| *Ms4a7* | chr19-11321039-11336146 | protein coding | 10.34 | -1.02 | 0.0419 | 0.8658 |
| *Bloc1s1* | chr10-128917882-128924035 | protein coding | 22.32 | -0.59 | 0.0422 | 0.8659 |
| *Pdpn* | chr4-143267431-143299564 | protein coding | 278.25 | -0.30 | 0.0423 | 0.8659 |
| *Pou4f1* | chr14-104461676-104467999 | protein coding | 15.18 | -1.54 | 0.0424 | 0.8659 |
| *Snrpert* | chr2-131322088-131322366 | processed pseudogene | 2.12 | -1.97 | 0.0426 | NA |
| *Clic6* | chr16-92485736-92541243 | protein coding | 266.63 | -1.37 | 0.0427 | 0.8659 |
| *Gm8292* | chr1-55335401-55335796 | processed pseudogene | 18.61 | -0.65 | 0.0430 | 0.8659 |
| *Zfp982* | chr4-147492417-147513486 | protein coding | 13.08 | -0.76 | 0.0433 | 0.8659 |
| *Gm10240* | chr15-68309819-68310301 | processed pseudogene | 2.17 | -2.45 | 0.0434 | 0.8659 |
| *Ntf5* | chr7-45413695-45417179 | protein coding | 4.78 | -1.19 | 0.0436 | 0.8659 |
| *Med29* | chr7-28386146-28392708 | protein coding | 159.68 | -0.32 | 0.0438 | 0.8659 |
| *Gm4977* | chr9-17732670-17733931 | processed pseudogene | 2.31 | -1.86 | 0.0438 | 0.8659 |
| *Gm6210* | chr6-75296118-75298059 | processed pseudogene | 14.49 | -0.88 | 0.0438 | 0.8659 |
| *Gm14586* | chrX-52772342-52772749 | processed pseudogene | 32.64 | -0.59 | 0.0439 | 0.8659 |
| *Vamp8* | chr6-72385223-72390703 | protein coding | 200.27 | -0.40 | 0.0440 | 0.8659 |
| *Gm11966* | chr11-5818497-5818583 | processed pseudogene | 9.88 | -1.01 | 0.0442 | 0.8659 |
| *Gm28523* | chr6-141246920-141250009 | lncRNA | 10.31 | -0.80 | 0.0443 | 0.8659 |
| *Mapkapk3* | chr9-107254927-107289877 | protein coding | 53.20 | -0.44 | 0.0444 | 0.8659 |
| *Rilp* | chr11-75510094-75513168 | protein coding | 36.59 | -0.47 | 0.0444 | 0.8659 |
| *Rad51c* | chr11-87376645-87404954 | protein coding | 82.75 | -0.34 | 0.0446 | 0.8659 |
| *Jchain* | chr5-88519809-88527891 | protein coding | 18.80 | -0.91 | 0.0448 | 0.8659 |
| *Nop10* | chr2-112261926-112263269 | protein coding | 566.42 | -0.32 | 0.0448 | 0.8659 |
| *Xkr5* | chr8-18932729-18950975 | protein coding | 2.15 | -2.18 | 0.0448 | 0.8659 |
| *Inhba* | chr13-16011851-16031621 | protein coding | 275.92 | -0.33 | 0.0448 | 0.8659 |
| *Rps12* | chr10-23785183-23787275 | protein coding | 2063.31 | -0.32 | 0.0448 | 0.8659 |
| *H19* | chr7-142575529-142578143 | lncRNA | 4.46 | -1.43 | 0.0449 | 0.8659 |
| *Gm12922* | chr4-123709219-123710027 | processed pseudogene | 0.77 | -2.93 | 0.0450 | NA |
| *H2-Q1* | chr17-35320405-35325099 | protein coding | 5.35 | -2.03 | 0.0451 | 0.8671 |
| *Tubb6* | chr18-67390717-67402749 | protein coding | 20.93 | -0.71 | 0.0454 | 0.8671 |
| *Gm4149* | chr13-75644896-75645171 | processed pseudogene | 22.94 | -0.65 | 0.0454 | 0.8671 |
| *Cd63* | chr10-128900989-128912822 | protein coding | 981.23 | -0.31 | 0.0454 | 0.8671 |
| *Akr1b8* | chr6-34354119-34368463 | protein coding | 14.11 | -0.66 | 0.0457 | 0.8675 |
| *Gm14046* | chr2-129749901-129750409 | processed pseudogene | 5.22 | -1.41 | 0.0458 | 0.8675 |
| *Gm47154* | chr9-14807774-14808040 | processed pseudogene | 0.83 | -2.99 | 0.0462 | NA |
| *Kcnj13* | chr1-87386363-87394729 | protein coding | 74.08 | -1.75 | 0.0465 | 0.8686 |
| *Gm8494* | chr5-146574888-146576120 | processed pseudogene | 3.31 | -1.86 | 0.0468 | 0.8686 |
| *Lgals3* | chr14-47367751-47386160 | protein coding | 27.03 | -0.97 | 0.0468 | 0.8686 |
| *Mt3* | chr8-94152607-94154146 | protein coding | 6389.69 | -0.32 | 0.0469 | 0.8686 |
| *Gm26910* | chr18-67773712-67774857 | lncRNA | 30.39 | -0.57 | 0.0470 | 0.8686 |
| *Cnpy1* | chr5-28200819-28245806 | protein coding | 52.74 | -0.85 | 0.0470 | 0.8686 |
| *Gm13715* | chr2-85233675-85243678 | lncRNA | 1.67 | -2.42 | 0.0474 | NA |
| *Smim18* | chr8-33742112-33747770 | protein coding | 119.29 | -0.35 | 0.0474 | 0.8686 |
| *Gm47484* | chr12-84313854-84317076 | lncRNA | 48.38 | -0.41 | 0.0474 | 0.8686 |
| *Vpreb1* | chr16-16868403-16870843 | protein coding | 3.86 | -1.54 | 0.0475 | 0.8686 |
| *Gm14010* | chr2-128423654-128424565 | lncRNA | 0.97 | -2.89 | 0.0475 | NA |
| *Myl12a* | chr17-70993656-71002878 | protein coding | 158.60 | -0.31 | 0.0475 | 0.8694 |
| *Snhg7os* | chr2-26640372-26645944 | lncRNA | 1.05 | -3.09 | 0.0480 | NA |
| *Zic2* | chr14-122475435-122479852 | protein coding | 331.63 | -0.38 | 0.0481 | 0.8702 |
| *En1* | chr1-120602418-120607992 | protein coding | 1.20 | -2.83 | 0.0484 | NA |
| *Glycam1* | chr15-103562759-103565081 | protein coding | 11.80 | -1.15 | 0.0486 | 0.8702 |
| *Rpl28-ps1* | chr1-128038569-128038982 | processed pseudogene | 24.94 | -0.64 | 0.0486 | 0.8702 |
| *Tmem267* | chr13-119488039-119611059 | protein coding | 380.51 | -0.36 | 0.0487 | 0.8702 |
| *S100a8* | chr3-90668978-90670035 | protein coding | 3.16 | -1.54 | 0.0487 | 0.8702 |
| *Pifo* | chr3-105996957-106014646 | protein coding | 19.21 | -1.02 | 0.0489 | 0.8702 |
| *Lpar6* | chr14-73237895-73243294 | protein coding | 134.48 | -0.33 | 0.0490 | 0.8702 |
| *Eif4a-ps4* | chr1-60703932-60705149 | processed pseudogene | 52.45 | -0.45 | 0.0495 | 0.8702 |
| *Rps12-ps10* | chr2-122219993-122220391 | processed pseudogene | 2.66 | -1.87 | 0.0497 | 0.8702 |

**Supplementary Table 6C. Upregulated genes in Pool 2 vs Control mice (Log2 Fold Change ≥ 0.3, p-value < 0.05)**.

| Gene symbol | Chromosomal location | Biotype | baseMean | log2FC | pvalue | padj |
| --- | --- | --- | --- | --- | --- | --- |
| *Ehd4* | chr2-120089175-120154606 | protein coding | 187.18 | 0.53 | 2.56E-06 | 0.0131 |
| *Gm23935* | chr16-11144125-11144181 | miRNA | 2259.45 | 2.82 | 6.28E-06 | 0.0257 |
| *Gm15564* | chr16-35966752-35983230 | lncRNA | 57.85 | 1.86 | 7.73E-06 | 0.0264 |
| *Sgpp2* | chr1-78310345-78420289 | protein coding | 195.36 | 0.94 | 1.08E-05 | 0.0278 |
| *Mir6236* | chr9-110281287-110281409 | miRNA | 112.80 | 2.21 | 1.12E-05 | 0.0278 |
| *Patj* | chr4-98395785-98719603 | protein coding | 208.63 | 1.29 | 1.22E-05 | 0.0278 |
| *Adra1b* | chr11-43774606-43901210 | protein coding | 85.34 | 1.31 | 1.37E-05 | 0.0280 |
| *Gpx3* | chr11-54902453-54910377 | protein coding | 60.41 | 1.46 | 2.10E-05 | 0.0385 |
| *Gm42418* | chr17-39846958-39848788 | lncRNA | 89954.62 | 2.53 | 2.26E-05 | 0.0385 |
| *Rab37* | chr11-115091431-115162236 | protein coding | 109.36 | 1.48 | 4.63E-05 | 0.0691 |
| *Rgs16* | chr1-153740349-153745468 | protein coding | 210.16 | 1.26 | 4.73E-05 | 0.0691 |
| *Lars2* | chr9-123366927-123462666 | protein coding | 10078.79 | 2.21 | 5.35E-05 | 0.0730 |
| *Gm26917* | chr17-39843013-39846341 | lncRNA | 624.31 | 2.39 | 6.60E-05 | 0.0812 |
| *Lamc3* | chr2-31887291-31946539 | protein coding | 150.53 | 0.72 | 6.74E-05 | 0.0812 |
| *Chrna4* | chr2-181018380-181043546 | protein coding | 443.06 | 0.82 | 8.03E-05 | 0.0866 |
| *Gm24270* | chr9-56223715-56223771 | miRNA | 118.27 | 2.71 | 8.04E-05 | 0.0866 |
| *Tmem132c* | chr5-127241808-127565793 | protein coding | 93.56 | 0.86 | 8.92E-05 | 0.0876 |
| *Adamts15* | chr9-30899155-30922452 | protein coding | 97.33 | 0.91 | 8.99E-05 | 0.0876 |
| *Rasgef1b* | chr5-99217426-99729065 | protein coding | 233.80 | 0.78 | 9.47E-05 | 0.0881 |
| *Aspg* | chr12-112106679-112127559 | protein coding | 100.07 | 0.77 | 0.0001 | 0.0938 |
| *Cited1* | chrX-102247381-102252181 | protein coding | 19.30 | 1.32 | 0.0001 | 0.0938 |
| *Zdhhc22* | chr12-86980763-86990430 | protein coding | 314.57 | 1.06 | 0.0001 | 0.0938 |
| *Hcn4* | chr9-58823412-58863175 | protein coding | 64.97 | 1.22 | 0.0001 | 0.0956 |
| *Gm43398* | chr5-143395503-143398244 | TEC | 73.00 | 1.46 | 0.0001 | 0.0965 |
| *Opn3* | chr1-175662421-175692776 | protein coding | 16.64 | 1.45 | 0.0001 | 0.0996 |
| *Itih5* | chr2-10153571-10256529 | protein coding | 797.03 | 0.40 | 0.0001 | 0.0996 |
| *Plxdc1* | chr11-97923238-97986444 | protein coding | 221.08 | 0.77 | 0.0002 | 0.1102 |
| *Cd74* | chr18-60803848-60812652 | protein coding | 96.68 | 1.36 | 0.0002 | 0.1177 |
| *Ret* | chr6-118151745-118197718 | protein coding | 88.48 | 1.21 | 0.0002 | 0.1277 |
| *n-R5-8s1* | chr18-73533403-73533550 | rRNA | 29.34 | 2.69 | 0.0002 | 0.1305 |
| *Hspg2* | chr4-137468769-137570630 | protein coding | 240.83 | 0.73 | 0.0002 | 0.1305 |
| *Lef1* | chr3-131110471-131224356 | protein coding | 129.88 | 0.98 | 0.0002 | 0.1305 |
| *A2m* | chr6-121635376-121679227 | protein coding | 252.36 | 0.90 | 0.0003 | 0.1305 |
| *Eps8l2* | chr7-141338880-141363020 | protein coding | 121.99 | 0.85 | 0.0003 | 0.1305 |
| *Col16a1* | chr4-130047840-130099283 | protein coding | 332.10 | 0.54 | 0.0003 | 0.1305 |
| *Synpo2* | chr3-123076519-123236149 | protein coding | 249.38 | 1.40 | 0.0003 | 0.1305 |
| *Serpinf1* | chr11-75409769-75422701 | protein coding | 290.05 | 0.66 | 0.0003 | 0.1305 |
| *Gm24187* | chr13-9834469-9834525 | miRNA | 822.87 | 2.46 | 0.0003 | 0.1305 |
| *Scarna2* | chr3-108554338-108554751 | scaRNA | 3.74 | 4.09 | 0.0003 | 0.1320 |
| *Mrc2* | chr11-105292643-105351139 | protein coding | 162.14 | 0.59 | 0.0003 | 0.1320 |
| *Epha8* | chr4-136929419-136956816 | protein coding | 92.72 | 0.90 | 0.0003 | 0.1457 |
| *Gm22009* | chr3-69085105-69085447 | scaRNA | 13.07 | 2.02 | 0.0003 | 0.1459 |
| *Vash2* | chr1-190947646-190979296 | protein coding | 55.77 | 0.84 | 0.0003 | 0.1475 |
| *Cnn1* | chr9-22099216-22109630 | protein coding | 10.45 | 1.45 | 0.0004 | 0.1495 |
| *Gm24245* | chr4-139338304-139338360 | miRNA | 12.23 | 2.62 | 0.0004 | 0.1519 |
| *Olfml2a* | chr2-38931978-38963753 | protein coding | 68.20 | 1.01 | 0.0004 | 0.1659 |
| *Piezo1* | chr8-122481698-122551329 | protein coding | 127.48 | 0.58 | 0.0004 | 0.1701 |
| *Pabpc1l* | chr2-164025450-164050538 | protein coding | 12.75 | 1.31 | 0.0004 | 0.1701 |
| *Itga3* | chr11-95044474-95076801 | protein coding | 323.30 | 0.48 | 0.0005 | 0.1716 |
| *Heyl* | chr4-123233556-123249875 | protein coding | 79.61 | 0.69 | 0.0005 | 0.1850 |
| *Gpr179* | chr11-97332109-97352077 | protein coding | 15.73 | 1.54 | 0.0005 | 0.1850 |
| *Rn7sk* | chr9-78175303-78175633 | miscRNA | 582.43 | 2.77 | 0.0005 | 0.1871 |
| *Tafa4* | chr6-96831203-97060413 | protein coding | 21.50 | 2.08 | 0.0006 | 0.1917 |
| *Gimap4* | chr6-48684549-48692060 | protein coding | 10.12 | 1.62 | 0.0006 | 0.1917 |
| *Smoc1* | chr12-81026808-81186414 | protein coding | 272.20 | 0.41 | 0.0006 | 0.1943 |
| *Cyp1b1* | chr17-79701537-79715061 | protein coding | 116.48 | 0.80 | 0.0006 | 0.1951 |
| *Lamb2* | chr9-108479736-108490530 | protein coding | 749.23 | 0.51 | 0.0006 | 0.1972 |
| *Gjc1* | chr11-102799579-102819700 | protein coding | 65.82 | 0.79 | 0.0006 | 0.1972 |
| *Slc2a4* | chr11-69942539-69948188 | protein coding | 22.96 | 1.33 | 0.0006 | 0.1972 |
| *Cytl1* | chr5-37735519-37739820 | protein coding | 10.87 | 2.10 | 0.0007 | 0.2023 |
| *Mrvi1* | chr7-110868266-110982461 | protein coding | 67.48 | 1.22 | 0.0007 | 0.2093 |
| *Ltbp2* | chr12-84783212-84876532 | protein coding | 14.45 | 1.27 | 0.0007 | 0.2093 |
| *H2-Aa* | chr17-34282744-34287823 | protein coding | 50.15 | 1.28 | 0.0007 | 0.2093 |
| *Fbln5* | chr12-101746565-101819055 | protein coding | 78.56 | 0.92 | 0.0008 | 0.2177 |
| *Nid1* | chr13-13437551-13512269 | protein coding | 176.66 | 0.53 | 0.0008 | 0.2177 |
| *9530082P21Rik* | chr17-23743234-23754065 | lncRNA | 46.38 | 0.76 | 0.0008 | 0.2240 |
| *Cd93* | chr2-148436640-148443563 | protein coding | 167.06 | 0.59 | 0.0009 | 0.2327 |
| *Tgfbr2* | chr9-116084293-116175360 | protein coding | 473.43 | 0.39 | 0.0009 | 0.2407 |
| *Abhd12b* | chr12-70154142-70183887 | protein coding | 12.26 | 2.31 | 0.0009 | 0.2412 |
| *Uba7* | chr9-107975505-107984060 | protein coding | 104.17 | 0.62 | 0.0010 | 0.2706 |
| *Scube2* | chr7-109798676-109865679 | protein coding | 37.00 | 1.08 | 0.0010 | 0.2706 |
| *Syt9* | chr7-107370728-107548656 | protein coding | 151.83 | 1.00 | 0.0011 | 0.2725 |
| *Rasa4* | chr5-136083916-136111860 | protein coding | 124.97 | 0.62 | 0.0011 | 0.2750 |
| *Pip5kl1* | chr2-32574797-32583782 | protein coding | 54.73 | 0.72 | 0.0011 | 0.2774 |
| *Sema4g* | chr19-44989101-45003397 | protein coding | 498.66 | 0.45 | 0.0011 | 0.2805 |
| *Mir6240* | chr5-114851948-114852064 | miRNA | 2.65 | 3.63 | 0.0012 | 0.2873 |
| *Gm37795* | chr4-150068539-150070997 | TEC | 20.39 | 1.08 | 0.0012 | 0.2873 |
| *Mrc1* | chr2-14229392-14332057 | protein coding | 59.25 | 0.84 | 0.0012 | 0.2873 |
| *Slc22a2* | chr17-12584132-12628488 | protein coding | 8.19 | 3.19 | 0.0013 | 0.2998 |
| *Slc36a2* | chr11-55158470-55185077 | protein coding | 16.83 | 1.58 | 0.0013 | 0.3007 |
| *Micall2* | chr5-139706696-139736336 | protein coding | 54.92 | 0.61 | 0.0013 | 0.3007 |
| *Kmo* | chr1-175620381-175662116 | protein coding | 3.77 | 2.64 | 0.0014 | 0.3104 |
| *Hsbp1l1* | chr18-80229758-80247102 | protein coding | 12.15 | 1.13 | 0.0014 | 0.3104 |
| *Nexn* | chr3-152236982-152266350 | protein coding | 41.49 | 1.19 | 0.0014 | 0.3104 |
| *Lama5* | chr2-180176373-180225859 | protein coding | 334.93 | 0.54 | 0.0014 | 0.3104 |
| *Prkch* | chr12-73584796-73778185 | protein coding | 89.29 | 0.74 | 0.0014 | 0.3116 |
| *Plekhg2* | chr7-28359604-28372599 | protein coding | 134.39 | 0.42 | 0.0015 | 0.3133 |
| *Wif1* | chr10-121033960-121100650 | protein coding | 33.08 | 0.99 | 0.0015 | 0.3163 |
| *Adgrg6* | chr10-14402583-14545659 | protein coding | 37.13 | 0.76 | 0.0015 | 0.3163 |
| *Nkg7* | chr7-43437073-43438249 | protein coding | 2.36 | 3.69 | 0.0015 | 0.3203 |
| *Rgs4* | chr1-169741477-169747642 | protein coding | 2795.22 | 0.31 | 0.0016 | 0.3232 |
| *Ltbp3* | chr19-5740904-5758532 | protein coding | 651.05 | 0.36 | 0.0016 | 0.3232 |
| *Cntn6* | chr6-104492790-104863406 | protein coding | 142.78 | 0.74 | 0.0016 | 0.3232 |
| *Grid2ip* | chr5-143357338-143392152 | protein coding | 70.21 | 1.29 | 0.0017 | 0.3232 |
| *Amotl1* | chr9-14541966-14645056 | protein coding | 708.15 | 0.79 | 0.0017 | 0.3232 |
| *Akap12* | chr10-4266380-4359470 | protein coding | 436.43 | 0.52 | 0.0017 | 0.3232 |
| *Zfp41* | chr15-75616679-75629827 | protein coding | 177.42 | 0.39 | 0.0017 | 0.3242 |
| *Btc* | chr5-91357261-91402994 | protein coding | 11.14 | 1.27 | 0.0017 | 0.3274 |
| *CT010467.1* | chr17-39846353-39848827 | rRNA | 14.12 | 2.54 | 0.0018 | 0.3305 |
| *Vangl1* | chr3-102153583-102204693 | protein coding | 105.85 | 0.73 | 0.0018 | 0.3305 |
| *Fignl2* | chr15-101050194-101078567 | protein coding | 31.58 | 0.79 | 0.0018 | 0.3311 |
| *Itih3* | chr14-30908572-30923760 | protein coding | 537.78 | 0.35 | 0.0018 | 0.3311 |
| *Adra2b* | chr2-127363208-127367221 | protein coding | 8.70 | 1.65 | 0.0019 | 0.3312 |
| *Serping1* | chr2-84765387-84775444 | protein coding | 209.73 | 0.87 | 0.0019 | 0.3357 |
| *Tnnt1* | chr7-4504570-4516382 | protein coding | 99.18 | 1.94 | 0.0019 | 0.3357 |
| *C330011M18Rik* | chr8-84065236-84067287 | lncRNA | 59.21 | 0.57 | 0.0019 | 0.3357 |
| *Mab21l1* | chr3-55782510-55785001 | protein coding | 28.25 | 1.11 | 0.0020 | 0.3384 |
| *Gstm2* | chr3-107981702-107986453 | protein coding | 34.35 | 0.82 | 0.0020 | 0.3384 |
| *Slc17a6* | chr7-51622006-51671119 | protein coding | 416.01 | 1.75 | 0.0020 | 0.3384 |
| *Gm24601* | chr6-94826781-94826923 | rRNA | 2.48 | 3.98 | 0.0020 | 0.3384 |
| *Stab1* | chr14-31139013-31168641 | protein coding | 392.62 | 0.39 | 0.0020 | 0.3402 |
| *Adcy7* | chr8-88272403-88329962 | protein coding | 120.86 | 0.53 | 0.0021 | 0.3430 |
| *Atp2a1* | chr7-126445858-126463108 | protein coding | 13.05 | 1.92 | 0.0021 | 0.3460 |
| *Gm43319* | chr5-48799010-48801279 | TEC | 1.24 | 4.38 | 0.0022 | NA |
| *Parp3* | chr9-106470322-106476949 | protein coding | 105.21 | 0.45 | 0.0022 | 0.3473 |
| *F13a1* | chr13-36867178-37050244 | protein coding | 31.62 | 0.95 | 0.0022 | 0.3473 |
| *Sdc1* | chr12-8771323-8793715 | protein coding | 17.44 | 1.09 | 0.0022 | 0.3473 |
| *Baiap3* | chr17-25242659-25256364 | protein coding | 146.99 | 1.30 | 0.0022 | 0.3473 |
| *Siglec1* | chr2-131069220-131086765 | protein coding | 8.95 | 1.88 | 0.0023 | 0.3531 |
| *Gm24265* | chr5-115489459-115489599 | snRNA | 12.20 | 2.53 | 0.0023 | 0.3533 |
| *Fgl2* | chr5-21372642-21378374 | protein coding | 43.64 | 0.97 | 0.0024 | 0.3574 |
| *Angel1* | chr12-86700502-86726460 | protein coding | 235.41 | 0.37 | 0.0024 | 0.3584 |
| *Eln* | chr5-134702593-134747323 | protein coding | 135.32 | 0.79 | 0.0024 | 0.3594 |
| *Mrgprf* | chr7-145300828-145309557 | protein coding | 12.28 | 1.40 | 0.0026 | 0.3808 |
| *Alpk3* | chr7-81057600-81105612 | protein coding | 13.57 | 1.39 | 0.0027 | 0.3891 |
| *Plcb4* | chr2-135659011-136014593 | protein coding | 749.33 | 0.64 | 0.0027 | 0.3893 |
| *Rims3* | chr4-120854816-120896579 | protein coding | 1373.88 | 0.65 | 0.0029 | 0.4030 |
| *Col5a1* | chr2-27886425-28039514 | protein coding | 238.15 | 0.74 | 0.0030 | 0.4133 |
| *Cfh* | chr1-140084708-140183764 | protein coding | 545.50 | 0.60 | 0.0030 | 0.4133 |
| *Colec12* | chr18-9707595-9882644 | protein coding | 214.26 | 0.64 | 0.0030 | 0.4133 |
| *Adgrg5* | chr8-94923694-94943280 | protein coding | 1.70 | 3.61 | 0.0031 | NA |
| *Plekhg1* | chr10-3740364-3967303 | protein coding | 1066.88 | 0.68 | 0.0031 | 0.4225 |
| *Lilrb4a* | chr10-51490956-51496611 | protein coding | 5.76 | 1.86 | 0.0031 | 0.4233 |
| *Ccrl2* | chr9-111054486-111057519 | protein coding | 20.76 | 0.92 | 0.0031 | 0.4233 |
| *Gm49674* | chr16-74873039-74875938 | TEC | 1.46 | 3.71 | 0.0032 | NA |
| *Gm44798* | chr8-9618153-9620562 | TEC | 2.40 | 3.97 | 0.0032 | 0.4295 |
| *Tepp* | chr8-95310566-95326323 | protein coding | 2.19 | 3.86 | 0.0033 | 0.4295 |
| *Rnu12* | chr15-83149644-83149794 | snRNA | 2.23 | 3.40 | 0.0033 | 0.4295 |
| *Fmod* | chr1-134037254-134048277 | protein coding | 185.82 | 1.42 | 0.0033 | 0.4295 |
| *Gm33699* | chr9-61032212-61059116 | lncRNA | 19.78 | 0.91 | 0.0033 | 0.4295 |
| *Shox2* | chr3-66971727-66981771 | protein coding | 59.42 | 2.47 | 0.0033 | 0.4335 |
| *Cd40* | chr2-165055627-165072948 | protein coding | 11.19 | 1.19 | 0.0034 | 0.4362 |
| *A4galt* | chr15-83226722-83251774 | protein coding | 37.45 | 0.86 | 0.0035 | 0.4403 |
| *Tcf7l2* | chr19-55741820-55933654 | protein coding | 249.83 | 1.43 | 0.0035 | 0.4403 |
| *Trhr2* | chr8-122356967-122360746 | protein coding | 4.67 | 2.32 | 0.0035 | 0.4403 |
| *Angpt2* | chr8-18690263-18741562 | protein coding | 35.07 | 0.88 | 0.0035 | 0.4403 |
| *Pear1* | chr3-87747890-87824306 | protein coding | 120.21 | 0.64 | 0.0036 | 0.4423 |
| *Crispld2* | chr8-119992438-120052793 | protein coding | 46.98 | 0.96 | 0.0036 | 0.4423 |
| *Col4a5* | chrX-141475385-141689234 | protein coding | 138.20 | 0.40 | 0.0037 | 0.4423 |
| *Gm22175* | chr12-113425150-113425227 | miRNA | 1.53 | 3.53 | 0.0038 | NA |
| *Tbx2* | chr11-85832551-85841948 | protein coding | 42.99 | 0.68 | 0.0038 | 0.4575 |
| *Ptprb* | chr10-116275523-116389535 | protein coding | 907.40 | 0.49 | 0.0039 | 0.4575 |
| *Rny1* | chr6-47788069-47788180 | miscRNA | 69.91 | 2.43 | 0.0039 | 0.4575 |
| *Lama1* | chr17-67697259-67822647 | protein coding | 75.30 | 0.70 | 0.0040 | 0.4575 |
| *Fgfbp1* | chr5-43978858-43981779 | protein coding | 11.27 | 1.26 | 0.0040 | 0.4593 |
| *Fzd8* | chr18-9212163-9218136 | protein coding | 128.20 | 0.45 | 0.0040 | 0.4607 |
| *Arhgef40* | chr14-51984719-52006251 | protein coding | 570.76 | 0.32 | 0.0041 | 0.4608 |
| *Fbxo40* | chr16-36963460-36990467 | protein coding | 4.64 | 2.49 | 0.0041 | 0.4608 |
| *Lef1os1* | chr3-131109026-131112090 | lncRNA | 3.33 | 2.38 | 0.0041 | 0.4640 |
| *Gm43429* | chr3-135860348-135862598 | TEC | 1.54 | 3.83 | 0.0041 | NA |
| *Col9a2* | chr4-121039385-121055322 | protein coding | 97.52 | 0.96 | 0.0041 | 0.4640 |
| *Adam33* | chr2-131050591-131063814 | protein coding | 8.68 | 1.50 | 0.0043 | 0.4698 |
| *Bmp7* | chr2-172868012-172940321 | protein coding | 118.93 | 0.88 | 0.0043 | 0.4698 |
| *Wnt6* | chr1-74771892-74785322 | protein coding | 10.72 | 1.59 | 0.0043 | 0.4700 |
| *Gja5* | chr3-96904693-97077416 | protein coding | 8.62 | 1.33 | 0.0043 | 0.4702 |
| *Cp* | chr3-19957054-20009145 | protein coding | 368.35 | 0.60 | 0.0044 | 0.4711 |
| *H2-Ab1* | chr17-34257689-34269419 | protein coding | 45.83 | 0.93 | 0.0044 | 0.4745 |
| *Slc6a12* | chr6-121343076-121365775 | protein coding | 31.76 | 1.48 | 0.0045 | 0.4745 |
| *D7Bwg0826e* | chr7-44894305-44898099 | TEC | 12.96 | 1.09 | 0.0046 | 0.4824 |
| *Col1a2* | chr6-4504814-4541544 | protein coding | 305.63 | 0.82 | 0.0047 | 0.4852 |
| *Rnf152* | chr1-105276914-105356710 | protein coding | 215.97 | 0.71 | 0.0047 | 0.4852 |
| *Bgn* | chrX-73483602-73495933 | protein coding | 375.76 | 0.41 | 0.0047 | 0.4863 |
| *Samd9l* | chr6-3372257-3399572 | protein coding | 89.07 | 0.53 | 0.0049 | 0.4952 |
| *Irx3* | chr8-91798525-91802067 | protein coding | 12.41 | 1.17 | 0.0050 | 0.4997 |
| *Siah3* | chr14-75455982-75529708 | protein coding | 229.08 | 0.38 | 0.0052 | 0.5119 |
| *Spp1* | chr5-104435118-104441050 | protein coding | 116.50 | 1.37 | 0.0052 | 0.5119 |
| *Igfbp2* | chr1-72824503-72852474 | protein coding | 586.32 | 0.65 | 0.0053 | 0.5149 |
| *Gm10048* | chr5-48924954-48928740 | TEC | 3.55 | 2.39 | 0.0053 | 0.5149 |
| *Crabp2* | chr3-87948666-87953376 | protein coding | 31.15 | 1.19 | 0.0054 | 0.5149 |
| *H2-Eb1* | chr17-34305867-34316674 | protein coding | 50.06 | 0.86 | 0.0054 | 0.5149 |
| *Pln* | chr10-53337667-53345987 | protein coding | 37.33 | 1.07 | 0.0054 | 0.5149 |
| *Zbp1* | chr2-173206612-173218923 | protein coding | 8.59 | 1.79 | 0.0054 | 0.5149 |
| *Ntrk1* | chr3-87778244-87795162 | protein coding | 1.58 | 3.50 | 0.0054 | NA |
| *Tbx18* | chr9-87702800-87731260 | protein coding | 54.39 | 0.82 | 0.0056 | 0.5246 |
| *Gm38379* | chr8-120109441-120110965 | TEC | 3.81 | 2.31 | 0.0056 | 0.5246 |
| *Chad* | chr11-94565047-94569127 | protein coding | 3.06 | 2.40 | 0.0056 | 0.5246 |
| *Klrk1* | chr6-129610323-129623864 | protein coding | 1.95 | 3.61 | 0.0056 | NA |
| *Cit* | chr5-115845278-116008947 | protein coding | 998.86 | 0.74 | 0.0057 | 0.5246 |
| *Fhdc1* | chr3-84442198-84480429 | protein coding | 82.65 | 0.69 | 0.0057 | 0.5246 |
| *Neil2* | chr14-63182443-63193856 | protein coding | 53.80 | 0.52 | 0.0059 | 0.5358 |
| *Tspan11* | chr6-127887589-127953977 | protein coding | 40.50 | 1.28 | 0.0060 | 0.5358 |
| *Tmem163* | chr1-127486546-127679548 | protein coding | 149.69 | 0.65 | 0.0060 | 0.5358 |
| *Fam83h* | chr15-76001093-76014336 | protein coding | 21.89 | 0.85 | 0.0060 | 0.5358 |
| *Slc13a3* | chr2-165405028-165473230 | protein coding | 321.24 | 0.50 | 0.0061 | 0.5400 |
| *Fzd10* | chr5-128600844-128604093 | protein coding | 25.39 | 1.31 | 0.0063 | 0.5461 |
| *Ier3* | chr17-35821684-35822923 | protein coding | 64.50 | 0.52 | 0.0064 | 0.5461 |
| *Rny3* | chr6-47781624-47781725 | miscRNA | 22.54 | 2.45 | 0.0064 | 0.5461 |
| *Klhl6* | chr16-19946496-19983037 | protein coding | 59.58 | 0.67 | 0.0065 | 0.5461 |
| *Gckr* | chr5-31297443-31327314 | protein coding | 7.92 | 1.90 | 0.0065 | 0.5461 |
| *Mctp2* | chr7-72077830-72306608 | protein coding | 37.17 | 1.00 | 0.0065 | 0.5461 |
| *Mmp15* | chr8-95352268-95375080 | protein coding | 341.13 | 0.34 | 0.0065 | 0.5461 |
| *Vipr2* | chr12-116077726-116146261 | protein coding | 47.53 | 1.62 | 0.0066 | 0.5461 |
| *Spdef* | chr17-27714352-27728955 | protein coding | 4.38 | 2.31 | 0.0066 | 0.5461 |
| *Camk2d* | chr3-126596302-126846326 | protein coding | 894.71 | 0.44 | 0.0066 | 0.5461 |
| *Slc39a8* | chr3-135825279-135888572 | protein coding | 89.76 | 0.46 | 0.0067 | 0.5461 |
| *Gm22513* | chr12-54696782-54696945 | snRNA | 5.57 | 3.41 | 0.0067 | 0.5461 |
| *Wnt2b* | chr3-104945272-104961921 | protein coding | 7.29 | 1.77 | 0.0067 | 0.5461 |
| *Gli1* | chr10-127329882-127341974 | protein coding | 36.41 | 0.79 | 0.0068 | 0.5529 |
| *Chrnb3* | chr8-27368711-27399730 | protein coding | 28.70 | 1.16 | 0.0070 | 0.5611 |
| *Arhgap29* | chr3-121952541-122016753 | protein coding | 501.28 | 0.35 | 0.0071 | 0.5611 |
| *Pth1r* | chr9-110722085-110747145 | protein coding | 97.42 | 0.49 | 0.0071 | 0.5611 |
| *Ptgfrn* | chr3-101040232-101110278 | protein coding | 245.28 | 0.41 | 0.0071 | 0.5611 |
| *Edn1* | chr13-42301476-42307990 | protein coding | 45.73 | 0.73 | 0.0071 | 0.5611 |
| *Sema3f* | chr9-107681500-107710475 | protein coding | 51.16 | 0.97 | 0.0071 | 0.5611 |
| *Slc22a8* | chr19-8591254-8611835 | protein coding | 351.04 | 0.45 | 0.0072 | 0.5611 |
| *Podxl* | chr6-31519488-31563981 | protein coding | 671.43 | 0.47 | 0.0073 | 0.5656 |
| *Soat2* | chr15-102150526-102163469 | protein coding | 2.36 | 3.31 | 0.0074 | 0.5687 |
| *Ldc1* | chr4-130209109-130222401 | protein coding | 2.58 | 3.14 | 0.0075 | 0.5744 |
| *Ankrd34c* | chr9-89725245-89738475 | protein coding | 57.11 | 1.15 | 0.0076 | 0.5744 |
| *Coch* | chr12-51593341-51605771 | protein coding | 119.72 | 0.88 | 0.0076 | 0.5744 |
| *Plbd1* | chr6-136612070-136661928 | protein coding | 4.80 | 1.76 | 0.0076 | 0.5744 |
| *Lyz2* | chr10-117277331-117282321 | protein coding | 245.15 | 0.76 | 0.0076 | 0.5747 |
| *Trpc3* | chr3-36620482-36690167 | protein coding | 154.08 | 0.68 | 0.0077 | 0.5747 |
| *Gm27320* | chr8-105307792-105307900 | miRNA | 7.79 | 1.54 | 0.0078 | 0.5817 |
| *Coro6* | chr11-77462411-77470484 | protein coding | 482.68 | 0.68 | 0.0079 | 0.5834 |
| *Ddr2* | chr1-169972307-170110762 | protein coding | 296.81 | 0.33 | 0.0079 | 0.5834 |
| *Grm4* | chr17-27422387-27521403 | protein coding | 281.31 | 0.58 | 0.0080 | 0.5866 |
| *Als2cl* | chr9-110879870-110900530 | protein coding | 285.93 | 0.39 | 0.0080 | 0.5866 |
| *Vwf* | chr6-125546774-125686679 | protein coding | 324.66 | 0.59 | 0.0081 | 0.5884 |
| *Ano1* | chr7-144588549-144751974 | protein coding | 104.57 | 0.59 | 0.0081 | 0.5885 |
| *Fpgs* | chr2-32682609-32704145 | protein coding | 175.17 | 0.32 | 0.0081 | 0.5885 |
| *Tsku* | chr7-98350668-98361328 | protein coding | 39.60 | 0.80 | 0.0083 | 0.5954 |
| *Ebf1* | chr11-44617317-45008091 | protein coding | 80.43 | 0.62 | 0.0084 | 0.5954 |
| *Gm42992* | chr5-131986549-131993384 | TEC | 1.44 | 3.98 | 0.0084 | NA |
| *Lsp1* | chr7-142460809-142494867 | protein coding | 63.51 | 0.55 | 0.0084 | 0.5955 |
| *Gm37077* | chr1-25755343-25759033 | TEC | 1.92 | 3.46 | 0.0085 | NA |
| *Foxb1* | chr9-69757710-69760940 | protein coding | 4.07 | 2.75 | 0.0085 | 0.5965 |
| *Mab21l2* | chr3-86545581-86548629 | protein coding | 12.79 | 1.64 | 0.0085 | 0.5965 |
| *Ccdc136* | chr6-29396309-29426994 | protein coding | 1112.37 | 0.38 | 0.0087 | 0.6024 |
| *Npsr1* | chr9-24097996-24316398 | protein coding | 11.62 | 1.59 | 0.0087 | 0.6024 |
| *Hspa12b* | chr2-131127280-131146321 | protein coding | 142.15 | 0.43 | 0.0087 | 0.6024 |
| *Mmrn2* | chr14-34375465-34404287 | protein coding | 89.27 | 0.63 | 0.0087 | 0.6024 |
| *Cxcl10* | chr5-92346638-92348889 | protein coding | 14.28 | 1.99 | 0.0091 | 0.6203 |
| *Fbln1* | chr15-85205949-85286535 | protein coding | 243.65 | 0.48 | 0.0092 | 0.6301 |
| *Gm38336* | chr1-34809360-34811908 | lncRNA | 4.50 | 1.97 | 0.0093 | 0.6331 |
| *Agt* | chr8-124556534-124569706 | protein coding | 527.83 | 0.88 | 0.0094 | 0.6331 |
| *Cavin1* | chr11-100956733-100970887 | protein coding | 395.04 | 0.49 | 0.0094 | 0.6331 |
| *Slc6a20a* | chr9-123634770-123678885 | protein coding | 299.76 | 0.79 | 0.0095 | 0.6341 |
| *Tnfaip2* | chr12-111442469-111455018 | protein coding | 19.17 | 0.98 | 0.0096 | 0.6370 |
| *Relt* | chr7-100845847-100863446 | protein coding | 241.18 | 0.33 | 0.0097 | 0.6405 |
| *Foxp2* | chr6-14901349-15441977 | protein coding | 53.27 | 0.91 | 0.0097 | 0.6405 |
| *Foxc1* | chr13-31806633-31812476 | protein coding | 87.00 | 0.75 | 0.0097 | 0.6405 |
| *Mcm10* | chr2-4989714-5012791 | protein coding | 14.27 | 0.96 | 0.0099 | 0.6448 |
| *Tal1* | chr4-115056426-115071755 | protein coding | 50.90 | 0.59 | 0.0099 | 0.6448 |
| *Cenpn* | chr8-116921730-116941507 | protein coding | 20.26 | 0.76 | 0.0101 | 0.6494 |
| *Irak3* | chr10-120141648-120202130 | protein coding | 19.87 | 0.94 | 0.0102 | 0.6567 |
| *Arhgef5* | chr6-43265582-43289320 | protein coding | 80.73 | 0.41 | 0.0103 | 0.6571 |
| *Ramp3* | chr11-6658521-6677475 | protein coding | 144.01 | 1.05 | 0.0104 | 0.6585 |
| *Card9* | chr2-26352176-26360918 | protein coding | 50.09 | 0.52 | 0.0104 | 0.6585 |
| *Fblim1* | chr4-141576062-141606096 | protein coding | 19.89 | 0.77 | 0.0106 | 0.6682 |
| *Jag1* | chr2-137081456-137116644 | protein coding | 216.01 | 0.61 | 0.0108 | 0.6689 |
| *Gm13489* | chr2-48809585-48810555 | processed pseudogene | 0.87 | 3.71 | 0.0108 | NA |
| *Ccdc9b* | chr2-118754158-118762661 | protein coding | 269.72 | 0.38 | 0.0109 | 0.6764 |
| *Col4a6* | chrX-141165403-141474076 | protein coding | 49.04 | 0.69 | 0.0110 | 0.6800 |
| *Cd248* | chr19-5068078-5070682 | protein coding | 66.11 | 0.73 | 0.0111 | 0.6800 |
| *Esyt1* | chr10-128509965-128525871 | protein coding | 210.51 | 0.39 | 0.0111 | 0.6812 |
| *Gm42583* | chr6-21258921-21265620 | TEC | 6.75 | 1.89 | 0.0112 | 0.6847 |
| *Ccdc88b* | chr19-6844623-6858211 | protein coding | 110.14 | 0.42 | 0.0113 | 0.6857 |
| *Omd* | chr13-49582462-49592822 | protein coding | 14.42 | 1.34 | 0.0114 | 0.6923 |
| *Alox12* | chr11-70241457-70255353 | protein coding | 16.00 | 0.81 | 0.0117 | 0.7057 |
| *Cd44* | chr2-102811141-102901665 | protein coding | 68.30 | 0.76 | 0.0119 | 0.7120 |
| *Acacb* | chr5-114146535-114250761 | protein coding | 138.29 | 0.49 | 0.0120 | 0.7137 |
| *4930534D22Rik* | chr3-131302643-131316661 | lncRNA | 6.23 | 1.54 | 0.0121 | 0.7193 |
| *Sspo* | chr6-48448229-48501250 | protein coding | 126.67 | 1.68 | 0.0122 | 0.7194 |
| *Smtn* | chr11-3517523-3540612 | protein coding | 170.88 | 0.48 | 0.0124 | 0.7210 |
| *Gata2* | chr6-88193891-88207032 | protein coding | 72.89 | 0.54 | 0.0124 | 0.7210 |
| *Bend7* | chr2-4717831-4802142 | protein coding | 41.82 | 0.52 | 0.0124 | 0.7210 |
| *Pik3r6* | chr11-68503019-68552698 | protein coding | 42.71 | 0.54 | 0.0125 | 0.7210 |
| *Pecam1* | chr11-106654217-106750628 | protein coding | 414.62 | 0.45 | 0.0126 | 0.7210 |
| *Adgra1* | chr7-139834174-139878088 | protein coding | 855.93 | 0.57 | 0.0126 | 0.7210 |
| *Clec14a* | chr12-58264720-58269290 | protein coding | 86.11 | 0.66 | 0.0131 | 0.7300 |
| *F2r* | chr13-95601803-95618487 | protein coding | 133.15 | 0.54 | 0.0131 | 0.7316 |
| *Nipal2* | chr15-34572799-34679212 | protein coding | 116.47 | 0.44 | 0.0132 | 0.7327 |
| *Aebp1* | chr11-5861947-5872088 | protein coding | 363.24 | 0.72 | 0.0134 | 0.7328 |
| *Mpzl2* | chr9-45039799-45054015 | protein coding | 23.45 | 1.04 | 0.0134 | 0.7328 |
| *Erich2os* | chr2-70503433-70509199 | lncRNA | 1.71 | 2.93 | 0.0134 | NA |
| *Ano7* | chr1-93373930-93404303 | protein coding | 6.72 | 1.46 | 0.0135 | 0.7328 |
| *Dab2* | chr15-6299788-6440712 | protein coding | 232.33 | 0.38 | 0.0135 | 0.7328 |
| *Socs3* | chr11-117966079-117970047 | protein coding | 29.06 | 0.65 | 0.0135 | 0.7328 |
| *Cbr2* | chr11-120729489-120732114 | protein coding | 19.33 | 0.92 | 0.0135 | 0.7328 |
| *Pou4f1* | chr14-104461676-104467999 | protein coding | 15.18 | 1.71 | 0.0135 | 0.7328 |
| *Btbd11* | chr10-85386814-85660292 | protein coding | 358.57 | 0.36 | 0.0136 | 0.7328 |
| *Dennd3* | chr15-73512560-73572242 | protein coding | 128.12 | 0.45 | 0.0137 | 0.7357 |
| *Gm19461* | chr1-133250398-133269808 | lncRNA | 10.16 | 1.27 | 0.0137 | 0.7372 |
| *Susd2* | chr10-75636706-75644008 | protein coding | 61.35 | 0.61 | 0.0138 | 0.7372 |
| *A330074K22Rik* | chr8-120204434-120228230 | lncRNA | 50.91 | 0.65 | 0.0140 | 0.7426 |
| *Slc22a6* | chr19-8618039-8628299 | protein coding | 89.74 | 1.31 | 0.0140 | 0.7426 |
| *Des* | chr1-75360329-75368579 | protein coding | 41.55 | 0.66 | 0.0142 | 0.7455 |
| *Vwa5b2* | chr16-20589471-20605377 | protein coding | 599.77 | 0.38 | 0.0142 | 0.7455 |
| *Eqtn* | chr4-94907267-94928843 | protein coding | 26.20 | 0.70 | 0.0143 | 0.7455 |
| *Gm38286* | chr1-25345978-25349082 | TEC | 1.09 | 3.72 | 0.0143 | NA |
| *Nfatc4* | chr14-55823144-55833943 | protein coding | 35.38 | 0.64 | 0.0144 | 0.7473 |
| *Tfpi* | chr2-84432855-84476775 | protein coding | 89.99 | 0.51 | 0.0144 | 0.7484 |
| *Tgm2* | chr2-158116402-158146436 | protein coding | 257.58 | 0.53 | 0.0145 | 0.7484 |
| *Slc6a13* | chr6-121300227-121337733 | protein coding | 214.64 | 1.24 | 0.0145 | 0.7484 |
| *Snord17* | chr2-144265979-144266216 | snoRNA | 11.15 | 1.84 | 0.0146 | 0.7520 |
| *8030474K03Rik* | chrX-101794656-101798642 | protein coding | 1.27 | 4.68 | 0.0147 | NA |
| *Mical1* | chr10-41476314-41487032 | protein coding | 243.48 | 0.34 | 0.0151 | 0.7543 |
| *Sell* | chr1-164061982-164084181 | protein coding | 1.77 | 3.54 | 0.0151 | NA |
| *Myof* | chr19-37899036-38043577 | protein coding | 92.92 | 0.64 | 0.0151 | 0.7543 |
| *Gm43693* | chr5-32984570-32985818 | TEC | 18.64 | 0.95 | 0.0152 | 0.7543 |
| *Cd33* | chr7-43524216-43544428 | protein coding | 300.57 | 0.37 | 0.0152 | 0.7543 |
| *Sned1* | chr1-93235841-93301065 | protein coding | 587.89 | 0.42 | 0.0152 | 0.7543 |
| *Gata3* | chr2-9857078-9890034 | protein coding | 11.31 | 1.69 | 0.0154 | 0.7592 |
| *9030612E09Rik* | chr10-43174699-43176562 | lncRNA | 18.73 | 0.78 | 0.0155 | 0.7619 |
| *Acta2* | chr19-34241090-34255590 | protein coding | 345.25 | 1.04 | 0.0156 | 0.7657 |
| *Zic1* | chr9-91358058-91365810 | protein coding | 611.83 | 0.93 | 0.0156 | 0.7657 |
| *Pygl* | chr12-70190811-70231488 | protein coding | 103.64 | 0.49 | 0.0157 | 0.7669 |
| *Fn1* | chr1-71585520-71653200 | protein coding | 1071.32 | 0.62 | 0.0159 | 0.7708 |
| *Bcam* | chr7-19756131-19771016 | protein coding | 272.69 | 0.43 | 0.0160 | 0.7719 |
| *Rxfp2* | chr5-150018675-150082184 | protein coding | 7.28 | 1.30 | 0.0160 | 0.7734 |
| *Kcnk12* | chr17-87745801-87797994 | protein coding | 121.80 | 0.38 | 0.0161 | 0.7746 |
| *Gm39929* | chr2-128601765-128609630 | lncRNA | 1.68 | 2.97 | 0.0161 | NA |
| *Cyp4x1os* | chr4-115133832-115137864 | lncRNA | 17.84 | 0.86 | 0.0161 | 0.7746 |
| *Vtn* | chr11-78499091-78502324 | protein coding | 1514.27 | 0.52 | 0.0165 | 0.7825 |
| *Gm45442* | chr7-45922215-45924174 | lncRNA | 2.37 | 2.42 | 0.0166 | 0.7839 |
| *Pard6g* | chr18-80046890-80119639 | protein coding | 111.94 | 0.32 | 0.0166 | 0.7839 |
| *Esrrb* | chr12-86361117-86521628 | protein coding | 34.25 | 0.62 | 0.0167 | 0.7839 |
| *Sema3g* | chr14-31217860-31230352 | protein coding | 122.92 | 0.50 | 0.0168 | 0.7862 |
| *Gm26796* | chr12-80758406-80762793 | lncRNA | 3.55 | 1.94 | 0.0168 | 0.7869 |
| *Lrch4* | chr5-137629121-137641099 | protein coding | 3.36 | 2.07 | 0.0169 | 0.7881 |
| *Fzd5* | chr1-64730558-64737751 | protein coding | 34.50 | 0.68 | 0.0170 | 0.7903 |
| *Cbln2* | chr18-86711110-86718283 | protein coding | 151.88 | 0.59 | 0.0171 | 0.7909 |
| *Clic5* | chr17-44134768-44280172 | protein coding | 160.43 | 0.46 | 0.0171 | 0.7909 |
| *Galnt15* | chr14-32028989-32062197 | protein coding | 62.90 | 0.48 | 0.0171 | 0.7909 |
| *Chrna6* | chr8-27403212-27413944 | protein coding | 9.53 | 1.56 | 0.0172 | 0.7922 |
| *Gm47122* | chr9-122119145-122119495 | TEC | 1.14 | 3.47 | 0.0173 | NA |
| *Vwc2* | chr11-11114223-11268931 | protein coding | 104.80 | 0.41 | 0.0174 | 0.7931 |
| *Gm42993* | chr5-132015610-132022934 | TEC | 2.12 | 3.40 | 0.0175 | NA |
| *Wnt3* | chr11-103774150-103817957 | protein coding | 69.79 | 0.72 | 0.0178 | 0.7994 |
| *Col6a2* | chr10-76595762-76623630 | protein coding | 120.73 | 0.65 | 0.0179 | 0.7994 |
| *Nanos2* | chr7-18987400-18988962 | protein coding | 17.00 | 0.82 | 0.0180 | 0.8023 |
| *Tnfrsf1b* | chr4-145213463-145246870 | protein coding | 112.85 | 0.39 | 0.0180 | 0.8023 |
| *Prorp* | chr12-55299577-55382533 | protein coding | 150.91 | 0.34 | 0.0181 | 0.8023 |
| *Ttc39aos1* | chr4-109403794-109406155 | lncRNA | 21.93 | 0.97 | 0.0181 | 0.8023 |
| *Fcrl1* | chr3-87376387-87402934 | protein coding | 13.11 | 0.89 | 0.0182 | 0.8023 |
| *Pidd1* | chr7-141438113-141444025 | protein coding | 37.82 | 0.54 | 0.0183 | 0.8023 |
| *Loxhd1* | chr18-77281958-77442930 | protein coding | 3.15 | 1.78 | 0.0183 | 0.8023 |
| *Bmp6* | chr13-38345107-38500302 | protein coding | 177.61 | 0.65 | 0.0183 | 0.8023 |
| *Dio3os* | chr12-110275385-110278172 | lncRNA | 2.53 | 2.41 | 0.0183 | 0.8023 |
| *Cgnl1* | chr9-71626509-71771602 | protein coding | 356.08 | 0.48 | 0.0186 | 0.8130 |
| *Map3k8* | chr18-4331327-4353015 | protein coding | 46.03 | 0.54 | 0.0186 | 0.8130 |
| *Vstm4* | chr14-32856756-32939491 | protein coding | 97.59 | 0.56 | 0.0188 | 0.8131 |
| *Slc22a4* | chr11-53983123-54028090 | protein coding | 163.22 | 0.38 | 0.0188 | 0.8131 |
| *Rpgrip1* | chr14-52110704-52163546 | protein coding | 18.16 | 0.74 | 0.0190 | 0.8159 |
| *Tacstd2* | chr6-67534062-67535796 | protein coding | 1.26 | 3.19 | 0.0190 | NA |
| *Clcf1* | chr19-4214238-4223490 | protein coding | 27.76 | 0.71 | 0.0191 | 0.8159 |
| *Myo5c* | chr9-75232020-75305451 | protein coding | 21.01 | 0.99 | 0.0191 | 0.8159 |
| *Trim25* | chr11-88999376-89020293 | protein coding | 215.67 | 0.35 | 0.0191 | 0.8159 |
| *Rdh1* | chr10-127759721-127768297 | protein coding | 2.31 | 2.81 | 0.0191 | 0.8159 |
| *Sp100* | chr1-85649988-85709998 | protein coding | 97.46 | 0.48 | 0.0193 | 0.8161 |
| *Dock6* | chr9-21799860-21852635 | protein coding | 486.08 | 0.31 | 0.0193 | 0.8161 |
| *Gm29695* | chr1-132191457-132209546 | protein coding | 19.14 | 0.76 | 0.0193 | 0.8161 |
| *Abca12* | chr1-71242276-71414910 | protein coding | 3.63 | 2.18 | 0.0195 | 0.8162 |
| *Slc25a24* | chr3-109123149-109168457 | protein coding | 52.89 | 0.61 | 0.0195 | 0.8162 |
| *Gm20696* | chr6-99257484-99666797 | protein coding | 0.87 | 3.56 | 0.0197 | NA |
| *Cped1* | chr6-21985916-22256404 | protein coding | 71.05 | 0.60 | 0.0197 | 0.8162 |
| *Slc8a3* | chr12-81197915-81333180 | protein coding | 397.72 | 0.33 | 0.0198 | 0.8162 |
| *Gm26583* | chr12-112946363-112950512 | lncRNA | 3.82 | 1.47 | 0.0198 | 0.8162 |
| *Gpr182* | chr10-127747276-127751732 | protein coding | 32.99 | 0.87 | 0.0198 | 0.8162 |
| *Ptprc* | chr1-138062861-138175708 | protein coding | 131.09 | 0.50 | 0.0199 | 0.8162 |
| *Myl9* | chr2-156775420-156781658 | protein coding | 312.68 | 0.61 | 0.0201 | 0.8162 |
| *Ucn2* | chr9-108986010-108987164 | protein coding | 1.24 | 3.12 | 0.0201 | NA |
| *Gm37895* | chr1-25775757-25780306 | TEC | 1.80 | 3.03 | 0.0201 | NA |
| *Camk2n2* | chr16-20619215-20621287 | protein coding | 709.19 | 0.34 | 0.0201 | 0.8162 |
| *Gm46416* | chr13-55535995-55536624 | lncRNA | 2.62 | 2.10 | 0.0202 | 0.8162 |
| *Crybg1* | chr10-43950636-44148853 | protein coding | 24.25 | 0.89 | 0.0202 | 0.8162 |
| *Asap3* | chr4-136206365-136245216 | protein coding | 134.46 | 0.32 | 0.0202 | 0.8162 |
| *Rec8* | chr14-55618037-55625395 | protein coding | 61.51 | 0.52 | 0.0202 | 0.8162 |
| *Nphp3* | chr9-104002544-104043818 | protein coding | 238.18 | 0.38 | 0.0202 | 0.8162 |
| *Gm28905* | chr10-9976627-10207981 | lncRNA | 3.01 | 2.07 | 0.0203 | 0.8162 |
| *Tlcd2* | chr11-75461715-75470899 | protein coding | 7.60 | 1.14 | 0.0205 | 0.8162 |
| *Mak* | chr13-41025008-41079706 | protein coding | 35.37 | 0.78 | 0.0205 | 0.8162 |
| *Gm38056* | chr1-55440904-55445930 | TEC | 2.67 | 2.77 | 0.0205 | 0.8162 |
| *Ank1* | chr8-22974844-23150497 | protein coding | 1063.58 | 0.40 | 0.0206 | 0.8162 |
| *Gm49745* | chr16-20566358-20568508 | lncRNA | 13.07 | 1.01 | 0.0208 | 0.8162 |
| *Adamts19* | chr18-58836667-59054060 | protein coding | 14.19 | 2.32 | 0.0208 | 0.8162 |
| *Grin3b* | chr10-79970715-79977190 | protein coding | 5.27 | 1.47 | 0.0209 | 0.8162 |
| *Emilin1* | chr5-30913402-30921277 | protein coding | 56.79 | 0.61 | 0.0209 | 0.8162 |
| *Adgrf5* | chr17-43360451-43459557 | protein coding | 877.09 | 0.39 | 0.0209 | 0.8162 |
| *Mamdc2* | chr19-23302609-23448442 | protein coding | 43.77 | 0.50 | 0.0210 | 0.8162 |
| *Mypn* | chr10-63115795-63203952 | protein coding | 19.33 | 0.91 | 0.0210 | 0.8162 |
| *Slc6a11* | chr6-114131241-114249952 | protein coding | 3134.46 | 0.36 | 0.0211 | 0.8183 |
| *Gm37367* | chr1-158534807-158537408 | TEC | 1.14 | 3.72 | 0.0212 | NA |
| *Gm37628* | chr1-25686062-25691501 | TEC | 1.69 | 3.12 | 0.0214 | NA |
| *Olfr53* | chr7-140646452-140653085 | protein coding | 2.91 | 1.80 | 0.0215 | 0.8248 |
| *Cd163* | chr6-124304656-124330527 | protein coding | 18.08 | 1.06 | 0.0218 | 0.8321 |
| *Ccr2* | chr9-124101950-124113557 | protein coding | 8.62 | 1.54 | 0.0218 | 0.8321 |
| *Rassf3* | chr10-121410350-121476347 | protein coding | 424.58 | 0.31 | 0.0218 | 0.8325 |
| *Mpo* | chr11-87793581-87804413 | protein coding | 2.15 | 2.44 | 0.0219 | 0.8328 |
| *AB124611* | chr9-21526176-21545333 | protein coding | 5.51 | 1.40 | 0.0219 | 0.8328 |
| *Thbs2* | chr17-14665500-14694235 | protein coding | 143.14 | 0.47 | 0.0222 | 0.8424 |
| *1810059C17Rik* | chr2-18054193-18056613 | lncRNA | 2.77 | 1.93 | 0.0223 | 0.8424 |
| *Gm45178* | chr7-90995309-90999418 | TEC | 1.31 | 3.13 | 0.0223 | NA |
| *Cd209a* | chr8-3743397-3748984 | protein coding | 5.57 | 1.28 | 0.0225 | 0.8489 |
| *Wt1* | chr2-105126529-105173616 | protein coding | 1.63 | 3.71 | 0.0226 | NA |
| *Gdpd5* | chr7-99381414-99461877 | protein coding | 366.04 | 0.44 | 0.0228 | 0.8545 |
| *Fzd6* | chr15-39006034-39038188 | protein coding | 239.57 | 0.30 | 0.0230 | 0.8545 |
| *Gm25632* | chr2-32225653-32225737 | snoRNA | 4.93 | 1.36 | 0.0230 | 0.8545 |
| *Rassf4* | chr6-116633008-116673952 | protein coding | 182.17 | 0.43 | 0.0231 | 0.8545 |
| *C1s1* | chr6-124530345-124542359 | protein coding | 21.03 | 0.76 | 0.0233 | 0.8562 |
| *AU020206* | chr7-75769038-75782099 | lncRNA | 123.36 | 0.48 | 0.0233 | 0.8562 |
| *Rsad2* | chr12-26442746-26456452 | protein coding | 42.74 | 0.65 | 0.0233 | 0.8562 |
| *Gpr153* | chr4-152274232-152285337 | protein coding | 217.98 | 0.49 | 0.0234 | 0.8564 |
| *Aldh1a2* | chr9-71215789-71296243 | protein coding | 145.11 | 1.35 | 0.0236 | 0.8591 |
| *Ube2l6* | chr2-84798828-84810335 | protein coding | 111.52 | 0.32 | 0.0236 | 0.8591 |
| *Il13ra1* | chrX-36112110-36171259 | protein coding | 131.26 | 0.37 | 0.0237 | 0.8597 |
| *Lfng* | chr5-140607320-140615545 | protein coding | 258.92 | 0.33 | 0.0240 | 0.8626 |
| *Gm42866* | chr5-121742417-121745720 | TEC | 0.76 | 3.39 | 0.0241 | NA |
| *Gm20242* | chr14-28415997-28417851 | TEC | 1.42 | 2.91 | 0.0242 | NA |
| *Efhc1* | chr1-20951626-20990841 | protein coding | 34.29 | 0.60 | 0.0244 | 0.8626 |
| *Abca1* | chr4-53030787-53159895 | protein coding | 1322.36 | 0.32 | 0.0244 | 0.8626 |
| *BC035947* | chr1-78497026-78512158 | protein coding | 24.94 | 0.95 | 0.0245 | 0.8626 |
| *Flt3l* | chr7-45129788-45136432 | protein coding | 88.59 | 0.40 | 0.0245 | 0.8626 |
| *Gm37529* | chr1-160321686-160327078 | lncRNA | 2.60 | 2.65 | 0.0247 | 0.8626 |
| *Hlx* | chr1-184727140-184732619 | protein coding | 16.66 | 0.81 | 0.0247 | 0.8626 |
| *Mir6403* | chr4-134567723-134567851 | miRNA | 1.18 | 2.81 | 0.0247 | NA |
| *Akr1c14* | chr13-4049011-4090688 | protein coding | 40.22 | 0.51 | 0.0248 | 0.8636 |
| *Notch3* | chr17-32120820-32166852 | protein coding | 257.10 | 0.41 | 0.0249 | 0.8636 |
| *Treml2* | chr17-48299498-48312533 | protein coding | 13.71 | 1.03 | 0.0251 | 0.8665 |
| *Fbxo15* | chr18-84934782-84981473 | protein coding | 3.21 | 1.67 | 0.0252 | 0.8665 |
| *Art4* | chr6-136848451-136857733 | protein coding | 1.46 | 3.14 | 0.0253 | NA |
| *Acap1* | chr11-69881567-69895539 | protein coding | 3.87 | 1.37 | 0.0255 | 0.8750 |
| *Dpt* | chr1-164796644-164824266 | protein coding | 1.26 | 2.94 | 0.0256 | NA |
| *Trim34b* | chr7-104329471-104336909 | protein coding | 3.11 | 5.14 | 0.0257 | 0.8755 |
| *Gm37899* | chr12-109668869-109677585 | TEC | 3.31 | 2.56 | 0.0259 | 0.8775 |
| *Plce1* | chr19-38481109-38785030 | protein coding | 253.33 | 0.30 | 0.0260 | 0.8775 |
| *Pik3ap1* | chr19-41272377-41385102 | protein coding | 123.53 | 0.44 | 0.0262 | 0.8775 |
| *Asic4* | chr1-75450436-75474343 | protein coding | 160.76 | 0.45 | 0.0263 | 0.8775 |
| *Dnmbp* | chr19-43846821-43940191 | protein coding | 270.85 | 0.32 | 0.0263 | 0.8775 |
| *Gm45176* | chr7-91131841-91135936 | TEC | 1.45 | 3.40 | 0.0263 | NA |
| *AC163616.1* | chr3-90052904-90066069 | protein coding | 28.23 | 0.52 | 0.0263 | 0.8775 |
| *Zic2* | chr14-122475435-122479852 | protein coding | 331.63 | 0.44 | 0.0264 | 0.8775 |
| *Eme1* | chr11-94644996-94653964 | protein coding | 3.48 | 1.63 | 0.0265 | 0.8775 |
| *Kcna5* | chr6-126532551-126535412 | protein coding | 21.70 | 0.84 | 0.0267 | 0.8775 |
| *Gm44797* | chr8-9595109-9596945 | TEC | 1.03 | 3.14 | 0.0268 | NA |
| *Rttn* | chr18-88971790-89131016 | protein coding | 107.69 | 0.32 | 0.0269 | 0.8775 |
| *Il15ra* | chr2-11705290-11734317 | protein coding | 99.70 | 0.37 | 0.0271 | 0.8775 |
| *Gpsm3* | chr17-34589806-34591754 | protein coding | 26.54 | 0.70 | 0.0271 | 0.8775 |
| *Gm28703* | chr9-90825302-90828860 | lncRNA | 4.71 | 2.01 | 0.0272 | 0.8775 |
| *Slc12a7* | chr13-73733094-73816754 | protein coding | 129.10 | 0.35 | 0.0272 | 0.8775 |
| *Gm44676* | chr7-91608956-91612010 | TEC | 1.13 | 3.84 | 0.0274 | NA |
| *Heg1* | chr16-33684370-33771576 | protein coding | 660.04 | 0.37 | 0.0274 | 0.8775 |
| *Steap4* | chr5-7960457-7982213 | protein coding | 9.95 | 1.26 | 0.0275 | 0.8775 |
| *Cd5l* | chr3-87357881-87371073 | protein coding | 1.66 | 3.58 | 0.0276 | NA |
| *Gm15351* | chr8-12899146-12915758 | lncRNA | 13.96 | 1.00 | 0.0276 | 0.8775 |
| *Sgsh* | chr11-119343425-119355536 | protein coding | 150.78 | 0.33 | 0.0276 | 0.8775 |
| *Ppp1r18os* | chr17-35860918-35866886 | lncRNA | 1.59 | 2.69 | 0.0276 | NA |
| *Eif3j2* | chr18-43475418-43477796 | protein coding | 22.76 | 1.01 | 0.0276 | 0.8775 |
| *Gm42771* | chr5-49219676-49222161 | TEC | 2.28 | 2.95 | 0.0276 | 0.8775 |
| *Gm42918* | chr5-121385522-121387101 | lncRNA | 24.61 | 0.63 | 0.0277 | 0.8775 |
| *Pcna-ps2* | chr19-9283380-9284160 | processed pseudogene | 9.47 | 1.43 | 0.0279 | 0.8807 |
| *Gm15418* | chr8-11187755-11204503 | lncRNA | 2.30 | 2.18 | 0.0280 | 0.8807 |
| *Lama4* | chr10-38965515-39110188 | protein coding | 112.17 | 0.34 | 0.0283 | 0.8808 |
| *Fas* | chr19-34290666-34327772 | protein coding | 31.64 | 0.56 | 0.0284 | 0.8808 |
| *Gm38120* | chr1-13343563-13348204 | TEC | 1.36 | 2.80 | 0.0285 | NA |
| *Gm48086* | chr10-61446902-61450717 | lncRNA | 2.53 | 2.15 | 0.0285 | 0.8809 |
| *Arhgef15* | chr11-68943155-68957480 | protein coding | 183.14 | 0.45 | 0.0286 | 0.8840 |
| *Gm50012* | chr17-86970372-86975288 | lncRNA | 7.80 | 1.09 | 0.0287 | 0.8840 |
| *Slc27a5* | chr7-12988346-12998192 | protein coding | 8.40 | 1.01 | 0.0288 | 0.8840 |
| *Grin2d* | chr7-45831883-45878378 | protein coding | 289.05 | 0.33 | 0.0288 | 0.8840 |
| *Gm37862* | chr1-25325866-25328398 | TEC | 0.72 | 3.40 | 0.0289 | NA |
| *Epn3* | chr11-94489599-94499974 | protein coding | 78.35 | 0.88 | 0.0289 | 0.8844 |
| *Gm16938* | chr7-98177194-98179401 | lncRNA | 6.64 | 1.11 | 0.0290 | 0.8849 |
| *Helz2* | chr2-181227615-181242027 | protein coding | 126.21 | 0.43 | 0.0292 | 0.8874 |
| *Rora* | chr9-68653786-69388246 | protein coding | 1005.92 | 0.70 | 0.0293 | 0.8874 |
| *Lca5l* | chr16-96158407-96192271 | protein coding | 65.75 | 0.45 | 0.0293 | 0.8874 |
| *Mmp19* | chr10-128790910-128800824 | protein coding | 34.53 | 0.81 | 0.0296 | 0.8903 |
| *Map3k1* | chr13-111746428-111808993 | protein coding | 248.73 | 0.36 | 0.0300 | 0.8938 |
| *Pla2g4e* | chr2-120166412-120245335 | protein coding | 157.75 | 1.09 | 0.0300 | 0.8938 |
| *Gm25939* | chr1-72255008-72255198 | snRNA | 6.88 | 1.68 | 0.0300 | 0.8938 |
| *Lncpint* | chr6-31082565-31220488 | lncRNA | 14.37 | 1.02 | 0.0301 | 0.8938 |
| *Gpat2* | chr2-127425199-127436092 | protein coding | 3.42 | 1.95 | 0.0302 | 0.8938 |
| *Bmf* | chr2-118528757-118549687 | protein coding | 96.86 | 0.40 | 0.0302 | 0.8938 |
| *Itih2* | chr2-10094593-10131396 | protein coding | 76.07 | 1.07 | 0.0303 | 0.8938 |
| *Mtmr11* | chr3-96162004-96171718 | protein coding | 165.50 | 0.32 | 0.0303 | 0.8938 |
| *Gm4759* | chr7-106417848-106424469 | unprocessed pseudogene | 1.97 | 3.69 | 0.0304 | NA |
| *Syt2* | chr1-134646677-134762593 | protein coding | 651.43 | 0.49 | 0.0306 | 0.8979 |
| *Filip1l* | chr16-57353093-57573126 | protein coding | 82.82 | 0.51 | 0.0308 | 0.8999 |
| *Cspg4* | chr9-56865033-56899870 | protein coding | 517.93 | 0.31 | 0.0308 | 0.8999 |
| *Gm44679* | chr7-91519115-91522022 | TEC | 1.04 | 3.56 | 0.0308 | NA |
| *Gm11745* | chr11-117134598-117135045 | processed pseudogene | 1.48 | 2.83 | 0.0309 | NA |
| *Mir344i* | chr7-62085223-62085310 | miRNA | 1.10 | 3.20 | 0.0309 | NA |
| *Plekhd1* | chr12-80692591-80724214 | protein coding | 119.67 | 0.62 | 0.0309 | 0.8999 |
| *Rnf213* | chr11-119393100-119487418 | protein coding | 405.34 | 0.43 | 0.0311 | 0.8999 |
| *Otoa* | chr7-121081650-121163097 | protein coding | 3.61 | 1.54 | 0.0312 | 0.8999 |
| *Antxr1* | chr6-87133853-87335821 | protein coding | 302.44 | 0.31 | 0.0314 | 0.9039 |
| *Gm43571* | chr5-121715109-121718206 | TEC | 1.31 | 2.81 | 0.0315 | NA |
| *Gpr4* | chr7-19212538-19224174 | protein coding | 72.31 | 0.43 | 0.0316 | 0.9039 |
| *Gm44618* | chr7-28629248-28681936 | lncRNA | 1.54 | 2.70 | 0.0317 | NA |
| *Trpm6* | chr19-18749983-18892511 | protein coding | 14.89 | 0.96 | 0.0318 | 0.9039 |
| *Tmem164* | chrX-142681406-142843494 | protein coding | 345.93 | 0.36 | 0.0318 | 0.9039 |
| *Hmcn2* | chr2-31314415-31460738 | protein coding | 18.04 | 0.68 | 0.0319 | 0.9039 |
| *Slc52a3* | chr2-151996511-152009258 | protein coding | 34.83 | 0.53 | 0.0320 | 0.9039 |
| *Tmc6* | chr11-117765988-117782198 | protein coding | 118.19 | 0.42 | 0.0321 | 0.9039 |
| *Gm50403* | chr18-40346169-40351751 | TEC | 2.43 | 2.74 | 0.0321 | 0.9039 |
| *Gm25099* | chr4-117189942-117190057 | snRNA | 1.30 | 2.94 | 0.0322 | NA |
| *Dysf* | chr6-84008590-84211060 | protein coding | 55.33 | 0.40 | 0.0326 | 0.9127 |
| *Tspan9* | chr6-127961396-128143594 | protein coding | 736.87 | 0.36 | 0.0327 | 0.9138 |
| *Tmem26* | chr10-68723646-68782650 | protein coding | 1.62 | 2.32 | 0.0328 | NA |
| *Pygm* | chr19-6384399-6398459 | protein coding | 532.21 | 0.32 | 0.0330 | 0.9163 |
| *Etnppl* | chr3-130617448-130637521 | protein coding | 337.77 | 0.58 | 0.0330 | 0.9163 |
| *Iigp1* | chr18-60376027-60392634 | protein coding | 72.04 | 0.48 | 0.0331 | 0.9165 |
| *St14* | chr9-31089402-31131853 | protein coding | 19.26 | 0.67 | 0.0332 | 0.9165 |
| *Dpy19l2* | chr9-24557047-24696293 | protein coding | 5.97 | 1.13 | 0.0333 | 0.9173 |
| *Fancc* | chr13-63285043-63497278 | protein coding | 175.97 | 0.33 | 0.0334 | 0.9176 |
| *2200002D01Rik* | chr7-29246561-29248466 | protein coding | 1.37 | 2.47 | 0.0336 | NA |
| *Chrna3* | chr9-55010111-55026562 | protein coding | 35.02 | 1.14 | 0.0337 | 0.9176 |
| *Adamts12* | chr15-11064790-11349231 | protein coding | 14.51 | 0.91 | 0.0337 | 0.9176 |
| *Kcne1l* | chrX-142304752-142306294 | protein coding | 85.85 | 0.38 | 0.0337 | 0.9176 |
| *Gm48870* | chr13-9344402-9348425 | TEC | 1.32 | 3.57 | 0.0337 | NA |
| *Tnni3* | chr7-4518305-4524229 | protein coding | 14.70 | 0.80 | 0.0338 | 0.9176 |
| *Gm44770* | chr6-71802888-71804151 | TEC | 12.84 | 0.95 | 0.0338 | 0.9183 |
| *Syt15* | chr14-34219595-34230420 | protein coding | 28.12 | 0.69 | 0.0340 | 0.9207 |
| *Gbp10* | chr5-105215699-105239533 | protein coding | 1.56 | 2.80 | 0.0340 | NA |
| *Gm25360* | chr1-72226240-72226430 | snRNA | 1.55 | 3.96 | 0.0340 | NA |
| *9930012K11Rik* | chr14-70154405-70159502 | protein coding | 28.45 | 0.58 | 0.0341 | 0.9215 |
| *Vat1* | chr11-101458745-101466230 | protein coding | 545.49 | 0.32 | 0.0341 | 0.9215 |
| *Mir6948* | chr14-54643049-54643110 | miRNA | 0.82 | 3.54 | 0.0343 | NA |
| *Gm28822* | chr1-23574587-23596664 | lncRNA | 1.65 | 2.54 | 0.0344 | NA |
| *Duxbl1* | chr14-25978577-25990088 | protein coding | 1.97 | 2.35 | 0.0344 | NA |
| *Col18a1* | chr10-77052178-77166548 | protein coding | 113.17 | 0.58 | 0.0346 | 0.9282 |
| *Ptgfr* | chr3-151796502-151837630 | protein coding | 15.42 | 0.97 | 0.0346 | 0.9283 |
| *Kazald1* | chr19-45075241-45079289 | protein coding | 38.24 | 0.52 | 0.0347 | 0.9288 |
| *Gm3510* | chr5-15923755-15933406 | lncRNA | 73.59 | 0.49 | 0.0347 | 0.9288 |
| *Oas1a* | chr5-120896256-120907521 | protein coding | 12.18 | 0.96 | 0.0348 | 0.9288 |
| *Adgre5* | chr8-83723251-83741326 | protein coding | 249.76 | 0.34 | 0.0350 | 0.9305 |
| *Gm47994* | chr13-38514096-38514875 | processed pseudogene | 0.92 | 2.99 | 0.0353 | NA |
| *Dhh* | chr15-98891152-98898540 | protein coding | 2.46 | 2.07 | 0.0354 | 0.9319 |
| *Hacd4* | chr4-88396144-88438928 | protein coding | 66.13 | 0.43 | 0.0356 | 0.9331 |
| *Gm29480* | chr1-92842177-92848771 | lncRNA | 3.34 | 1.54 | 0.0356 | 0.9331 |
| *Tfap2b* | chr1-19208914-19238576 | protein coding | 27.80 | 1.15 | 0.0356 | 0.9333 |
| *Rhobtb1* | chr10-69151434-69291791 | protein coding | 131.17 | 0.32 | 0.0357 | 0.9333 |
| *E130317F20Rik* | chr10-79851381-79854971 | lncRNA | 86.48 | 0.34 | 0.0357 | 0.9333 |
| *B230323A14Rik* | chr9-69758740-69830845 | lncRNA | 4.47 | 2.15 | 0.0358 | 0.9333 |
| *Jak3* | chr8-71676296-71690575 | protein coding | 137.02 | 0.31 | 0.0360 | 0.9350 |
| *Gm15471* | chr3-103803129-103808439 | lncRNA | 1.98 | 2.68 | 0.0362 | NA |
| *Efemp1* | chr11-28853204-28926743 | protein coding | 255.10 | 0.37 | 0.0363 | 0.9350 |
| *Tinagl1* | chr4-130164454-130175122 | protein coding | 113.04 | 0.43 | 0.0363 | 0.9350 |
| *Gm38085* | chr2-4103877-4106938 | lncRNA | 1.13 | 3.84 | 0.0365 | NA |
| *Angptl4* | chr17-33773750-33781830 | protein coding | 28.42 | 0.54 | 0.0366 | 0.9350 |
| *Gm45102* | chr7-67759317-67760173 | TEC | 6.20 | 1.12 | 0.0366 | 0.9350 |
| *Smim9* | chrX-75146056-75163770 | protein coding | 1.31 | 2.70 | 0.0366 | NA |
| *Osbpl5* | chr7-143688762-143756985 | protein coding | 398.66 | 0.35 | 0.0367 | 0.9350 |
| *Lrrc3* | chr10-77897575-77902536 | protein coding | 149.32 | 0.33 | 0.0367 | 0.9350 |
| *Sorbs3* | chr14-70180468-70211989 | protein coding | 683.71 | 0.33 | 0.0370 | 0.9374 |
| *Fut2* | chr7-45648591-45666394 | protein coding | 3.02 | 1.79 | 0.0371 | 0.9389 |
| *Gm2447* | chr3-52739716-52776656 | lncRNA | 1.37 | 2.99 | 0.0371 | NA |
| *Gm45609* | chr2-155074181-155099510 | lncRNA | 5.15 | 1.38 | 0.0373 | 0.9393 |
| *9330161L09Rik* | chr12-103406822-103407976 | TEC | 6.48 | 1.12 | 0.0374 | 0.9393 |
| *5730405O15Rik* | chrX-13042015-13045297 | lncRNA | 30.53 | 0.56 | 0.0374 | 0.9393 |
| *Gm17146* | chr3-88722090-88722670 | lncRNA | 6.47 | 1.13 | 0.0377 | 0.9393 |
| *Foxr2* | chrX-153118786-153132861 | protein coding | 12.34 | 0.81 | 0.0377 | 0.9393 |
| *Lgr5* | chr10-115450311-115587780 | protein coding | 41.22 | 0.62 | 0.0378 | 0.9393 |
| *Dusp27* | chr1-166098148-166127922 | protein coding | 9.18 | 0.92 | 0.0378 | 0.9393 |
| *Gm4779* | chrX-101790053-101794423 | protein coding | 12.81 | 1.14 | 0.0378 | 0.9393 |
| *Sox14* | chr9-99874106-99876170 | protein coding | 9.30 | 1.47 | 0.0378 | 0.9393 |
| *4930572G02Rik* | chr14-48537991-48571888 | lncRNA | 4.30 | 1.37 | 0.0379 | 0.9407 |
| *Epsti1* | chr14-77904239-78002657 | protein coding | 24.25 | 0.71 | 0.0380 | 0.9407 |
| *Gm13123* | chr4-144247777-144248268 | processed pseudogene | 1.83 | 3.97 | 0.0380 | NA |
| *Evpl* | chr11-116220559-116238077 | protein coding | 42.03 | 0.52 | 0.0380 | 0.9409 |
| *Tap1* | chr17-34187553-34197225 | protein coding | 109.10 | 0.40 | 0.0382 | 0.9417 |
| *Pdp1* | chr4-11958184-11966452 | protein coding | 1540.46 | 0.37 | 0.0383 | 0.9431 |
| *Abi3* | chr11-95830074-95842476 | protein coding | 120.60 | 0.40 | 0.0383 | 0.9434 |
| *Tns1* | chr1-73910231-74124449 | protein coding | 795.53 | 0.31 | 0.0385 | 0.9466 |
| *Plekhg6* | chr6-125362660-125380793 | protein coding | 1.31 | 2.77 | 0.0388 | NA |
| *Dhrs13os* | chr11-78030012-78032665 | lncRNA | 3.48 | 1.81 | 0.0391 | 0.9543 |
| *Cdca7l* | chr12-117804289-117878706 | protein coding | 41.04 | 0.45 | 0.0392 | 0.9543 |
| *Sphk1* | chr11-116530925-116536674 | protein coding | 33.62 | 0.87 | 0.0393 | 0.9543 |
| *Gm36608* | chr19-3689686-3708176 | lncRNA | 3.08 | 1.46 | 0.0396 | 0.9545 |
| *Vsig10* | chr5-117319083-117355005 | protein coding | 55.48 | 0.40 | 0.0396 | 0.9545 |
| *Myh11* | chr16-14194535-14291372 | protein coding | 231.32 | 0.99 | 0.0397 | 0.9545 |
| *Tcf24* | chr1-9960163-9967932 | protein coding | 7.44 | 1.08 | 0.0397 | 0.9546 |
| *Plekha7* | chr7-116123485-116308376 | protein coding | 380.67 | 0.50 | 0.0399 | 0.9551 |
| *Sptbn5* | chr2-120041493-120085678 | protein coding | 24.71 | 0.64 | 0.0399 | 0.9551 |
| *Slc16a12* | chr19-34668403-34747289 | protein coding | 48.87 | 0.44 | 0.0403 | 0.9577 |
| *Foxd1* | chr13-98354242-98359305 | protein coding | 23.19 | 0.77 | 0.0403 | 0.9577 |
| *Brinp3* | chr1-146494760-146902472 | protein coding | 244.65 | 0.37 | 0.0404 | 0.9577 |
| *Ptgds* | chr2-25466709-25470046 | protein coding | 9190.69 | 1.01 | 0.0405 | 0.9577 |
| *Mxra8* | chr4-155839680-155844088 | protein coding | 154.35 | 0.35 | 0.0405 | 0.9577 |
| *Gm49932* | chr17-66231949-66236369 | lncRNA | 3.10 | 1.70 | 0.0406 | 0.9577 |
| *Tcf19* | chr17-35512734-35516824 | protein coding | 54.12 | 0.38 | 0.0406 | 0.9577 |
| *Gm36189* | chr6-88882655-88883177 | processed pseudogene | 1.08 | 2.81 | 0.0407 | NA |
| *Pik3c2g* | chr6-139587221-139969284 | protein coding | 6.83 | 1.20 | 0.0407 | 0.9577 |
| *Adgrg2* | chrX-160390690-160498070 | protein coding | 39.26 | 0.48 | 0.0408 | 0.9577 |
| *Irx2* | chr13-72628820-72634198 | protein coding | 17.44 | 1.40 | 0.0410 | 0.9577 |
| *St8sia2* | chr7-73939119-74013690 | protein coding | 84.65 | 0.50 | 0.0410 | 0.9577 |
| *Mfap4* | chr11-61485431-61488900 | protein coding | 88.54 | 0.46 | 0.0411 | 0.9577 |
| *Tagln* | chr9-45929619-45936058 | protein coding | 188.60 | 0.83 | 0.0411 | 0.9577 |
| *Ptgir* | chr7-16906490-16910905 | protein coding | 1.49 | 2.59 | 0.0414 | NA |
| *Hmcn1* | chr1-150562524-150993435 | protein coding | 111.30 | 0.52 | 0.0415 | 0.9640 |
| *Dbf4* | chr5-8396973-8422716 | protein coding | 33.01 | 0.49 | 0.0417 | 0.9659 |
| *Gm6505* | chr3-28764713-28765360 | processed pseudogene | 1.95 | 2.23 | 0.0419 | NA |
| *Rgl3* | chr9-21968711-21989446 | protein coding | 89.20 | 0.37 | 0.0420 | 0.9659 |
| *Arhgap6* | chrX-168795099-169304435 | protein coding | 56.73 | 0.46 | 0.0421 | 0.9659 |
| *Tekt5* | chr16-10357948-10395490 | protein coding | 93.91 | 0.78 | 0.0423 | 0.9659 |
| *Irf6* | chr1-193153111-193172023 | protein coding | 15.61 | 0.83 | 0.0424 | 0.9659 |
| *Uaca* | chr9-60794542-60880370 | protein coding | 183.19 | 0.46 | 0.0424 | 0.9659 |
| *Gm44708* | chr7-57775429-57777665 | TEC | 3.46 | 1.53 | 0.0428 | 0.9659 |
| *Ip6k3* | chr17-27143965-27167795 | protein coding | 9.16 | 1.10 | 0.0428 | 0.9659 |
| *Cubn* | chr2-13276338-13491813 | protein coding | 18.31 | 1.03 | 0.0429 | 0.9659 |
| *Gm8066* | chr5-137510253-137517501 | lncRNA | 38.06 | 0.57 | 0.0430 | 0.9659 |
| *Rhov* | chr2-119269201-119271272 | protein coding | 70.08 | 0.34 | 0.0430 | 0.9659 |
| *Adamtsl5* | chr10-80339110-80348412 | protein coding | 88.81 | 0.41 | 0.0430 | 0.9659 |
| *Mms22l* | chr4-24496451-24602950 | protein coding | 37.25 | 0.47 | 0.0431 | 0.9659 |
| *Gm36849* | chr7-136353364-136356270 | lncRNA | 1.64 | 2.39 | 0.0431 | NA |
| *Efna5* | chr17-62604184-62881317 | protein coding | 81.56 | 0.43 | 0.0432 | 0.9659 |
| *Tnfrsf11a* | chr1-105780718-105847981 | protein coding | 51.70 | 0.44 | 0.0432 | 0.9659 |
| *Ppp1r26* | chr2-28446800-28455508 | protein coding | 344.55 | 0.31 | 0.0433 | 0.9659 |
| *Wnt9b* | chr11-103727364-103749821 | protein coding | 36.77 | 1.46 | 0.0435 | 0.9659 |
| *Ston1* | chr17-88597684-88662586 | protein coding | 96.90 | 0.43 | 0.0436 | 0.9659 |
| *Syt6* | chr3-103575231-103645569 | protein coding | 174.72 | 0.37 | 0.0436 | 0.9659 |
| *Gja4* | chr4-127311421-127314039 | protein coding | 38.49 | 0.56 | 0.0436 | 0.9659 |
| *Fendrr* | chr8-121054882-121083110 | lncRNA | 25.05 | 0.63 | 0.0439 | 0.9659 |
| *Rcn3* | chr7-45082913-45092221 | protein coding | 136.81 | 0.47 | 0.0439 | 0.9659 |
| *Zic4* | chr9-91362413-91389348 | protein coding | 138.98 | 0.64 | 0.0441 | 0.9659 |
| *Kdm4d* | chr9-14462548-14500482 | protein coding | 21.27 | 0.58 | 0.0441 | 0.9659 |
| *Ttll9* | chr2-152962485-153008482 | protein coding | 20.26 | 0.82 | 0.0442 | 0.9659 |
| *Etnk2* | chr1-133363572-133380336 | protein coding | 33.21 | 0.68 | 0.0442 | 0.9659 |
| *Dmbx1* | chr4-115915119-115939926 | protein coding | 9.93 | 1.04 | 0.0444 | 0.9659 |
| *Il4ra* | chr7-125552120-125579474 | protein coding | 96.08 | 0.33 | 0.0446 | 0.9659 |
| *Lrrc32* | chr7-98489283-98502181 | protein coding | 44.48 | 0.55 | 0.0447 | 0.9660 |
| *Gm24616* | chr16-23111617-23111755 | snoRNA | 2.35 | 2.05 | 0.0448 | 0.9660 |
| *Gm8983* | chr14-56865413-56866355 | processed pseudogene | 4.18 | 1.33 | 0.0449 | 0.9666 |
| *Slc7a11* | chr3-49892526-50443614 | protein coding | 1052.07 | 0.47 | 0.0452 | 0.9670 |
| *3110080E11Rik* | chr8-16912706-16916182 | TEC | 2.70 | 2.20 | 0.0453 | 0.9670 |
| *Gm12185* | chr11-48904656-48992226 | protein coding | 3.76 | 1.50 | 0.0455 | 0.9670 |
| *Oxgr1* | chr14-120019585-120042435 | protein coding | 4.46 | 1.33 | 0.0460 | 0.9670 |
| *Gm30655* | chr13-63575051-63582562 | lncRNA | 7.71 | 1.01 | 0.0460 | 0.9670 |
| *Gper1* | chr5-139423151-139427800 | protein coding | 53.66 | 0.54 | 0.0463 | 0.9670 |
| *Brca1* | chr11-101488764-101551955 | protein coding | 35.63 | 0.45 | 0.0465 | 0.9670 |
| *Hmgcs2* | chr3-98280435-98310738 | protein coding | 170.82 | 0.38 | 0.0466 | 0.9670 |
| *Tmie* | chr9-110865711-110880113 | protein coding | 64.53 | 0.46 | 0.0468 | 0.9670 |
| *Kcnt1* | chr2-25863734-25918273 | protein coding | 1139.18 | 0.40 | 0.0469 | 0.9670 |
| *Gm50394* | chr19-53528609-53529655 | lncRNA | 8.22 | 1.05 | 0.0469 | 0.9670 |
| *Gm47590* | chr10-90851099-90854074 | TEC | 1.73 | 2.15 | 0.0470 | NA |
| *Barhl1* | chr2-28907679-28916668 | protein coding | 8.50 | 1.30 | 0.0470 | 0.9670 |
| *Islr* | chr9-58156268-58204306 | protein coding | 321.85 | 0.49 | 0.0473 | 0.9670 |
| *Gpnmb* | chr6-49036546-49070929 | protein coding | 162.17 | 0.43 | 0.0473 | 0.9670 |
| *Gm18890* | chr15-99883640-99884444 | processed pseudogene | 4.22 | 1.22 | 0.0474 | 0.9670 |
| *Lyl1* | chr8-84701449-84704940 | protein coding | 50.74 | 0.44 | 0.0476 | 0.9670 |
| *Sntb1* | chr15-55636388-55906949 | protein coding | 123.88 | 0.48 | 0.0477 | 0.9670 |
| *Gm36736* | chr7-121314810-121393101 | lncRNA | 1.10 | 2.95 | 0.0478 | NA |
| *Slc45a3* | chr1-131962967-131982969 | protein coding | 23.03 | 0.74 | 0.0478 | 0.9670 |
| *Tnfrsf1a* | chr6-125349362-125362484 | protein coding | 227.69 | 0.31 | 0.0479 | 0.9670 |
| *Rab32* | chr10-10545002-10558265 | protein coding | 65.60 | 0.40 | 0.0479 | 0.9670 |
| *Vegfd* | chrX-164373378-164402650 | protein coding | 24.74 | 0.62 | 0.0481 | 0.9670 |
| *Has1* | chr17-17843323-17855205 | protein coding | 6.52 | 1.03 | 0.0482 | 0.9670 |
| *Adgre1* | chr17-57358691-57483527 | protein coding | 167.46 | 0.41 | 0.0482 | 0.9670 |
| *Asic3* | chr5-24413392-24417835 | protein coding | 8.22 | 1.07 | 0.0483 | 0.9670 |
| *Parp10* | chr15-76231174-76243441 | protein coding | 58.73 | 0.40 | 0.0483 | 0.9670 |
| *Gm26885* | chr17-29446919-29491925 | lncRNA | 10.27 | 0.90 | 0.0484 | 0.9670 |
| *C630004L07Rik* | chr4-141238179-141239453 | TEC | 2.74 | 1.46 | 0.0485 | 0.9675 |
| *Adam18* | chr8-24602246-24674755 | protein coding | 15.23 | 0.99 | 0.0487 | 0.9696 |
| *Thbd* | chr2-148404466-148408188 | protein coding | 197.24 | 0.47 | 0.0488 | 0.9714 |
| *Lax1* | chr1-133679091-133690108 | protein coding | 2.84 | 1.87 | 0.0488 | 0.9714 |
| *Tmem91* | chr7-25669139-25675166 | protein coding | 77.59 | 0.68 | 0.0489 | 0.9719 |
| *C330021F23Rik* | chr8-3567990-3584939 | protein coding | 0.92 | 2.97 | 0.0490 | NA |
| *Cdh5* | chr8-104101625-104144511 | protein coding | 272.96 | 0.40 | 0.0492 | 0.9720 |
| *Gbp9* | chr5-105077630-105139539 | protein coding | 98.22 | 0.38 | 0.0492 | 0.9720 |
| *Gm4356* | chr17-28726083-28726700 | protein coding | 5.51 | 1.30 | 0.0494 | 0.9724 |
| *Gm44996* | chr7-89388344-89391323 | TEC | 1.63 | 2.66 | 0.0494 | NA |
| *Ntng1* | chr3-109780040-110144011 | protein coding | 842.80 | 0.68 | 0.0495 | 0.9724 |
| *Gm45477* | chr3-90311518-90317015 | TEC | 1.16 | 2.82 | 0.0496 | NA |
| *Gm15998* | chr3-89315439-89318907 | lncRNA | 14.41 | 0.71 | 0.0498 | 0.9744 |

**Supplementary Table 6D. Downregulated genes in Pool 2 vs Control mice (Log2 Fold Change ≤ -0.3, p-value < 0.05)**.

| Gene symbol | Chromosomal location | Biotype | baseMean | log2FC | pvalue | padj |
| --- | --- | --- | --- | --- | --- | --- |
| *Gm5869* | chr5-86997159-86998415 | processed pseudogene | 25.41 | -4.91 | 2.92E-07 | 0.0035 |
| *Eef1a1-ps1* | chr19-17926623-17928009 | processed pseudogene | 61.11 | -2.10 | 3.40E-07 | 0.0035 |
| *Gm13456* | chr2-40555752-40557116 | processed pseudogene | 218.43 | -2.31 | 1.23E-06 | 0.0084 |
| *Gm29216* | chr1-24615706-24616197 | unprocessed pseudogene | 74.50 | -1.22 | 0.0001 | 0.0938 |
| *Gm49901* | chr17-50114019-50117459 | lncRNA | 2.17 | -4.50 | 0.0003 | 0.1305 |
| *Gm36447* | chr5-150822859-150866601 | lncRNA | 92.72 | -0.87 | 0.0004 | 0.1495 |
| *Gm28661* | chr1-24614885-24615565 | unprocessed pseudogene | 103.29 | -1.21 | 0.0005 | 0.1850 |
| *Rpl19-ps11* | chr8-19492936-19493526 | processed pseudogene | 46.36 | -0.70 | 0.0006 | 0.1970 |
| *Gm28437* | chr1-24613189-24613971 | unprocessed pseudogene | 77.27 | -1.09 | 0.0012 | 0.2890 |
| *Gm26793* | chr7-144894560-144897461 | lncRNA | 4.01 | -3.07 | 0.0016 | 0.3232 |
| *Gm10925* | chr1-24613974-24614651 | unprocessed pseudogene | 127.92 | -1.10 | 0.0017 | 0.3242 |
| *Lamp5* | chr2-136052239-136069917 | protein coding | 1090.04 | -0.45 | 0.0018 | 0.3311 |
| *Arl6ip6* | chr2-53191726-53219220 | protein coding | 186.54 | -0.38 | 0.0021 | 0.3467 |
| *Anapc10* | chr8-79711820-79779059 | protein coding | 238.75 | -0.31 | 0.0022 | 0.3473 |
| *Mrpl32* | chr13-14608184-14613165 | protein coding | 414.89 | -0.31 | 0.0022 | 0.3473 |
| *1700112D23Rik* | chr3-28702667-28781108 | lncRNA | 12.83 | -1.37 | 0.0023 | 0.3531 |
| *Rpl37rt* | chr5-115102923-115110268 | transcribed processed pseudogene | 159.72 | -0.53 | 0.0025 | 0.3705 |
| *Mtrr* | chr13-68560780-68582149 | protein coding | 391.19 | -0.38 | 0.0035 | 0.4403 |
| *Acat3* | chr17-12923813-12940600 | protein coding | 102.88 | -0.43 | 0.0035 | 0.4403 |
| *Immp1l* | chr2-105904638-105965558 | protein coding | 217.95 | -0.43 | 0.0037 | 0.4496 |
| *Rpl7a-ps7* | chr3-129461972-129462747 | processed pseudogene | 6.90 | -1.59 | 0.0039 | 0.4575 |
| *Itprid1* | chr6-55836895-55978735 | protein coding | 53.44 | -0.64 | 0.0039 | 0.4575 |
| *Gm13341* | chr2-22587291-22587962 | unprocessed pseudogene | 75.77 | -1.02 | 0.0042 | 0.4684 |
| *Gm8326* | chr1-46414502-46416932 | processed pseudogene | 5.70 | -1.99 | 0.0044 | 0.4745 |
| *Gm9843* | chr16-76403251-76403652 | processed pseudogene | 54.39 | -0.76 | 0.0048 | 0.4898 |
| *Eef2-ps2* | chr4-143242024-143243368 | processed pseudogene | 52.04 | -1.09 | 0.0049 | 0.4952 |
| *Rps27rt* | chr9-114982366-114982739 | protein coding | 39.32 | -0.92 | 0.0050 | 0.4993 |
| *Mterf1b* | chr5-4192367-4197651 | protein coding | 37.62 | -0.67 | 0.0054 | 0.5149 |
| *Gm4204* | chr1-135231995-135233225 | processed pseudogene | 91.03 | -0.43 | 0.0055 | 0.5228 |
| *Dlx1as* | chr2-71516454-71537891 | lncRNA | 171.93 | -0.38 | 0.0058 | 0.5358 |
| *Gm20754* | chr3-73066316-73594830 | lncRNA | 109.05 | -0.39 | 0.0059 | 0.5358 |
| *Gm10086* | chr5-108517179-108517532 | processed pseudogene | 9.36 | -1.39 | 0.0062 | 0.5461 |
| *Gm10443* | chr6-86334031-86334240 | processed pseudogene | 37.50 | -0.86 | 0.0065 | 0.5461 |
| *Gm43712* | chr3-90126767-90127057 | unprocessed pseudogene | 13.31 | -1.11 | 0.0065 | 0.5461 |
| *Med21* | chr6-146642547-146650732 | protein coding | 413.00 | -0.34 | 0.0065 | 0.5461 |
| *Llph* | chr10-120227070-120232582 | protein coding | 179.51 | -0.37 | 0.0067 | 0.5497 |
| *Gm15459* | chr5-5781615-5783555 | processed pseudogene | 151.77 | -0.55 | 0.0068 | 0.5529 |
| *Dlx1* | chr2-71528113-71533981 | protein coding | 369.57 | -0.43 | 0.0072 | 0.5656 |
| *Gm15421* | chr5-22528321-22529244 | transcribed processed pseudogene | 14.19 | -1.01 | 0.0076 | 0.5744 |
| *Foxo6* | chr4-120267079-120287349 | protein coding | 407.49 | -0.54 | 0.0083 | 0.5954 |
| *Gm14303* | chr2-172509521-172509691 | processed pseudogene | 164.56 | -0.78 | 0.0083 | 0.5954 |
| *Slc17a5* | chr9-78536488-78588041 | protein coding | 640.90 | -0.32 | 0.0084 | 0.5965 |
| *Oxnad1* | chr14-32085374-32103202 | protein coding | 336.01 | -0.31 | 0.0087 | 0.6024 |
| *Pon1* | chr6-5168090-5193946 | protein coding | 1.94 | -4.60 | 0.0089 | NA |
| *Gm6392* | chr15-60468124-60469477 | processed pseudogene | 4.03 | -2.31 | 0.0089 | 0.6128 |
| *Gm11960* | chr11-4561780-4564637 | transcribed processed pseudogene | 5.34 | -1.66 | 0.0095 | 0.6341 |
| *Eif4e3* | chr6-99625135-99666771 | protein coding | 343.63 | -0.32 | 0.0095 | 0.6341 |
| *Zcchc10* | chr11-53324679-53333301 | protein coding | 179.91 | -0.38 | 0.0095 | 0.6341 |
| *Gprc5d* | chr6-135105991-135118283 | protein coding | 2.55 | -3.28 | 0.0101 | 0.6494 |
| *Gm11249* | chr4-67930881-67931739 | processed pseudogene | 5.83 | -1.59 | 0.0102 | 0.6567 |
| *Msrb2* | chr2-19371440-19394976 | protein coding | 658.13 | -0.40 | 0.0103 | 0.6571 |
| *Gm8343* | chr9-37685941-37686934 | processed pseudogene | 1.26 | -3.73 | 0.0105 | NA |
| *Ccdc173* | chr2-69758033-69789575 | protein coding | 70.53 | -0.49 | 0.0106 | 0.6678 |
| *2810407A14Rik* | chr16-87784075-87839293 | lncRNA | 33.04 | -0.63 | 0.0107 | 0.6689 |
| *Lyg2* | chr1-37905923-37916493 | protein coding | 2.85 | -3.51 | 0.0107 | 0.6689 |
| *Gm5441* | chr12-117234521-117337022 | lncRNA | 1.28 | -3.49 | 0.0108 | NA |
| *Gm13783* | chr2-91195906-91202778 | lncRNA | 1.96 | -3.17 | 0.0109 | NA |
| *Gm9794* | chr6-66875321-66875743 | processed pseudogene | 55.14 | -0.82 | 0.0111 | 0.6800 |
| *Pcdha11* | chr18-37005203-37187657 | protein coding | 62.57 | -0.53 | 0.0115 | 0.6938 |
| *Gm15379* | chrX-75458812-75459459 | processed pseudogene | 19.34 | -0.86 | 0.0121 | 0.7194 |
| *Sox1ot* | chr8-12385771-12436768 | lncRNA | 501.42 | -0.36 | 0.0123 | 0.7210 |
| *Gm15772* | chr5-3236431-3236868 | processed pseudogene | 10.71 | -1.12 | 0.0127 | 0.7239 |
| *Siva1* | chr12-112644679-112649149 | protein coding | 151.19 | -0.34 | 0.0130 | 0.7300 |
| *Rpl36-ps2* | chr11-76601319-76601629 | processed pseudogene | 3.04 | -2.41 | 0.0130 | 0.7300 |
| *B230303A05Rik* | chr13-15813548-16023423 | transcribed processed pseudogene | 328.06 | -0.32 | 0.0130 | 0.7300 |
| *Gm44931* | chr7-80603239-80603783 | TEC | 3.19 | -3.06 | 0.0133 | 0.7328 |
| *Gm17750* | chr13-84025297-84064772 | lncRNA | 90.93 | -0.57 | 0.0135 | 0.7328 |
| *Gm5879* | chr6-87764996-87766204 | processed pseudogene | 6.91 | -1.32 | 0.0140 | 0.7426 |
| *Rasl11b* | chr5-74195286-74199481 | protein coding | 1536.90 | -0.56 | 0.0140 | 0.7426 |
| *Gm7308* | chr6-127282063-127282509 | processed pseudogene | 13.13 | -1.00 | 0.0143 | 0.7455 |
| *Rpl38-ps2* | chr6-140746960-140747172 | processed pseudogene | 38.77 | -0.67 | 0.0143 | 0.7455 |
| *Gm12338* | chr11-75599763-75599954 | processed pseudogene | 45.68 | -0.77 | 0.0147 | 0.7542 |
| *Rps16-ps2* | chr5-129128077-129128517 | processed pseudogene | 61.03 | -0.66 | 0.0148 | 0.7543 |
| *Colec11* | chr12-28594173-28623377 | protein coding | 14.14 | -0.91 | 0.0148 | 0.7543 |
| *Rpl31-ps8* | chr15-90764279-90764656 | processed pseudogene | 44.75 | -0.60 | 0.0149 | 0.7543 |
| *Mtln* | chr2-127791388-127792488 | protein coding | 546.42 | -0.35 | 0.0150 | 0.7543 |
| *Gm10180* | chr9-25101404-25101481 | processed pseudogene | 19.37 | -0.91 | 0.0150 | 0.7543 |
| *Gm10076* | chr14-105681828-105682211 | lncRNA | 20.30 | -0.94 | 0.0158 | 0.7694 |
| *AA536875* | chr14-123043282-123265002 | lncRNA | 1.27 | -3.56 | 0.0159 | NA |
| *Gm10036* | chr18-15832794-15833327 | processed pseudogene | 6.94 | -1.46 | 0.0162 | 0.7750 |
| *4933412O06Rik* | chr13-15781664-15802630 | lncRNA | 70.48 | -0.49 | 0.0164 | 0.7811 |
| *Gm48161* | chr13-30370359-30377183 | lncRNA | 4.12 | -1.91 | 0.0167 | 0.7839 |
| *M6pr-ps* | chr3-99500550-99501379 | processed pseudogene | 1.53 | -3.34 | 0.0172 | NA |
| *Duox2* | chr2-122279247-122298449 | protein coding | 3.10 | -3.77 | 0.0173 | 0.7931 |
| *Gm49539* | chr15-85646083-85667675 | lncRNA | 10.43 | -1.33 | 0.0174 | 0.7931 |
| *Creld2* | chr15-88819646-88826683 | protein coding | 294.67 | -0.34 | 0.0174 | 0.7931 |
| *Smim18* | chr8-33742112-33747770 | protein coding | 119.29 | -0.45 | 0.0174 | 0.7931 |
| *Gm28154* | chr1-17615084-17641081 | lncRNA | 20.74 | -0.84 | 0.0175 | 0.7941 |
| *Gm11770* | chr11-120215082-120216993 | lncRNA | 1.65 | -3.19 | 0.0177 | NA |
| *Eif3s6-ps1* | chr11-9737152-9737621 | processed pseudogene | 6.65 | -1.25 | 0.0178 | 0.7994 |
| *Gm10073* | chr8-106573009-106573350 | processed pseudogene | 15.21 | -0.87 | 0.0178 | 0.7994 |
| *Otop1* | chr5-38275972-38304217 | protein coding | 2.91 | -2.57 | 0.0187 | 0.8131 |
| *4921524J17Rik* | chr8-85408759-85432833 | protein coding | 264.09 | -0.30 | 0.0188 | 0.8131 |
| *Dlx5* | chr6-6877805-6882085 | protein coding | 86.66 | -0.51 | 0.0190 | 0.8159 |
| *Gm11541* | chr11-94694498-94704499 | protein coding | 1.32 | -3.15 | 0.0192 | NA |
| *Gm9385* | chr9-116040593-116041064 | processed pseudogene | 44.30 | -0.57 | 0.0193 | 0.8161 |
| *Pfn3* | chr13-55414688-55415232 | protein coding | 1.11 | -3.60 | 0.0193 | NA |
| *Stc1* | chr14-69029238-69041804 | protein coding | 68.49 | -0.56 | 0.0197 | 0.8162 |
| *Bcl2a1a* | chr9-88956900-88962419 | protein coding | 3.84 | -1.80 | 0.0199 | 0.8162 |
| *Rps26-ps1* | chr8-107439131-107439579 | transcribed processed pseudogene | 16.13 | -0.94 | 0.0206 | 0.8162 |
| *Gm4833* | chr18-8051203-8051992 | processed pseudogene | 5.13 | -1.66 | 0.0206 | 0.8162 |
| *Crh* | chr3-19693401-19695396 | protein coding | 29.72 | -0.65 | 0.0209 | 0.8162 |
| *Mettl18* | chr1-163994889-163997243 | protein coding | 102.23 | -0.33 | 0.0209 | 0.8162 |
| *Egr2* | chr10-67535475-67542188 | protein coding | 42.60 | -1.60 | 0.0212 | 0.8213 |
| *Gm13485* | chr2-50547914-50552258 | lncRNA | 0.94 | -3.50 | 0.0212 | NA |
| *Calm2* | chr17-87433412-87446935 | protein coding | 38148.00 | -0.32 | 0.0212 | 0.8213 |
| *Rps6-ps2* | chr8-88806391-88807137 | processed pseudogene | 3.53 | -1.79 | 0.0221 | 0.8377 |
| *Gm6444* | chr5-123066242-123066699 | processed pseudogene | 11.58 | -1.09 | 0.0223 | 0.8428 |
| *Ifit1bl2* | chr19-34617049-34640743 | protein coding | 13.35 | -0.89 | 0.0229 | 0.8545 |
| *Gm9521* | chr7-18229777-18231292 | processed pseudogene | 1.54 | -3.98 | 0.0229 | NA |
| *Neurod6* | chr6-55677822-55681263 | protein coding | 6565.21 | -0.40 | 0.0229 | 0.8545 |
| *Gm12261* | chr11-59577992-59578636 | processed pseudogene | 1.20 | -3.54 | 0.0233 | NA |
| *Gm26760* | chr15-92344359-92378103 | lncRNA | 6.94 | -1.23 | 0.0234 | 0.8564 |
| *Reg3b* | chr6-78370657-78373466 | protein coding | 1.32 | -3.31 | 0.0236 | NA |
| *D930020B18Rik* | chr10-121641588-121693915 | protein coding | 9.74 | -1.02 | 0.0236 | 0.8596 |
| *Inhba* | chr13-16011851-16031621 | protein coding | 275.92 | -0.40 | 0.0238 | 0.8613 |
| *Snord104* | chr11-106500993-106501062 | snoRNA | 24.86 | -0.68 | 0.0239 | 0.8626 |
| *Gm19196* | chr12-21543307-21554521 | transcribed_unprocessed pseudogene | 81.55 | -0.47 | 0.0240 | 0.8626 |
| *Chrm5* | chr2-112479171-112480769 | protein coding | 44.52 | -0.73 | 0.0245 | 0.8626 |
| *Axdnd1* | chr1-156323509-156421159 | protein coding | 10.42 | -0.95 | 0.0245 | 0.8626 |
| *N4bp3* | chr11-51643063-51650842 | protein coding | 340.25 | -0.44 | 0.0246 | 0.8626 |
| *Avpr1b* | chr1-131599239-131612000 | protein coding | 40.65 | -1.15 | 0.0247 | 0.8626 |
| *Lin37* | chr7-30555441-30559838 | protein coding | 381.86 | -0.31 | 0.0250 | 0.8645 |
| *Gm1818* | chr12-48555308-48559885 | unprocessed pseudogene | 5.34 | -1.40 | 0.0256 | 0.8751 |
| *C230062I16Rik* | chr7-29953621-29970352 | transcribed_unprocessed pseudogene | 86.39 | -0.45 | 0.0262 | 0.8775 |
| *Gm10250* | chr15-5120654-5121135 | processed pseudogene | 35.51 | -0.68 | 0.0264 | 0.8775 |
| *Gm5781* | chr10-116972766-116973208 | processed pseudogene | 29.61 | -0.91 | 0.0265 | 0.8775 |
| *Gm9616* | chr13-3298887-3299667 | processed pseudogene | 45.34 | -0.62 | 0.0266 | 0.8775 |
| *Myo1h* | chr5-114289166-114365357 | protein coding | 72.17 | -0.66 | 0.0267 | 0.8775 |
| *Gm5513* | chr19-14959549-14960766 | processed pseudogene | 1.43 | -3.84 | 0.0269 | NA |
| *Gm15464* | chr1-60117255-60117685 | processed pseudogene | 0.82 | -3.74 | 0.0270 | NA |
| *Bc1* | chr7-144914470-144914637 | lncRNA | 58946.80 | -0.84 | 0.0272 | 0.8775 |
| *Gm33027* | chr7-129398102-129403090 | lncRNA | 23.59 | -0.67 | 0.0275 | 0.8775 |
| *Syce2* | chr8-84872111-84888221 | protein coding | 129.85 | -0.68 | 0.0276 | 0.8775 |
| *Glrx3* | chr7-137437614-137468594 | protein coding | 1248.44 | -0.31 | 0.0276 | 0.8775 |
| *Aard* | chr15-52040107-52045722 | protein coding | 40.22 | -0.53 | 0.0278 | 0.8793 |
| *Gm23301* | chr18-75001455-75001519 | snoRNA | 5.91 | -1.32 | 0.0279 | 0.8800 |
| *Gm12254* | chr11-58508791-58509261 | processed pseudogene | 9.45 | -1.21 | 0.0280 | 0.8807 |
| *Gm18283* | chr15-76933705-76935060 | processed pseudogene | 1.60 | -2.92 | 0.0280 | NA |
| *AA387883* | chr19-52923181-52941209 | lncRNA | 43.35 | -0.46 | 0.0282 | 0.8808 |
| *Htr1a* | chr13-105443639-105448122 | protein coding | 1063.24 | -0.43 | 0.0283 | 0.8808 |
| *Nxph2* | chr2-23321246-23401973 | protein coding | 17.91 | -0.87 | 0.0283 | 0.8808 |
| *Ang* | chr14-51091150-51102009 | protein coding | 29.42 | -0.61 | 0.0284 | 0.8808 |
| *Gm5805* | chr15-81972390-81972845 | processed pseudogene | 18.34 | -0.86 | 0.0289 | 0.8840 |
| *Dlx6os1* | chr6-6820189-6871592 | lncRNA | 519.33 | -0.32 | 0.0294 | 0.8886 |
| *2700069I18Rik* | chr3-5177586-5220841 | lncRNA | 16.75 | -0.74 | 0.0295 | 0.8886 |
| *H2ac6* | chr13-23681467-23683948 | protein coding | 36.61 | -0.54 | 0.0295 | 0.8886 |
| *Cfap100* | chr6-90403479-90428797 | protein coding | 362.12 | -0.42 | 0.0298 | 0.8938 |
| *Gm17709* | chr8-121972727-121973822 | processed pseudogene | 2.86 | -1.87 | 0.0300 | 0.8938 |
| *Cbx3-ps6* | chr14-73955000-73955551 | processed pseudogene | 1.64 | -2.93 | 0.0305 | NA |
| *Atf4* | chr15-80255184-80257541 | protein coding | 2466.34 | -0.32 | 0.0306 | 0.8979 |
| *Gm6543* | chr5-21598740-21599547 | processed pseudogene | 1.18 | -2.93 | 0.0308 | NA |
| *Gm15501* | chr7-93178983-93184176 | transcribed processed pseudogene | 22.28 | -0.69 | 0.0310 | 0.8999 |
| *Gm2308* | chr7-42125200-42126203 | processed pseudogene | 3.13 | -1.91 | 0.0312 | 0.8999 |
| *Ndufs5-ps* | chr16-42955631-42955949 | processed pseudogene | 35.89 | -0.61 | 0.0314 | 0.9039 |
| *Zbtb3* | chr19-8802555-8804846 | protein coding | 92.50 | -0.33 | 0.0317 | 0.9039 |
| *Gm9755* | chr8-67514235-67515590 | processed pseudogene | 5.19 | -1.32 | 0.0321 | 0.9048 |
| *Gm4811* | chr13-64927942-64929161 | processed pseudogene | 1.50 | -3.66 | 0.0324 | NA |
| *Khdrbs3* | chr15-68928420-69101211 | protein coding | 4760.44 | -0.37 | 0.0325 | 0.9104 |
| *Gm6473* | chr1-30769687-30771815 | unprocessed pseudogene | 1.32 | -2.91 | 0.0327 | NA |
| *Gm40466* | chr8-20001099-20008736 | unprocessed pseudogene | 22.42 | -0.67 | 0.0327 | 0.9138 |
| *Dusp6* | chr10-99263231-99267489 | protein coding | 1547.36 | -0.46 | 0.0330 | 0.9163 |
| *Dctpp1* | chr7-127256959-127260709 | protein coding | 150.49 | -0.35 | 0.0336 | 0.9176 |
| *D830050J10Rik* | chr6-115675968-115677780 | lncRNA | 24.49 | -0.61 | 0.0349 | 0.9304 |
| *1700030C14Rik* | chr2-152652883-152654330 | lncRNA | 4.06 | -1.54 | 0.0350 | 0.9305 |
| *H3c15* | chr3-96238108-96239127 | protein coding | 36.39 | -0.60 | 0.0351 | 0.9305 |
| *Acot13* | chr13-24817948-24831540 | protein coding | 1443.08 | -0.32 | 0.0354 | 0.9319 |
| *Gm5787* | chr8-36372596-36373633 | processed pseudogene | 1.24 | -2.79 | 0.0354 | NA |
| *Gm11539* | chr11-96248633-96249189 | processed pseudogene | 10.41 | -1.09 | 0.0355 | 0.9327 |
| *D830035M03Rik* | chr9-120490532-120492547 | lncRNA | 18.89 | -0.71 | 0.0360 | 0.9350 |
| *Gm5865* | chr5-44405957-44406593 | processed pseudogene | 18.10 | -0.66 | 0.0361 | 0.9350 |
| *Ndufaf2* | chr13-108002715-108158623 | protein coding | 284.27 | -0.36 | 0.0362 | 0.9350 |
| *Gm9349* | chr17-74329533-74329888 | processed pseudogene | 4.62 | -1.49 | 0.0363 | 0.9350 |
| *Eid3* | chr10-82866626-82867930 | protein coding | 2.65 | -2.15 | 0.0364 | 0.9350 |
| *Glt8d2* | chr10-82650433-82690650 | protein coding | 519.52 | -0.36 | 0.0367 | 0.9350 |
| *Rpl10-ps3* | chr9-50344201-50344968 | protein coding | 15.99 | -0.83 | 0.0368 | 0.9350 |
| *Gm38843* | chr6-82803769-82805083 | lncRNA | 7.26 | -1.11 | 0.0375 | 0.9393 |
| *Gm7926* | chr18-30419686-30420060 | processed pseudogene | 2.85 | -1.73 | 0.0377 | 0.9393 |
| *Ido2* | chr8-24531892-24576333 | protein coding | 60.59 | -0.48 | 0.0381 | 0.9412 |
| *Nfil3* | chr13-52967209-52981073 | protein coding | 191.39 | -0.32 | 0.0387 | 0.9486 |
| *Gm3839* | chr14-11280735-11356726 | protein coding | 4.29 | -1.43 | 0.0391 | 0.9543 |
| *E130006D01Rik* | chr5-111734284-111761725 | lncRNA | 14.82 | -0.76 | 0.0392 | 0.9543 |
| *Gm35037* | chr7-3451357-3475020 | lncRNA | 47.44 | -0.76 | 0.0393 | 0.9543 |
| *Gm2225* | chr8-78486034-78486410 | processed pseudogene | 8.47 | -1.36 | 0.0394 | 0.9543 |
| *Atp5pb-ps* | chr11-55118101-55118795 | processed pseudogene | 8.45 | -0.95 | 0.0399 | 0.9551 |
| *D5Ertd615e* | chr5-45009171-45424402 | lncRNA | 1.06 | -3.00 | 0.0406 | NA |
| *Egr1* | chr18-34859823-34864984 | protein coding | 2947.66 | -0.51 | 0.0411 | 0.9577 |
| *Rpl17-ps5* | chr5-22612628-22613182 | processed pseudogene | 5.86 | -1.19 | 0.0419 | 0.9659 |
| *Gm43154* | chr6-31605190-31798053 | lncRNA | 71.28 | -0.52 | 0.0423 | 0.9659 |
| *Gm13498* | chr2-50909684-50911849 | processed pseudogene | 1.66 | -3.02 | 0.0428 | NA |
| *B930059L03Rik* | chr12-110590512-110592700 | lncRNA | 35.71 | -0.48 | 0.0428 | 0.9659 |
| *Pid1* | chr1-84036296-84364180 | protein coding | 1058.29 | -0.36 | 0.0430 | 0.9659 |
| *Pnp2* | chr14-50955992-50964749 | protein coding | 8.48 | -0.89 | 0.0431 | 0.9659 |
| *Fgf2* | chr3-37348346-37410108 | protein coding | 253.97 | -0.42 | 0.0432 | 0.9659 |
| *Acyp2* | chr11-30505991-30649587 | protein coding | 335.73 | -0.36 | 0.0433 | 0.9659 |
| *8430426J06Rik* | chr15-81242657-81312866 | lncRNA | 7.38 | -1.20 | 0.0436 | 0.9659 |
| *Gm1141* | chrX-71920947-71940870 | protein coding | 13.02 | -0.78 | 0.0439 | 0.9659 |
| *Gm22637* | chr14-45461363-45461645 | miscRNA | 6.26 | -1.30 | 0.0440 | 0.9659 |
| *Abracl* | chr10-18011260-18023288 | protein coding | 429.42 | -0.46 | 0.0442 | 0.9659 |
| *Gemin6* | chr17-80224441-80228497 | protein coding | 94.21 | -0.30 | 0.0443 | 0.9659 |
| *4930461C15Rik* | chr16-58404878-58408276 | lncRNA | 3.00 | -1.59 | 0.0450 | 0.9670 |
| *Gm15975* | chr5-55624190-55625591 | processed pseudogene | 2.75 | -1.95 | 0.0454 | 0.9670 |
| *1700001L19Rik* | chr13-68597421-68614231 | protein coding | 127.44 | -0.55 | 0.0457 | 0.9670 |
| *Mxd3* | chr13-55325168-55329823 | protein coding | 22.14 | -0.80 | 0.0458 | 0.9670 |
| *Gm10222* | chr1-24612407-24612700 | unprocessed pseudogene | 4.92 | -1.39 | 0.0463 | 0.9670 |
| *Pcdhb6* | chr18-37333921-37337674 | protein coding | 88.24 | -0.33 | 0.0463 | 0.9670 |
| *Snord98* | chr10-62765576-62765640 | snoRNA | 3.67 | -1.62 | 0.0464 | 0.9670 |
| *Ndufs6b* | chr7-81161397-81161744 | processed pseudogene | 5.93 | -1.38 | 0.0469 | 0.9670 |
| *Chrm4* | chr2-91927249-91928688 | protein coding | 224.94 | -0.47 | 0.0470 | 0.9670 |
| *Gm6170* | chr1-128520073-128521460 | processed pseudogene | 8.12 | -1.16 | 0.0470 | 0.9670 |
| *Rpp21* | chr17-36255645-36258069 | protein coding | 271.53 | -0.31 | 0.0472 | 0.9670 |
| *Gm4691* | chr6-128202150-128203276 | transcribed processed pseudogene | 8.07 | -1.04 | 0.0476 | 0.9670 |
| *Gm12901* | chr4-123258046-123258904 | processed pseudogene | 2.15 | -2.60 | 0.0476 | 0.9670 |
| *Suv39h2* | chr2-3455815-3475031 | protein coding | 119.86 | -0.35 | 0.0476 | 0.9670 |
| *Lefty1* | chr1-180935022-180938400 | protein coding | 415.55 | -0.76 | 0.0479 | 0.9670 |
| *Capn11* | chr17-45630204-45659325 | protein coding | 44.77 | -1.76 | 0.0483 | 0.9670 |
| *Gm44007* | chr6-124381972-124384160 | TEC | 0.72 | -3.32 | 0.0484 | NA |
| *F630042J09Rik* | chr13-67278577-67283361 | lncRNA | 8.45 | -1.16 | 0.0484 | 0.9670 |
| *Gm6788* | chr19-28763161-28763430 | processed pseudogene | 5.08 | -1.24 | 0.0491 | 0.9720 |
| *Dpy30* | chr17-74299474-74323944 | protein coding | 391.93 | -0.35 | 0.0492 | 0.9720 |
| *Npy* | chr6-49822710-49829507 | protein coding | 1069.19 | -0.36 | 0.0493 | 0.9720 |
| *Gm5855* | chr3-130929366-130930670 | processed pseudogene | 4.24 | -1.53 | 0.0494 | 0.9724 |
| *Il6* | chr5-30013114-30019981 | protein coding | 1.06 | -2.78 | 0.0494 | NA |

**Supplementary Table 7A. Common upregulated genes in Pool 1 and Pool 2 vs Control mice (Log2 Fold Change ≥ 0.3, p-value < 0.05)**.

| Gene symbol |  | Gene symbol |
| --- | --- | --- |
| *5730405O15Rik* |  | *Gm42918* |
| *Abca12* |  | *Gm43429* |
| *Adamtsl5* |  | *Gm44798* |
| *Ank1* |  | *Gm45178* |
| *Bmf* |  | *Gm48086* |
| *Brca1* |  | *Gm49674* |
| *Btc* |  | *Gm50394* |
| *C330011M18Rik* |  | *Helz2* |
| *CT010467.1* |  | *Hmcn2* |
| *Ccdc88b* |  | *Kcna5* |
| *Ccrl2* |  | *Kcnk12* |
| *Coro6* |  | *Lars2* |
| *D7Bwg0826e* |  | *Mcm10* |
| *Dennd3* |  | *Mir344i* |
| *Dio3os* |  | *Mir6236* |
| *Dnmbp* |  | *Mir6240* |
| *E130317F20Rik* |  | *Npsr1* |
| *Gm15351* |  | *Oxgr1* |
| *Gm15564* |  | *Pip5kl1* |
| *Gm17146* |  | *Plekha7* |
| *Gm22009* |  | *Rdh1* |
| *Gm22175* |  | *Rn7sk* |
| *Gm22513* |  | *Rnu12* |
| *Gm23935* |  | *Rny1* |
| *Gm24187* |  | *Rny3* |
| *Gm24245* |  | *Scarna2* |
| *Gm24265* |  | *Siah3* |
| *Gm24270* |  | *Slc52a3* |
| *Gm24601* |  | *Snord17* |
| *Gm25360* |  | *Soat2* |
| *Gm25939* |  | *Sp100* |
| *Gm26796* |  | *Syt2* |
| *Gm26917* |  | *Tacstd2* |
| *Gm27320* |  | *Trim34b* |
| *Gm36736* |  | *Ucn2* |
| *Gm37628* |  | *Vegfd* |
| *Gm38336* |  | *Vwa5b2* |
| *Gm38379* |  | *n-R5-8s1* |
| *Gm42418* |  |  |

**Supplementary Table 7B. Common downregulated genes in Pool 1 and Pool 2 vs Control mice (Log2 Fold Change ≤ -0.3, p-value <0.05)**.

| Gene symbol |  | Gene symbol |
| --- | --- | --- |
| *4921524J17Rik* |  | *Gm5855* |
| *Aard* |  | *Gm5869* |
| *Acyp2* |  | *Gm6170* |
| *Anapc10* |  | *Gm6392* |
| *Creld2* |  | *Gm6444* |
| *Dctpp1* |  | *Gm8326* |
| *Dpy30* |  | *Gm9349* |
| *Eef1a1-ps1* |  | *Gm9521* |
| *F630042J09Rik* |  | *Gm9843* |
| *Gm10076* |  | *H3c15* |
| *Gm10086* |  | *Immp1l* |
| *Gm10222* |  | *Inhba* |
| *Gm10443* |  | *Llph* |
| *Gm10925* |  | *Med21* |
| *Gm12254* |  | *Mrpl32* |
| *Gm12338* |  | *Mterf1b* |
| *Gm12901* |  | *Mtln* |
| *Gm13341* |  | *Pnp2* |
| *Gm13456* |  | *Rpl19-ps11* |
| *Gm14303* |  | *Rpl31-ps8* |
| *Gm15421* |  | *Rpl37rt* |
| *Gm15459* |  | *Rpl38-ps2* |
| *Gm17750* |  | *Rpl7a-ps7* |
| *Gm19196* |  | *Rps16-ps2* |
| *Gm26793* |  | *Rps27rt* |
| *Gm28437* |  | *Rps6-ps2* |
| *Gm28661* |  | *Smim18* |
| *Gm29216* |  | *Zcchc10* |
